# Comparison of Small Biomolecule Ionization and Fragmentation in *Pseudomonas aeruginosa* Using Common MALDI Matrices

**DOI:** 10.1021/jasms.2c00157

**Published:** 2023-01-25

**Authors:** Nathan C. Wamer, Chase N. Morse, Jennifer N. Gadient, Taylor A. Dodson, Eric A. Carlson, Erin G. Prestwich

**Affiliations:** † Department of Medicinal and Biological Chemistry, University of Toledo, Toledo, Ohio 43606, United States; ‡ The College of Natural Sciences and Mathematics, NSM Instrumentation Center, University of Toledo, Toledo, Ohio 43606, United States

## Abstract

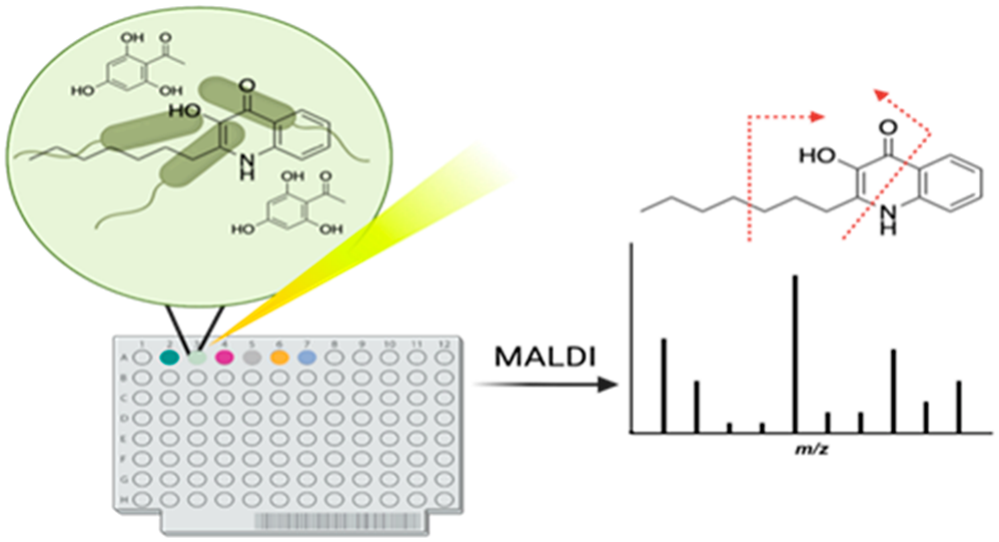

Different bacterial cell surface associated biomolecules can be analyzed by matrix-assisted laser desorption ionization time-of-flight (MALDI-TOF) mass spectrometry and coupled with collision induced dissociation (CID) for identification. *Pseudomonas aeruginosa* is an opportunistic, Gram-negative bacterium that causes acute or chronic biofilm infections. Cells of *P. aeruginosa* communicate through a system of signaling biomolecules known as quorum sensing (QS). The QS system can result in the production of biosurfactant rhamnolipids known to associate and alter the cellular membrane. MALDI-TOF utilizes a variety of matrices that can interact differently with biomolecules for selective ionization. We examined six common matrices to determine the optimal matrix specific to different molecule classes in *P. aeruginosa* associated with cell surfaces. Three major molecule classes (quinolones, rhamnolipids, and phospholipids) were observed to ionize selectively with the different matrices tested. Sodiated and protonated adducts differed between matrices utilized in our study. Isobaric ions were identified as different molecule classes depending on the matrix used. We highlight the role of matrix selection in MALDI-TOF identification of molecules within a complex biological mixture.

## Introduction

Matrix-assisted laser desorption ionization mass spectrometry (MALDI-TOF MS) can be utilized for the identification and characterization of microorganisms.^1,2^ Analysis of MALDI-TOF MS can be used to describe numerous small biomolecules associated with specific microorganisms. Many of these biomolecules play important roles in cellular processes such as cell-to-cell communication, growth, and antibiotics.^3−5^ Specific ions can be fragmented using MALDI-TOF MS coupled with collision induced dissociation (CID). Together, MALDI-TOF MS with CID allows the selection of specified precursor ions for direct fragmentation to aid ion structure identification. As such, MALDI-TOF MS coupled with CID serves as a powerful tool for the direct analysis of small biomolecules associated with microorganisms.

Lipids are crucial for understanding specific microbes for human health. The World Health Organization (WHO) lists carbapenem-resistant *Pseudomonas aeruginosa* as a critical priority pathogen,^6^ making the generation of new antibiotics a high priority.^7^ Antibiotic drug strategies are being developed that target membrane phospholipids for cell lysis.^8,9^ Glycoconjugate vaccines are also being further developed to combat antimicrobial resistance.^10^ The analysis of both the lipid membrane and virulence associated molecules could help elucidate targetable functional mechanisms in complex bacterial systems. The membrane lipid composition of *P. aeruginosa* consists of several different classes such as phosphatidylethanolamines (PEs), phosphatidic acids (PAs), and phosphatidylglycerols (PGs).^8^ Each of these contain two acyl chains that can be differing lengths, which can be either saturated or unsaturated.^8^ These lipid classes also differ based on the functional groups attached to their phosphate headgroup. Lysophospholipids have a single acyl chain that are present in the cell membrane. They can be precursors of other phospholipids and can be produced under stress.^11^

*P. aeruginosa* is regulated by cell-to-cell small molecule communications, known as quorum sensing (QS).^12^ These chemical signals include alkyl quinolones (AQs) that control production of multiple virulence factors.^13−15^ The most notable forms of these classes are Pseudomonas quorum signal (PQS), alkyl hydroxyquinoline N-oxide (AQNO), and alkyl hydroxyquinoline (AHQ).^15^ The hydroxyl group on the quinolone ring and the variable alkyl chain differentiate each of the AQ structures. These structural differences result in varying functionality and activity of each quinolone.^15^ Quorum sensing molecules regulate the self-production of rhamnolipids, which alter cellular interactions.^16^ Rhamnose (Rha) sugars can be conjugated to lipid aliphatic chains which can be of varying lengths. One rhamnose (mono–rhamnolipid) can have one aliphatic chain (Rha–C) or two aliphatic chains (Rha–C–C). Two rhamnose moieties (di–rhamnolipids) can also be conjugated to either one (Rha–Rha–C) or two aliphatic chains (Rha–Rha–C–C).^17^ Analysis of these molecule types in *P. aeruginosa* cultures could provide insight into their functions and their role in proliferation.

Several classes of MALDI matrices have been developed to assist in the ionization of a range of molecules. Matrix selection is a critical component in MALDI-TOF MS,^18^ as different matrices may aid in preferential ionization of the sample and prevent unwanted fragmentation of analytes. Matrices co-crystallize with the sample and absorb energy from the pulsed laser, assisting in analyte ionization. The ratio of matrix to analyte is known to contribute to ionization efficiency.^19^ However, since analyte concentration in whole cell samples is often unknown, this matrix to analyte ratio may need to be optimized. The exact mechanism for matrix-assisted ionization is unclear.^20^ Acidic matrices can donate protons to analytes during positive ion mode; however, these matrices can also form negatively charged ions.^21^ Other methods of ionization can occur during MALDI-TOF analysis.^20,22,23^ The matrix functionality in biological samples could play a role in molecule ionization and ion adduct forms.

Multiple matrices have been shown to ionize biomolecules (including phospholipids,^18^ rhamnolipids,^24^ and quinolones).^25^ In this study, the common MALDI matrices, Super DHB, 2,5-dihydroxybenzoic acid:2-hydroxy-5-methoxybenzoic acid (9:1) (sDHB), 2′,4′,6′-trihydroxyacetophenone (THAP), 5-chloro-2-mercaptobenzothiazole (CMBZT), 9-aminoacradine (9AA), 3-hydroxypicolinic acid (HPA), and α-cyano-4-hydroxy-cinnamic acid (CHCA), were selected for analysis. Typically, sDHB and CHCA are used with MALDI-TOF to analyze lipids, peptides, and proteins.^18,26−29^ Oligonucleotide mixtures are often ionized with HPA.^30^ The relatively neutral acetophenone derivative matrix, THAP, has been applied as a MALDI matrix for oligonucleotides and glycoproteins.^31,32^ The organosulfur matrix, CMBZT, is typically used for peptides, proteins, and lipids.^33^ The moderately basic matrix, 9AA, is typically used in negative mode for lipids and lower molecular weight compounds.^34,35^ The difference in structure and acid–base character of various matrices highlights the importance of surveying molecules of interest with different matrices. Previous studies comparing multiple matrices often focused on a select few molecules. In many cases, these molecules were isolated and purified before analysis.^36−38^ In this study, whole cells containing several small molecular classes were analyzed to understand how matrices differed in their ionization and fragmentation of molecules of interest. Furthermore, many of these matrices can be combined with certain additives to help select and increase ionization efficiency of particular molecular classes.^38,39^ Our analysis focused on the matrices themselves, and as such, additives were not used.

Here, we utilize MALDI-TOF MS coupled with CID fragmentation to analyze and putatively identify major small molecule classes within *P. aeruginosa* planktonic bacteria. Furthermore, the use of common MALDI matrices provides insight into matrix specific ionization and their global coverage of both mass ranges and molecule class identification. Similar sample preparation without additives can demonstrate ionization variability between the matrices. Fragmentation analysis allows isomer and ion adduct identification. This will help provide insight into the ability of each matrix to select and differentiate molecules within a complex biological sample.

## Experimental Section

### Materials

All chemicals applied were not subjected to further purification. Super DHB, 2,5-dihydroxybenzoic acid:2-hydroxy-5-methoxybenzoic acid (9:1) (sDHB, >99.0%), 2′,4′,6′-trihydroxyacetophenone monohydrate (THAP, >95.5%), and 5-chloro-2-mercaptobenzothiazole (CMBZT, >90.0%) were purchased from Sigma-Aldrich (MO, USA), 9-aminoacradine (9AA, >98.0%) was purchased from TCI America (OR, USA), 3-hydroxypicolinic acid (HPA, > 99.9%) was purchased from Chem-Impex International (IL, USA), and α-cyano-4-hydroxy-cinnamic acid (CHCA, >99.0%) was purchased from Fluka-Honeywell (NC, USA). Methanol and acetonitrile (Optima LC/MS grade) were purchased from Fisher Scientific (NH, USA). Peptide Calibration Standard II and MTP 384 Target plate Ground Steel TF were purchased from Bruker (MA, USA). Lipid standards, 1,2-dioleoyl-*sn*-glycero-3-phospho-(1′-*rac*-glycerol) (sodium salt) (PG 18:1 (Δ9-Cis)), 1-palmitoyl-2-oleoyl-*sn*-glycero-3-phosphoethanolamine (PE (18:1/16:0)), 1-palmitoyl-2-oleoyl-*sn*-glycero-3-phosphate (sodium salt) (PA (18:1/16:0)), and 1-oleoyl-2-hydroxy-*sn*-glycero-3-phospho-(1′-*rac*-glycerol) (LPG(18:1)), were purchased from Avanti Polar Lipids (AL, USA). Quinolone standards, 2-heptyl-3-hydroxy-4(1*H*)-quinolone (PQS) and 2-heptyl-4-quinolinoal 1-oxide (HQNO), and lactone standard *N*-hexanoyl-l-homoserine lactone (HSL) were purchased from Cayman Chemical Company (MI, USA). Quinolone standard 2-heptyl-4-quinolone (HHQ), rhamnolipids, 95%(mono–rhamnolipid dominant), and rhamnolipids, 95% (di–rhamnolipid dominant) were purchased from Sigma-Aldrich (MO, USA). Luria–Bertani (LB) broth (Miller) and LB agar (Miller) were purchased from Fisher BioReagents (PA, USA).

### Matrix Preparation

Each matrix solution was made fresh prior to each application. Matrix solutions were prepared for sDHB (50 mg/mL, 50:50, ACN:H_2_O), 9AA (15 mg/mL, MeOH), CMBZT (saturated, 50:50 MeOH:H_2_O), HPA (saturated, H_2_O), THAP (40 mg/mL, 50:50, ACN:H_2_O), and CHCA (saturated, H_2_O). All matrices were prepared without additives.

### Cell Cultures, Culture Conditions, and Standards

*Pseudomonas aeruginosa* UCBPP-PA14 was propagated and maintained in Luria–Bertani (LB) broth (Miller) and LB agar (Miller). Three colonies of *P. aeruginosa* wild-type cells were grown in separate cultures overnight at 37 °C with orbital shaking in LB broth until the stationary phase (14–16 h). An aliquot of 200 μL from each replicate was transferred to a microcentrifuge tube. Cultures were pooled together to ensure sample consistency. The pooled samples (600 μL total) were centrifuged at 16 000*g* at 4 °C for 10 min. Supernatant was removed gently by pipetting, and the pellet was resuspended in 0.9% saline. Standards of phospholipids were resolubilized in chloroform. Rhamnolipid, quinolone, and lactone standards were prepared in a mixture of methanol:0.9% saline (50:50,v/v).

### MALDI Plating

Matrices without sample were spotted in triplicate on a ground steel target plate. Once matrices were dried, 1 μL of either prepared cells or chemical standards was spotted on top of the matrices and dried again. For matrix solutions containing organic solvents (sDHB, THAP, 9AA, and CMBZT), no matrix was applied on top of sample spots to maintain cellular integrity. Samples with CHCA or HPA were spotted using the sandwich method,^40^ which required another 1 μL matrix layer. Standards were spotted onto the plate in the same procedure as the cells for each matrix. All standards were used as a representative fragmentation pattern for the classes of molecules.

### Spectra Collection

Spectra were collected utilizing a Bruker UltrafleXtreme MALDI-TOF/TOF with flexControl software. All experiments were conducted in reflectron positive mode with a frequency-tripled Nd:YAG laser (355 nm). Initial scans (50–1200 *m/z*) were collected using each matrix in triplicate with the same pooled bacterial samples. Ion peaks that appeared in two out of three of the initial scans were further analyzed. Collision induced dissociation (CID) with argon (5 × 10^–6^ mbar) was performed on freshly prepared bacterial samples to putatively identify precursor ions of interest. An ion gate selects the ion of interest for CID fragmentation analysis based on the drift tube time calibrated before every experiment. The instrument was calibrated to less than 5 ppm before every experiment. Calibration for fragmentation analysis was performed with the Peptide Calibration Standard II (*m*/*z* ≈ 700–3500) (Bruker) using 15 mg/mL sDHB suspended in 30% acetonitrile, 70% water, and 0.1% trifluoroacetic acid. The sulfur cluster, S_11_ (*m*/*z* 351.693), was used as an additional calibrant.^41^ Following calibration, CID spectra of the ions of interest were collected. Raw data was imported to FlexAnalysis Bruker software. Matrix specific ions were manually removed for the generation of mass lists using the Snap peak detection algorithm with a signal-to-noise (S/N) threshold set to ≥3.

## Results and Discussion

This work provides different identifications and analyses of cell associated small biomolecules. Generally, MALDI-TOF analysis of bacterial molecules focuses on only one class of molecule and utilizes a few select MALDI matrices.^4,24,42^ Performance of each matrix over three highlighted molecule types (phospholipids, rhamnolipids, and quinolones) and their respected subclasses will be discussed. These results provide a basis for analyzing different molecule classes known to be associated with *P. aeruginosa*. Sample preparation without additives and subsequent analysis allows less bias to understand how each matrix performs under similar conditions with dried droplet sampling.

### MALDI Matrix Preparation and Spotting

This study focused on small signaling molecules and lipids on the cell surface. Thus, for cellular sample consistency, three biological replicates were pooled directly prior to analysis. To maintain the integrity of the signaling molecules and lipids, cellular samples were not washed.^43^ To ensure this, ionized small surface molecules were analyzed both before and after a saline wash. When the samples were washed, less molecules ionized with CHCA, THAP, and CMBZT. After cells were washed and ionized using sDHB, some ions appeared with higher intensity, while in HPA, similar amounts of ions were retained (Figure S1). To properly compare the matrices, all experiments were not washed to maintain the integrity of small molecules at the cell surface. Without the wash step, small peptides and peptide fragments may still be present; however, many peptides were beyond the *m*/*z* 50–1200 range and scope of this analysis. Each matrix was prepared right before analysis to prevent degradation. Additives were not included in matrix solutions. Our intent was to compare ionization and adducts between matrices. Differing matrix concentrations were tested to optimize overall intensity of potential ions of interest. At these concentrations, 9AA, sDHB, and THAP, readily dissolved in their solvent systems. Saturated solutions of CMBZT, HPA, and CHCA were used to promote ionization. Matrix mixtures were centrifuged briefly, and the supernatant was used for spotting. The spotted matrices appeared different on the ground steel plate. When spotted, CHCA and THAP both appeared as white homogeneous spots (Figure S2a,b). Upon analysis, sample ions were found throughout the entire spot. Matrix spots of sDHB and HPA appeared as a ring of crystalline solid (Figure S2c,d)^44^ with ions found predominantly within the outer crystalline ring. Matrix spots for CMBZT and 9AA formed as a dense spot on the sample target plate (white and yellow respectively) (Figure S2e,f) with ions found across the entire sample spot. Distribution of ions throughout sample spots with dried droplet sampling can be irregular and sample dependent. Analyte distribution was recorded for reproducibility and was consistent during the generation of the ion mass list.

### Generation of Ions of Interest

With matrix conditions established, spectra were collected and collated to generate an ion of interest list for each matrix. We first collated a list of ion peaks that had S/N ≥ 3. Similar ion peaks in two out of three spectra for a specific matrix were considered as possible peaks of interest for that matrix. All peaks of interest were then compared to predictive adduct ([M]^+^, [M + H]^+^, [M + Na]^+^, [M + Na + H]^+^, [M + Na – H]^+^ , [M + 2Na]^+^, [M + 2Na + H]^+^, [M + 2Na – H]^+^) ions of small molecules previously observed in *P. aeruginosa*.^8,17,45−48^ All peaks that appeared in two of the three scans and corresponding to known molecules were deemed as our ions of interest and subjected to further fragmentation analysis (Table S1). The number of ion peaks and their observed relative intensities varied between the matrices.

Identification of ions of interest were attempted by CID fragmentation. Only specific precursor ions that have been ionized with the assistance of the matrix can be fragmented by CID. Analysis of CID fragmentation of selected precursor ions provides insight into the chemical structure of the selected ion. All identification presented in this study was putative, and commercial standards were used to gather representative fragmentation patterns (Tables S2–S5). Previous work has shown that fragmentation analysis can be dependent on the MALDI matrix properties including proton affinity.^49^ As such, adducts of commercial standards and their fragmentation could not always be directly compared to ions of interest. Due to these possible differences, commercial standards were used to help guide analysis based on their common fragments (Tables S2–S5). For ionization of precursor ions and identification following CID fragmentation, the required laser power varied between matrices. Typically, CHCA required the lowest laser power for both ion selection and fragmentation, while 9AA typically required the most laser power. In total, 211 ions were found to meet our criteria, outlined in the Experimental Section, for being ions of interest in this study (Table S1).

**Figure 1:**
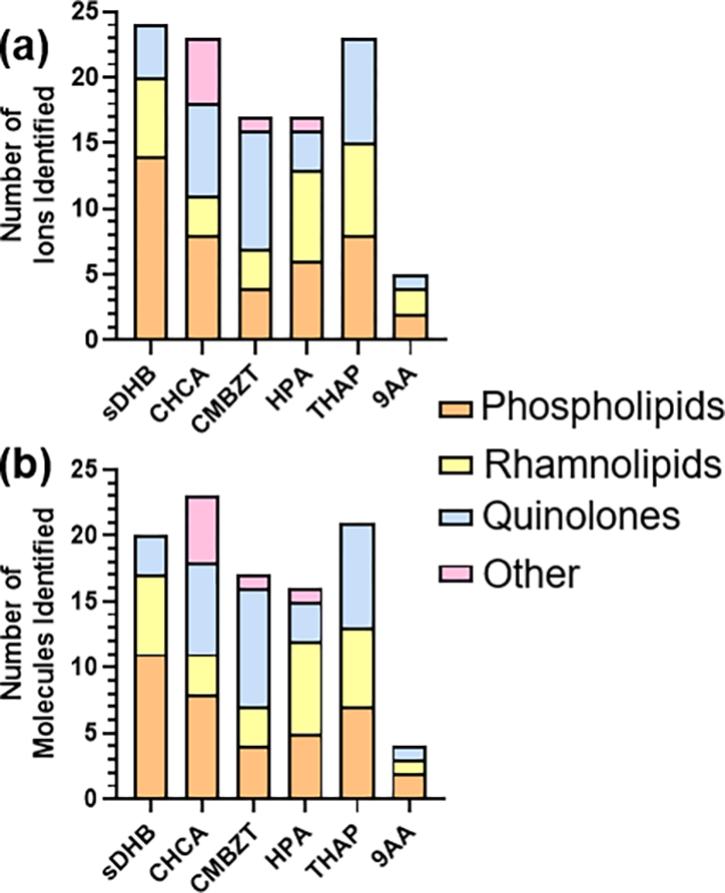
**Nominal ion identification.** (a) Total number of each identified ion including all precursor ion adducts in the quinolone, phospholipid, rhamnolipid, or other classes found within each matrix. (b) Total number of molecules identified for each class found in each of the matrices.

Though all of these 211 precursor ions were attempted to be fragmented by CID, only 109 ions were selected and fragmented (Figure 1a, Table S1). It was difficult to fragment precursor ions within the lower mass range (*m*/*z* < 350), despite including the sulfur calibrant. As such, many of the nominal ions of interest could not be properly identified. Only fragmented ions that were identified were included in our statistical analysis. In some cases, ions were identified as different sodiated adducts of the same molecule (Figure 1a, Tables S6 and S7). Taking this into consideration, ions identified as the same molecule within a matrix were counted only once for molecule abundance analysis and data collection. In total, 101 molecules were identified across the six matrices tested (Figure 1b, Tables S6 and S7).

### Total Abundances of Observed Ions across Matrices

**Figure 2:**
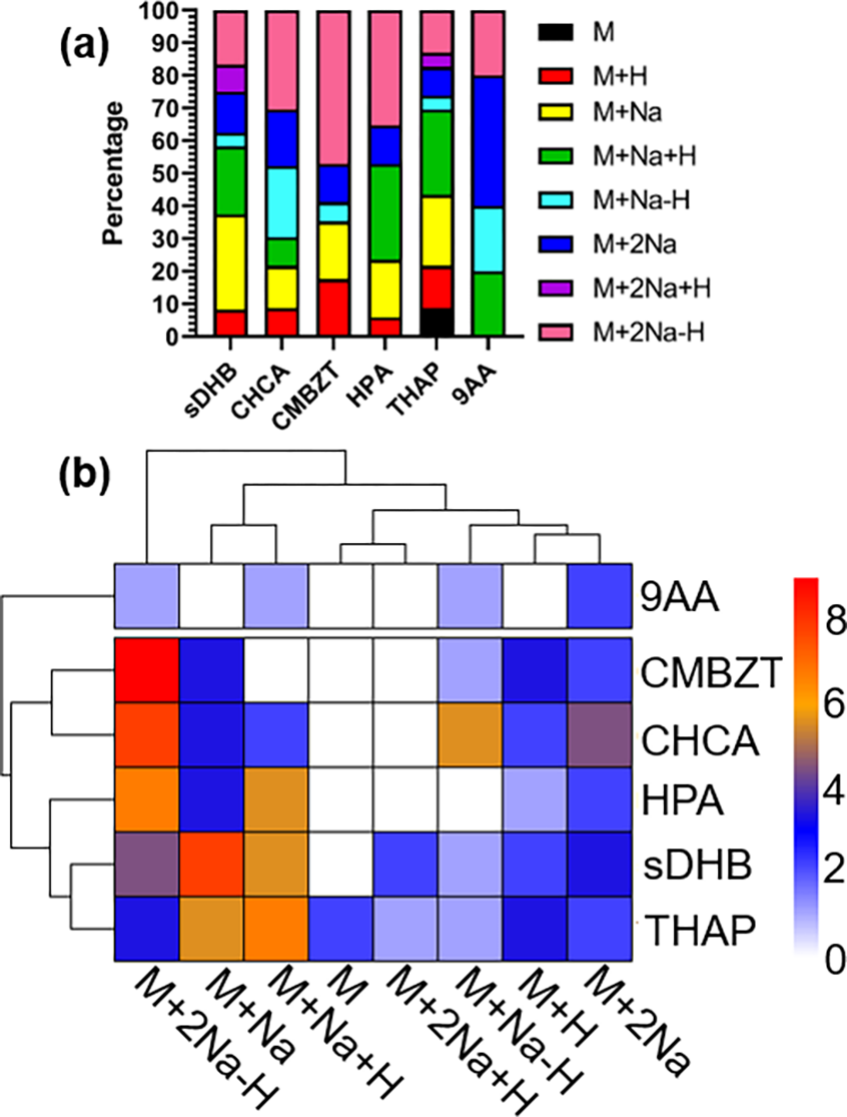
**Adduct categories.** (a) Observed distribution of adducts of identified ions. Percentages of the number of each adduct occurring within each matrix. (b) Hierarchical clustering constructed in R version 4.1 using the pheatmap (version 1.0.12) R package.^51^ Clustering was based on the complete distance between the observed adduct types and the matrices. Scales are based on the total whole number value of each adduct identified within each of the matrices.

Following fragmentation and identification, a wide range of molecule types and adducts were observed. All matrices formed multiple adducts (Figure 2a, Table S6). The matrices THAP and sDHB when prepared in the less polar solvent system (ACN:water) shared similar adduct patterns, favoring formation of [M + Na]^+^ and [M + Na + H]^+^ adducts (Figure 2b). The matrices CHCA, HPA, and CMBZT when prepared in the more polar solvent systems (water or methanol:water) favored [M + 2Na – H]^+^ adducts (Figure 2b), with 47% of the observed adducts in CMBZT being [M + 2Na – H]^+^ adduct ions (Figure 2a). Previously, it has been reported that the matrix solvents can play a role in ionization efficiency.^50^ Our results suggest that increased polarity of the matrix solvent system could result in an increase in the appearance of sodium adducts independent of salt concentration. Residual water entrapped within the crystals in dried droplet sampling^50^ could also contain more sodium ions within the crystals and allow for more MALDI ion sodium adducts. These trends are important to note when examining the appearance of precursor ions in mass spectra for further CID isolation and identification.

Three major classes of molecules were observed in this work. Quinolones, phospholipids, and rhamnolipids were identified across all six matrices. These surface associated molecules are known to play roles in signaling and structural composition of *P. aeruginosa*.^12,52^ Some amino acids, lactones, and other small biomolecules were also identified, but due to low ionization in the lower *m*/*z* range, these were not commonly observed across all matrices. The identified ions (Figure 1a) and molecules (Figure 1b) revealed the selectivity of each matrix. More molecules were identified by CHCA, THAP, and sDHB, when comparing overall molecule totals (Figure 1b). However, each matrix varied in its ionization efficiency from the ions of interest (Table S1). The percent identification for each matrix differed and was defined by the number of ions identified by CID to the initial number of ions shown by MALDI-TOF (Table S1). Using CHCA, most (92%) of the originally observed ions were identified by CID. Following this was THAP with 68%, HPA with 49%, CMBZT with 52%, sDHB with 44%, and 9AA with only 17% identification (Table S1).

Each matrix revealed varying distributions of molecule classes (Figure 1). A total of 24 ions were identified with sDHB, and 20 of those were different molecules. Of those, 4 ions were quinolones (3 molecules), 14 were phospholipid ions (11 molecules), and 6 were rhamnolipid molecules. The matrix CHCA revealed 23 molecules, including 7 quinolones, 8 phospholipids, and 3 rhamnolipids. Using CHCA, 5 other molecules were identified, including phosphoenolpyruvate, *N*-(3-hydroxybutanoyl)-l-homoserine lactone (3-OH-C_4_-AHL), arginine, kynurenine, and arogenic acid (pretyrosine) which did not fit under the three major classes observed. A total of 23 ions were discovered using THAP which presented 21 different molecules. Of the 23 observed ions, 8 were quinolone molecules, 8 were phospholipid ions (7 molecules), and 7 were rhamnolipid ions (6 molecules). The matrix CMBZT generated 17 ions, including 9 quinolone molecules, 4 phospholipid molecules, 3 rhamnolipid molecules, and 1 lactone. A total of 17 ions were identified using HPA with 16 being different molecules. These ions consisted of 3 quinolone molecules, 6 phospholipid ions (5 molecules), and 7 rhamnolipid molecules. The one additional molecule identified was palmitic acid. The final matrix, 9AA, only revealed 5 ions, which consisted of 2 phospholipids, 1 quinolone, and 2 rhamnolipids (1 molecule). The remainder of the ions for 9AA were not able to be identified and or selected for fragmentation analysis. This is likely due to this matrix accepting labile protons, leading to more negatively charged ion adducts not applicable to this analysis.^35^

**Figure 3:**
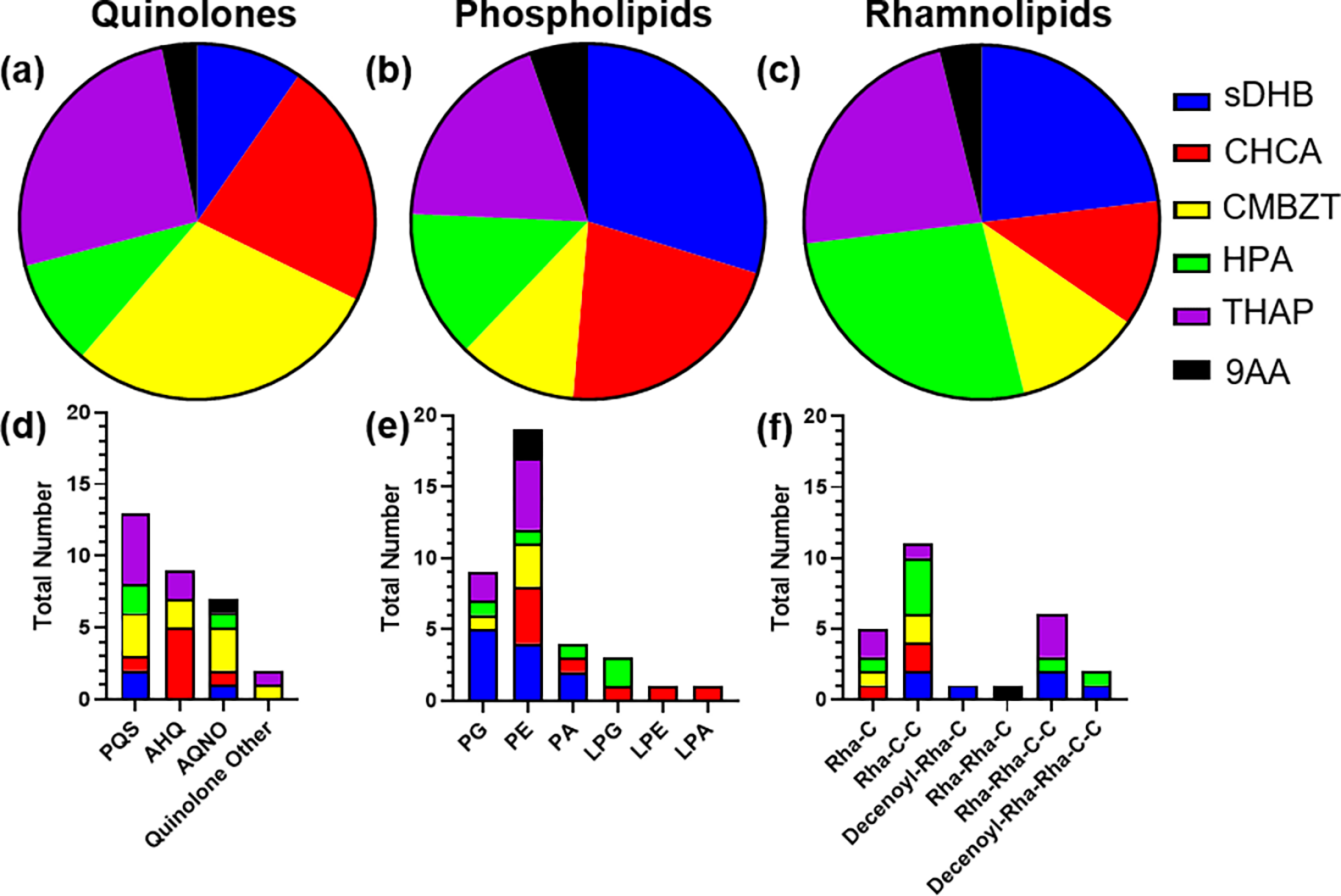
**Major molecule class examination.** Pie charts depicting the proportion of the total amount for (a) quinolone, (b) phospholipid, and (c) rhamnolipid class molecules found with each matrix. Distribution of total number of identified molecules from each of their subclasses of (d) quinolones, (e) phospholipids, and (f) rhamnolipids.

The following distribution of each major class was observed between matrices. A total of 31 quinolone molecules were identified with 9 (29%) occurring in CMBZT, 8 (26%) in THAP, 7 (23%) in CHCA, 3 (10%) in both sDHB and HPA, and 1 (3%) in 9AA (Figure 3a). A total of 37 phospholipid molecules were identified with 11 (30%) in sDHB, 8 (22%) in CHCA, 7 (19%) in THAP, 5 (14%) in HPA, 4 (11%) in CMBZT, and 2 (5%) in 9AA (Figure 3b). Finally, a total of 26 rhamnolipid molecules were identified with 7 (27%) being observed in HPA, 6 (23%) in both sDHB and THAP, 3 (12%) in both CHCA and CMBZT, and 1 (4%) in 9AA (Figure 3c). Various molecule subclasses of each major class were determined across the matrices (Figure 3d–f).

The ions identified by CID fragmentation were different between each of the matrices (Tables S6 and S7). Of all matrices used, CHCA had the widest range of ions identified, *m*/*z* 190–813 (Table S1). The matrix HPA covered a range of *m*/*z* 297–848, THAP covered a range of *m*/*z* 257–827, CMBZT covered a range of *m*/*z* 260–783, 9AA covered a range of *m*/*z* 372–762, and sDHB covered a range of *m*/*z* 310–827 (Table S1). The use of uncomplicated matrix preparation methods allowed for the identification of a wide range of ions identified as *P. aeruginosa* cell associated molecules.

### Identification of Quinolones

**Figure 4:**
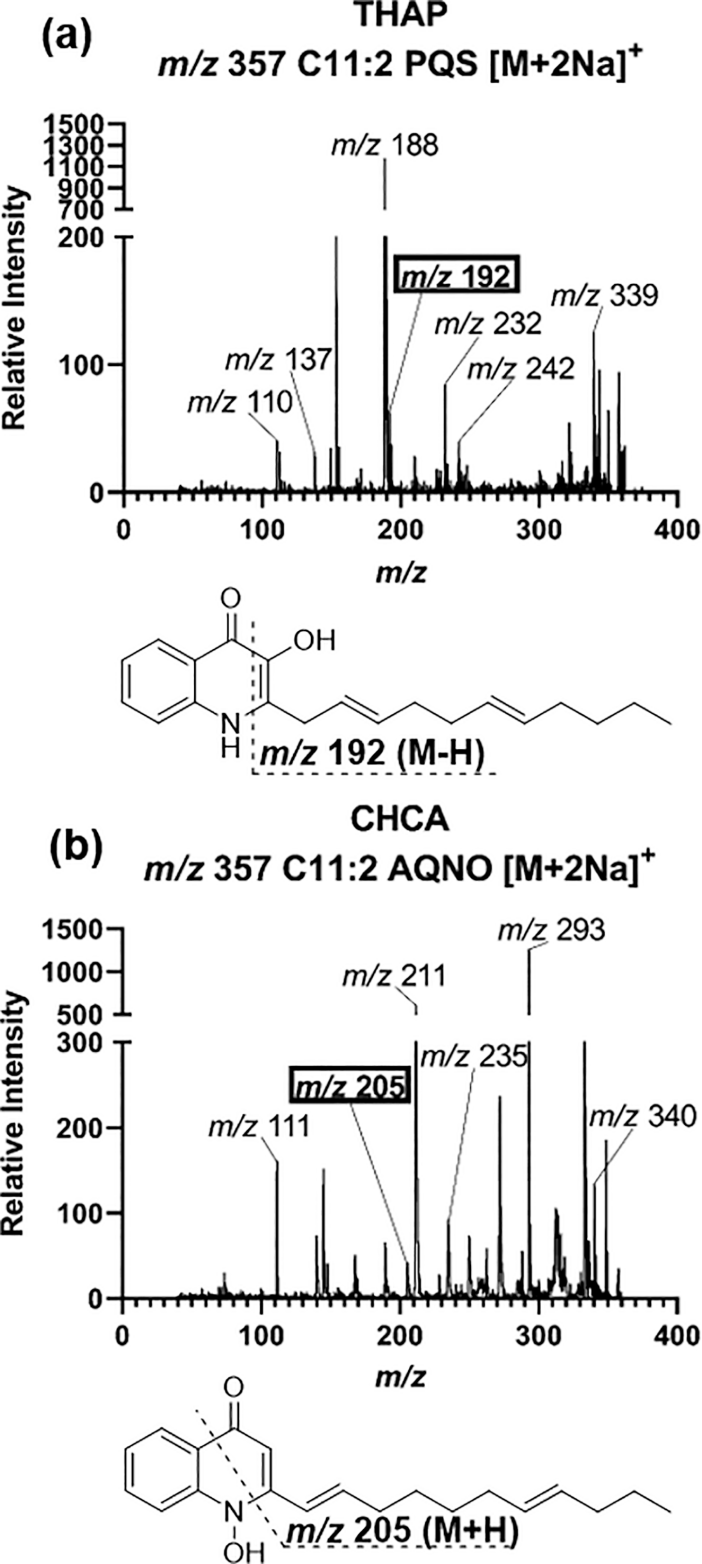
**Identifying ring break fragmentation for *m***/***z* 357.** (a) CID spectrum using THAP matrix identified as C11:2 PQS [M + 2Na]^+^ with *m*/*z* 192 [M – H]^+^ highlighted as the identifying ring break fragment. (b) CID spectrum using CHCA identified as C11:2 AQNO [M + 2Na]^+^ with *m*/*z* 205 [M + H]^+^ highlighted as the identifying ring break fragment.

Three major types of quinolones are observed in *P. aeruginosa*; these are classified as PQS, AQNO, and AHQ. Because the quinolone rings are often isomeric or isobaric, the rings must be fragmented to distinguish between PQS, AQNO, and AHQ molecules. Quinolone standards were used to confirm the ring break fragmentation patterns (Figure S3, Table S3). All standards were analyzed in reflectron positive scans to confirm their presence in each matrix spot. However, it was difficult to ionize certain quinolone standards with some matrices. The matrices HPA and THAP failed to ionize the AHQ standard, HHQ, and were only able to select and show a single fragment for the AQNO standard (Table S3). It was difficult to ionize the PQS and HHQ standards with sDHB, and each observed only two fragment ions after CID (Table S3). The matrices CHCA and CMBZT were able to ionize and adequately fragment all quinolone standards tested (Table S3). When utilizing cellular samples, THAP failed to ionize AQNO quinolones, and similarly, sDHB and HPA both failed to ionize any AHQ quinolones (Figure 3d, Table S6). The classes of standard molecules that were not able to ionize with specific matrices were unlikely to ionize in the cell samples. Furthermore, several unidentified ions of interest such as *m*/*z* 244.13 in sDHB and *m*/*z* 260.15 in HPA have nominal masses, which corresponded to the AHQ and AQNO standards respectively (Table S1). Neither of these were properly identified by CID fragmentation despite appearing as ion peaks of interest in initial scans. Similarly, when analyzing the standards, ion peaks corresponding to AHQ and AQNO appeared in initial scans; however, the instrument did not select the ions for CID fragmentation. Despite this, several quinolone rings were fragmented across the matrices to enable determination of specific quinolone subclasses (Table S7). In some cases, the identification of a nominal ion changed from one class of quinolone to another depending on the matrix used. The *m*/*z* 357 was a specific example (Figure 4). In THAP, the ion fragment *m*/*z* 192 [M – H]^+^ corresponded with the PQS quinolone ring, allowing the *m*/*z* 357 to be identified as C11:2 PQS (Figure 4a). However, at the same precursor ion mass in CHCA, the fragment *m*/*z* 205 [M + H]^+^ corresponded as an AQNO quinolone ring and was therefore identified as C11:2 AQNO (Figure 4b).

Analysis by CID fragmentation displayed selectivity of quinolone derivatives between the matrices. The ions *m*/*z* 338 and *m/z* 339 were identified as C11:PQS with the adduct forms of [M + Na]^+^ and [M + Na + H]^+^ respectively (Figure S4). This molecule was observed across all matrices except 9AA. For the remainder of observed quinolones, THAP favored ionization of PQS ions. The nominal ion *m*/*z* 357 (Figure 4a) was identified as a PQS molecule, while CHCA showed the ions as AQNO derivatives (Figure 4b). The matrices HPA and sDHB ionized predominantly PQS molecules over other quinolones. The matrix CHCA primarily ionized AHQ molecules. Compared to the other matrices, CMBZT ionized a variety of quinolone classes and showed no preferential ionization (Figure 3d). The nominal ion *m*/*z* 304 was identified as three quinolone molecules in CMBZT (Figure S5).

### Identification of Phospholipids

There are a variety of phospholipids found on the surface of *P. aeruginosa*.^8^ In this work, 39% (42 out of 109) of the total ions were identified as phospholipids, which were classified into six subclasses. Typically, sDHB has been used to analyze phospholipids with MALDI-TOF.^42^ However, the charges of different phospholipid headgroups may play a role in ionization selectivity between matrices.^18^ Characteristic headgroup fragments were used to identify phospholipid subclasses (Table S4). Often phospholipids fragmented twice at both the headgroup and in the alkyl chain (Figure S6, Table S4). These fragmentation patterns were similarly observed in the phospholipid standards (Table S4).

**Figure 5:**
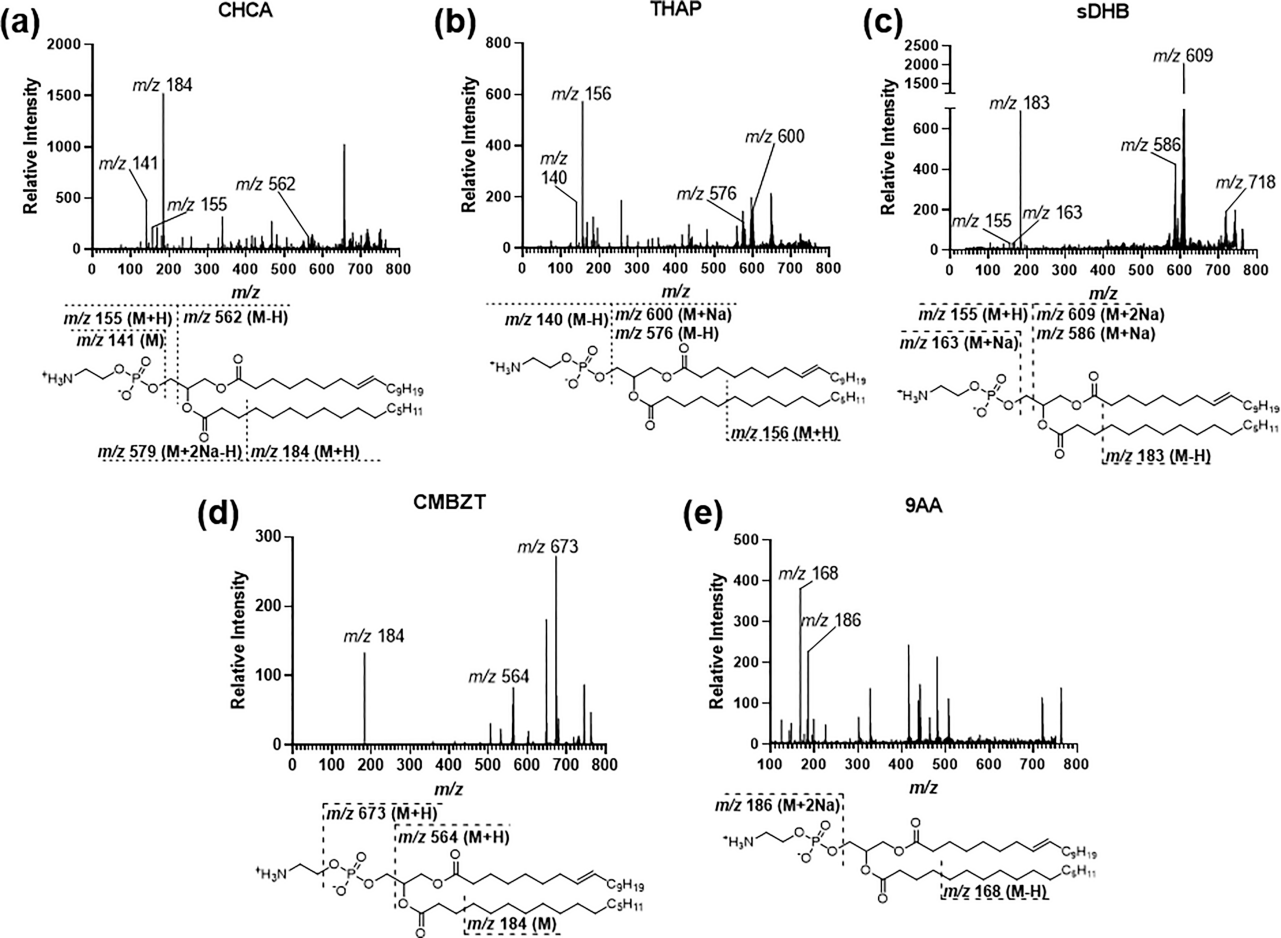
**Mass spectral comparison of *m***/***z* 763 PE (18:1/16:0) [M + 2Na]^+^**. (a–e) CID spectra for the *m*/*z* 763 ion. Labeled fragment peaks are depicted below each spectrum.

Phosphatidylethanolamine (PE) derivatives are common molecules in the membrane of *P. aeruginosa* and other Gram-negative bacteria.^11,37^ In this study, 51% (19 out of 37) of the identified phospholipids across the 6 matrices were PE phospholipids. The percentages of PEs identified compared to the total phospholipid molecules differed within each matrix: sDHB 36% (4 out of 11), CHCA 50% (4 out of 8), CMBZT 75% (3 out of 4), HPA 20% (1 out of 5), THAP 71% (5 out of 7), and 9AA 100% (2 out of 2). The phospholipid PE (18:1/16:0) was identified in all matrices except HPA. This specific phospholipid was observed as multiple adducts in both THAP and sDHB (Table S6). The most common adduct observed by PE (18:1/16:0) was [M + 2Na]^+^ (Figure 5). Phosphatidic acids (PAs) were only detected in sDHB, CHCA, and HPA samples. Phosphatidylglycerols (PGs) were observed in all matrices expect CHCA and 9AA. Of the seven different PG phospholipid molecules observed, sDHB was able to ionize five of the eight PG phospholipids (Table S6).

Lysophospholipids come from degradation of membranes or as intermediates of other phospholipids.^11^ The abundance of specific lysophospholipids in bacteria can increase in response to stress and alter membrane integrity.^53,54^ Lysophosphatidylglycerols (LPGs) were ionized by both CHCA and HPA, while derivatives of lysophosphatidic acids (LPAs) and lysophosphatidylethanolamines (LPEs) were observed only by CHCA (Figure 3e, Table S6).

Lipid extraction and additives have been previously utilized with the intent to improve ionization of phospholipids.^36,55^ Our study did not extract phospholipids or use additives to assist ionization. Despite forgoing these preparations, multiple variations of phospholipids were identified including PA, PE, PG, and lysophospholipids. Some phospholipid molecules were not observed within our analysis including phosphatidylserines (PSs), cardiolipins (CLs), and phosphatidylcholines (PCs). Ionization of PS is likely limited in positive ion mode due to the negative charge on the headgroup.^18^ The CLs have higher molecular weights, which were not observed in our experiments.^47^ The headgroup quarternary ammonium of the PC phospholipids have a permanent positive charge. However, no PC phospholipids were identified despite this charge favoring positive ion mode analysis.^18^ The PC phospholipids are known to be present in the outer membrane of *P. aeruginosa* strains,^56^ but the overall abundance were lower than other phospholipids.^45^ The low abundance and the complexity of the sample could explain the lack of detection in this study without lipid extractions. As such, alternative sample preparation methods or other matrices may be required for the ionization of PC molecules in bacterial cells.

### Identification of Rhamnolipids

**Figure 6:**
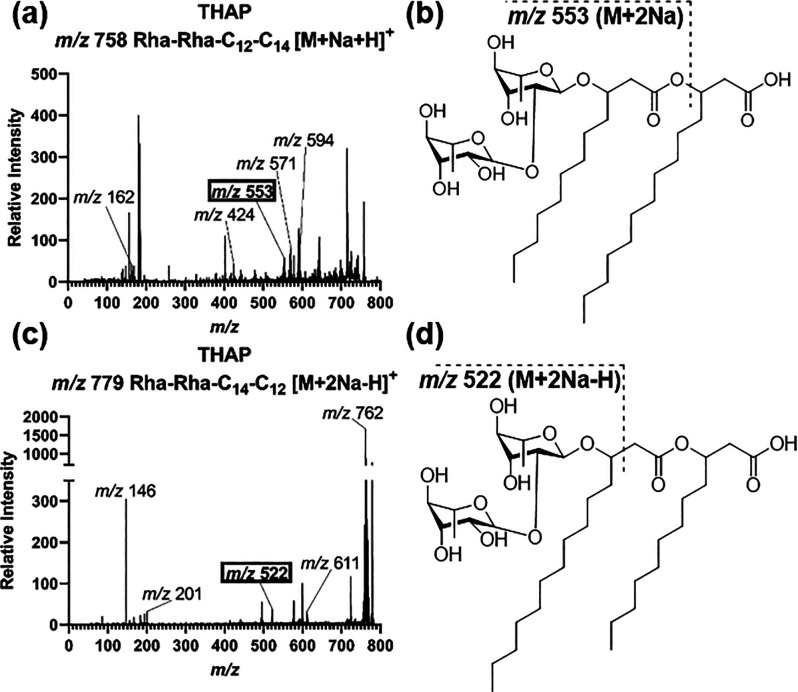
**Spectral comparison of di–rhamnolipid C**_**12**_**–C**_**14**_. (a) CID spectra for the *m*/*z* 758 ion in THAP, which was identified as Rha–Rha–C_12_–C_14_. Highlighted fragment of *m*/*z* 553 was key in identification. (b) *m*/*z* 553 [M + 2Na]^+^ occurrence in the Rha–Rha–C_12_–C_14_. (c) CID spectra for the *m*/*z* 779 ion in THAP, which was identified as Rha–Rha–C_14_–C_12_. Highlighted fragment of *m*/*z* 522 was key in identification. (d) *m*/*z* 522 [M + 2Na]^+^ occurrence in the Rha–Rha–C_14_–C_12_.

Although *P. aeruginosa* produces rhamnolipids, their role in bacterial cultures is still being elucidated. Rhamnolipids can alter microbial surfaces for increased uptake of hydrophobic substrates^17^ as well as play roles in motility and biofilm development.^57^ In total, 28 rhamnolipid ions (26 molecules) were observed in this work. This number accounted for roughly 25% of the total number of ions identified in this study. Characteristic fragmentation patterns, determined from commercial standards, were typically between the rhamnose headgroup and the alkyl chains (Table S5). However, fragmenting between the rhamnose headgroup alone was not sufficient for identification. If the rhamnose headgroup (*m*/*z* 147) was sodiated (*m*/*z* 170), it matches the fragment ion (M) of a PG headgroup (Figure S7). Thus, further analysis of other fragment ions was necessary for identification. In both cells and standards, fragments were occasionally observed at the ester bond between the two aliphatic chains, which provided evidence of the chain lengths (Tables S5 and S7). Mono–rhamnolipid (Rha) molecules accounted for 61% (16 out of 26) of observed rhamnolipid associated molecules, with 69% (11 out of 16) of those containing two aliphatic chains (Rha–C–C). All matrices (except 9AA) ionized Rha–C, Rha–C–C, and Rha–Rha–C–C rhamnolipid classes. The only rhamnolipid identified in 9AA was Rha–Rha–C_8_, which ionized with two different adducts (Tables S6 and S7). Both sDHB and HPA included the identification of rhamnolipids containing an α-decenoyl moiety at the 2-hydroxyl group position of the rhamnose, as seen previously.^17^ Also of interest, fragmentation patterns and adducts in THAP led to the differential identification of both Rha–Rha–C_12_–C_14_ (*m*/*z* 758 [M + Na + H]^+^) and Rha–Rha–C_14_–C_12_ (*m*/*z* 779 [M + 2Na – H]^+^) (Figure 6). The THAP matrix was able to differentiate between two rhamnolipid isomers. Most matrices allowed for the identification of a variety of rhamnolipids.

### Matrix Dependent Identification of Ions

**Figure 7:**
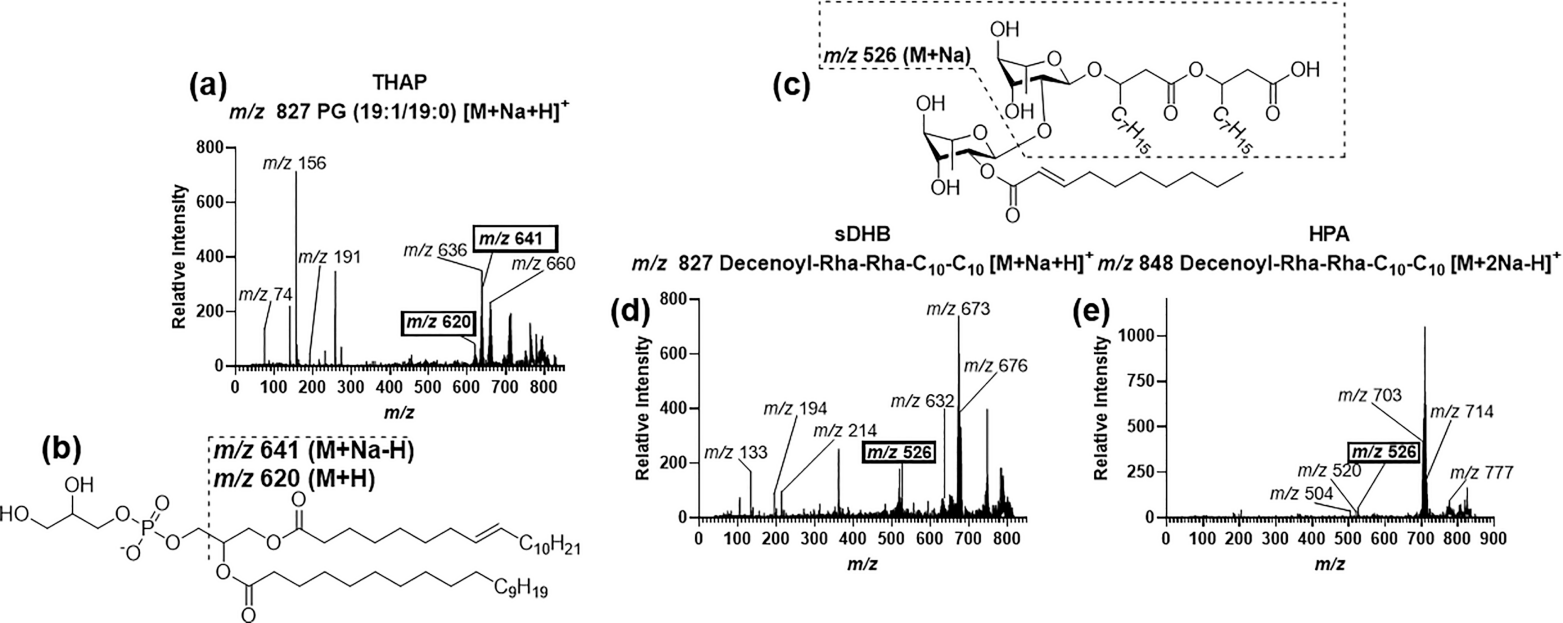
**Differences in matrix fragmentation for *m***/***z* 827 precursor ion.** (a) CID spectrum of ion *m*/*z* 827 with THAP matrix, identified as PG(19:1/19:0) [M + Na + H]^+^. Labeled peaks are unique and were used to identify the molecule. (b) Structure of PG (19:1/19:0) with identifying fragments depicted. (c) Structure of decanoyl–Rha–Rha–C_10_–C_10_ with the key *m*/*z* 526 [M + Na]^+^ fragment noted. (d) CID spectrum for *m*/*z* 827 with matrix sDHB identified, as decanoyl–Rha–Rha–C_10_–C_10_ [M + Na + H]^+^. Ion identified as a decanoyl–Rha–Rha–C_10_–C_10_ based upon identified fragment ions. (e) CID spectrum for *m*/*z* 848 in HPA, which was identified as decanoyl–Rha–Rha–C_10_–C_10_ [M + 2Na – H]^+^.

Several matching nominal ions were identified as nonidentical molecule classes following CID analysis with different matrices. We compiled these examples to demonstrate matrix dependent molecular ionization of specific molecule classes. As shown in Figure 4, different matrices ionized different quinolones with the same *m*/*z*. Other dissimilar lipid classes also ionized at the same *m*/*z* using different matrices. An interesting instance of this is *m*/*z* 827 (Figure 7a–d). Upon analysis in THAP, fragmentation determined ion *m*/*z* 827 to be a PG (19:1/19:0) [M + Na+H]^+^ (Figure 7a,b). However, in sDHB, *m*/*z* 827 was identified as decenoyl–Rha–Rha–C_10_–C_10_ [M + Na + H]^+^ (Figure 7c,d). The key difference between the two CID spectra was the presence of ions in the *m*/*z* 500–600 range in sDHB. The fragment ion, *m*/*z* 526 [M + Na]^+^, demonstrates fragmentation of the α-decenoyl and rhamnose group (Figure 7c). The decenoyl–Rha–Rha–C_10_–C_10_ standard in sDHB contained an *m*/*z* 525 [M + Na – H]^+^ ion fragment, which corresponded to the same break showing the loss of the α-decenoyl and rhamnose group (Table S5). This *m*/*z* 526 ion functioned as a characteristic fragment for the identification of the decenoyl–Rha–Rha–C_10_–C_10_ molecule in sDHB. This decenoyl–rhamnose fragment was similarly found in fragmentation of ion *m*/*z* 848 in HPA, which was identified as decenoyl–Rha–Rha–C_10_–C_10_ [M + 2Na – H]^+^ (Figure 7c,e). The fragment ion *m*/*z* 526 was absent from the THAP spectrum, showing that it was not a rhamnolipid. Based on the presence of the fragment ions *m*/*z* 641 [M + Na – H]^+^ and *m*/*z* 620 [M + H]^+^, the ion *m*/*z* 827 in THAP was identified as a phospholipid (Figure 7a,b).

Other examples of matrix dependent nominal ion identifications were observed. The ion *m*/*z* 657 in CHCA was identified as PE (14:1/14:0) [M + Na + H]^+^ (Figure S8a,b) but was identified as decenoyl–Rha–C_10_–C_10_ [M + H]^+^ in sDHB (Figure S8c,d). Similarly, the *m*/*z* 779 ion was identified as PE (18:0/17:0) [M + 2Na]^+^ (Figure S9a,b) in CHCA but was identified as Rha–Rha–C_14_–C_12_ [M + 2Na – H]^+^ in THAP (Figure S9c,d). Based on these differences, despite preference for phospholipid ionization (Figure 1a), sDHB showed ionization of decenoyl–rhamnolipids over a PG and PE phospholipid (Figure 7 and Figure S8). The matrix CHCA favored ionization of PE phospholipids and demonstrated low ionization of rhamnolipids (Figures 1a and 3e). Fragmentation of the same nominal ion from THAP showed no preferential ionization toward phospholipids or rhamnolipids. Overall, the results collected here provide further support of matrix specificity for ionization of different molecule classes.

### Suitable Matrix Selection

For each quinolone molecule subclass, an optimal matrix was identified. The identification of each quinolone ion adduct and matrix is represented in Table S6. Quinolone analysis found that matrix selection influences the class of quinolones observed. The most quinolones were ionized in CMBZT, which accounted for 29% (9 out of 31) of all quinolone molecules identified (Figure 3a). The matrix CMBZT ionized three different quinolones at the nominal ion of *m*/*z* 304 (Figure S5). The matrix CMBZT ionized the largest variety of quinolone derivatives. Most identified quinolone molecules in THAP, 63% (5 out of 8) were PQS, and none were AQNO (Figure 3d). This finding was supported by difficulty with the ionization and fragmentation of an AQNO standard when using THAP (Table S3). A total of 7 quinolone molecules ionized with CHCA, with 71% (5 out of 7) were identified as AHQs with the other molecules being PQS and AQNO derivatives (Figure 3d). The type of quinolone class should be considered before deciding which matrix should be used for analysis. For general analysis of quinolones, CMBZT appeared to be the optimal matrix. For analysis of the PQS quinolone subclass, THAP was the best choice of matrix. Finally, for the AHQ quinolone subclass, the best preference for matrix was CHCA.

Specific subclasses of phospholipids were ionized depending on the matrix used (Figure 3e, Table S6). Phospholipids, PG and PA, were ionized with sDHB (Figure 3e). However, sDHB did not ionize lysophospholipids. Lysophospholipids only ionized with CHCA and HPA. More unique lysophospholipid molecules were identified with CHCA (3 out of 5) (Figure 3e). The PE subclass ionized well in sDHB, THAP, and CHCA. The subclasses of phospholipids ionized were dependent on the matrix.

Rhamnolipids generally ionized across multiple matrices (Figure 3f, Table S6). The matrix HPA can be used to ionize mono–rhamnolipids, di–rhamnolipids, and decenoyl–rhamnolipids (Figure 3f). Both the mono–rhamnose and di–rhamnose forms of the decanoyl–rhamnolipids were observed with sDHB (Figure 3f). Ionization of a di–rhamnolipid with one alkyl chain was only seen with 9AA (Figure 3f). The optimal matrix for the majority of rhamnolipid molecules present was HPA.

A select few lactones and amino acids were identified (Table S6). However, due to their lower molecular weights, they were difficult to fragment for identification. Despite this, CHCA ionized five and CMBZT and HPA ionized one each (Figure 1). Overall, CHCA was better at ionizing low molecular weight ions (<*m*/*z* 350).

## Conclusion

The diversity of molecules associated with *P. aeruginosa* cultures complicates direct analysis of select molecules. Though this work is limited to *P. aeruginosa*, the preparation and matrix selectivity can be applied to other biological organisms. The matrices and whole cell samples were prepared in a simplistic manner and allowed small molecules to be analyzed. From this study, quinolones, phospholipids, rhamnolipids, lactones, and amino acids were identified across six matrices. Each of the tested matrices exhibited ionization specificity to select molecule classes. This work contributes to a better understanding of MALDI matrix ionization of small molecules.

## Supporting Information Available

Additional experimental details including fragmentation analysis and adduct formation (PDF)
