# Supplementary material for: Comparison of Small Biomolecule Ionization and Fragmentation in Pseudomonas aeruginosa Using Common MALDI Matrices
Source: J Am Soc Mass Spectrom. 2023 Jan 25;34(3):355–65. doi: 10.1021/jasms.2c00157 (PMC9983012; doi:10.1021/jasms.2c00157)
Supplement: Supplementary file 1 — js2c00157_si_001.pdf [file js2c00157_si_001.pdf]

Supporting Information

Comparison small biomolecule ionization and fragmentation in *Pseudomonas aeruginosa* using common MALDI matrices

Nathan C. Wamer[1‡], Chase N. Morse[1‡], Jennifer N. Gadient[2], Taylor A. Dodson[1], Eric A. Carlson[1], Erin G. Prestwich[1*]

[1]Department of Medicinal and Biological Chemistry, University of Toledo, Toledo, Ohio, 43606 USA

[2]The College of Natural Sciences and Mathematics, NSM Instrumentation Center, University of Toledo, Toledo, Ohio, 43606 USA

Address reprint requests to Erin. G. Prestwich, 2801 W. Bancroft St., Toledo, Ohio, 43606, USA

Phone#: (419)-530-1944, Email address: Erin.Prestwich@utoledo.edu

SI Contents:

Figure S1. - Wash vs no wash comparison S3

Figure S2. - Structures and spotted MALDI matrices S4

Figure S3. - Quinolone ring breaks........ S5

Figure S4. - Fragmentation of single sodium adduct C11: PQS........ S6

Figure S5. - Fragmentation of CMBZT *m/z* 304 ion........ S7

Figure S6. - Lipid standard *m/z* 718 $[M+H]^+$ double break fragmentation S8

Figure S7. - Major headgroup fragments........ S9

Figure S8. - Differing fragmentation of *m/z* 657 ion........ S10

Figure S9. - Differences in matrix fragmentation at *m/z* 779 ion S11

Table S1. - Initial nominal ions of interest scans........ S12

Table S2. - Commercial lactone standard spectra and fragments........ S13

Table S3. - Commercial quinolone standards spectra and fragments S14

Table S4. - Commercial phospholipid standards spectra and fragments S19

Table S5. - Commercial rhamnolipid standards spectra and fragments........ S26

Table S6. - Adducts of identified molecules........ S32

Table S7 - Identified ions of interest spectra and fragments........ S35

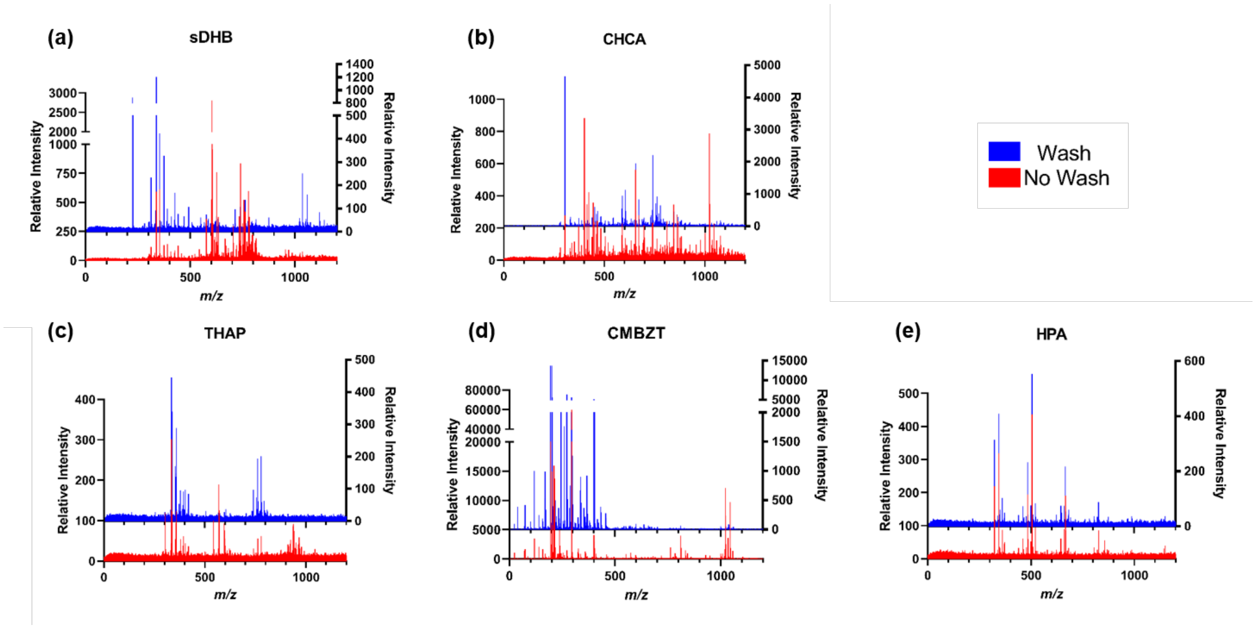

Figure S1. Wash vs no wash comparison. Comparison of reflectron positive ion scan mass spectrum between washing cells with 0.9% saline (blue) and not washing the cells (red) before spotting prepared cells in either **(a)** sDHB, **(b)** CHCA, **(c)** THAP, **(d)** CMBZT, or **(e)** HPA.

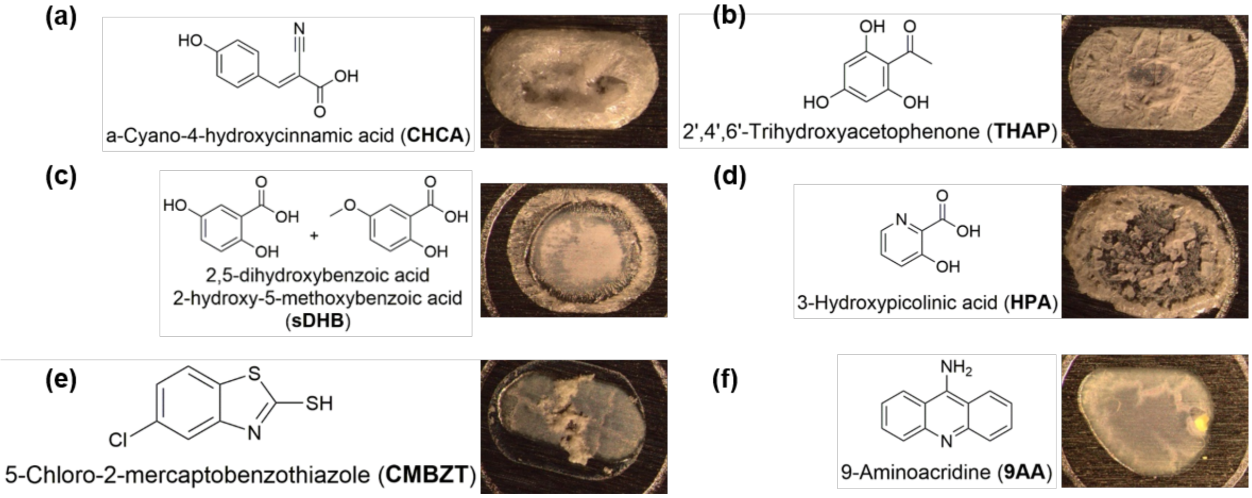

Figure S2. Structures and spotted MALDI matrices. **(a)** CHCA, **(b)** THAP, **(c)** sDHB, **(d)** HPA, **(e)** CMBZT, and **(f)** 9AA. All photos were taken under light microscope after spotting on a ground-steel plate prior to MALDI analysis.

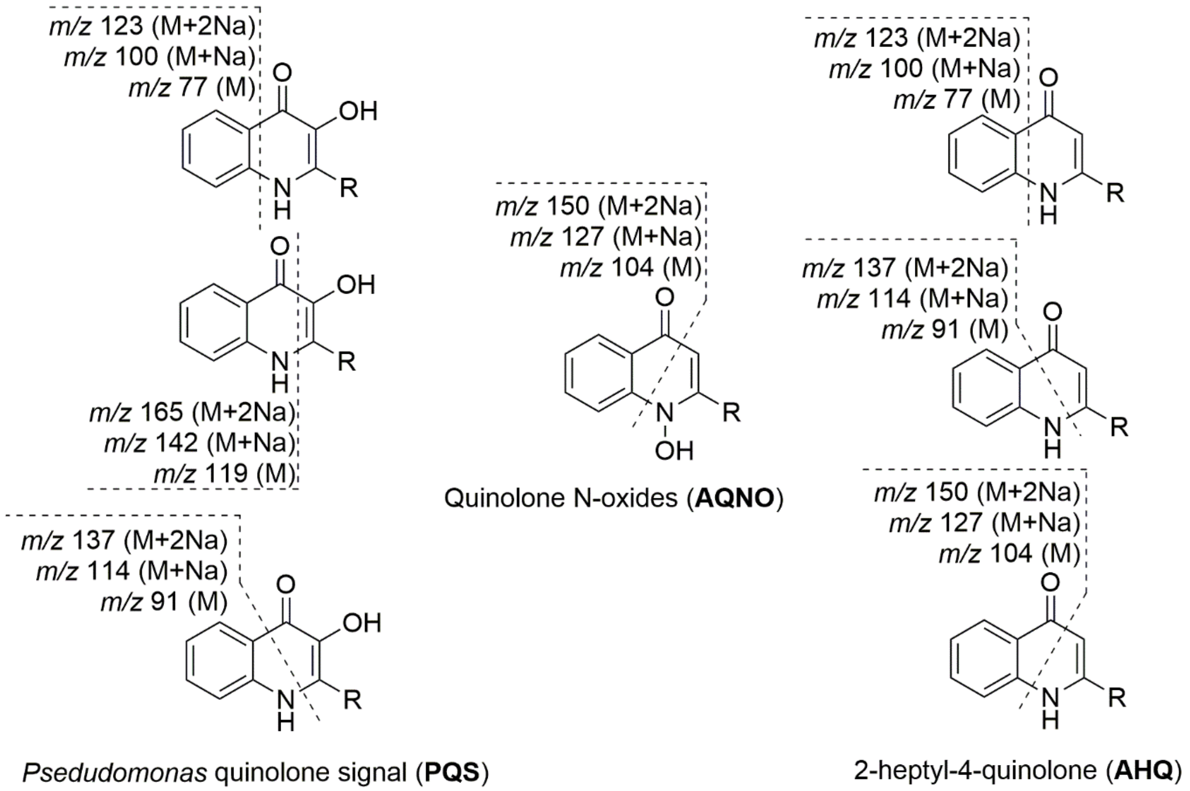

Figure S3. Quinolone ring breaks. Structures and fragment mass values for ring breaks observed in commercial quinolone standards. Singly and doubled sodiated adducts are also listed.

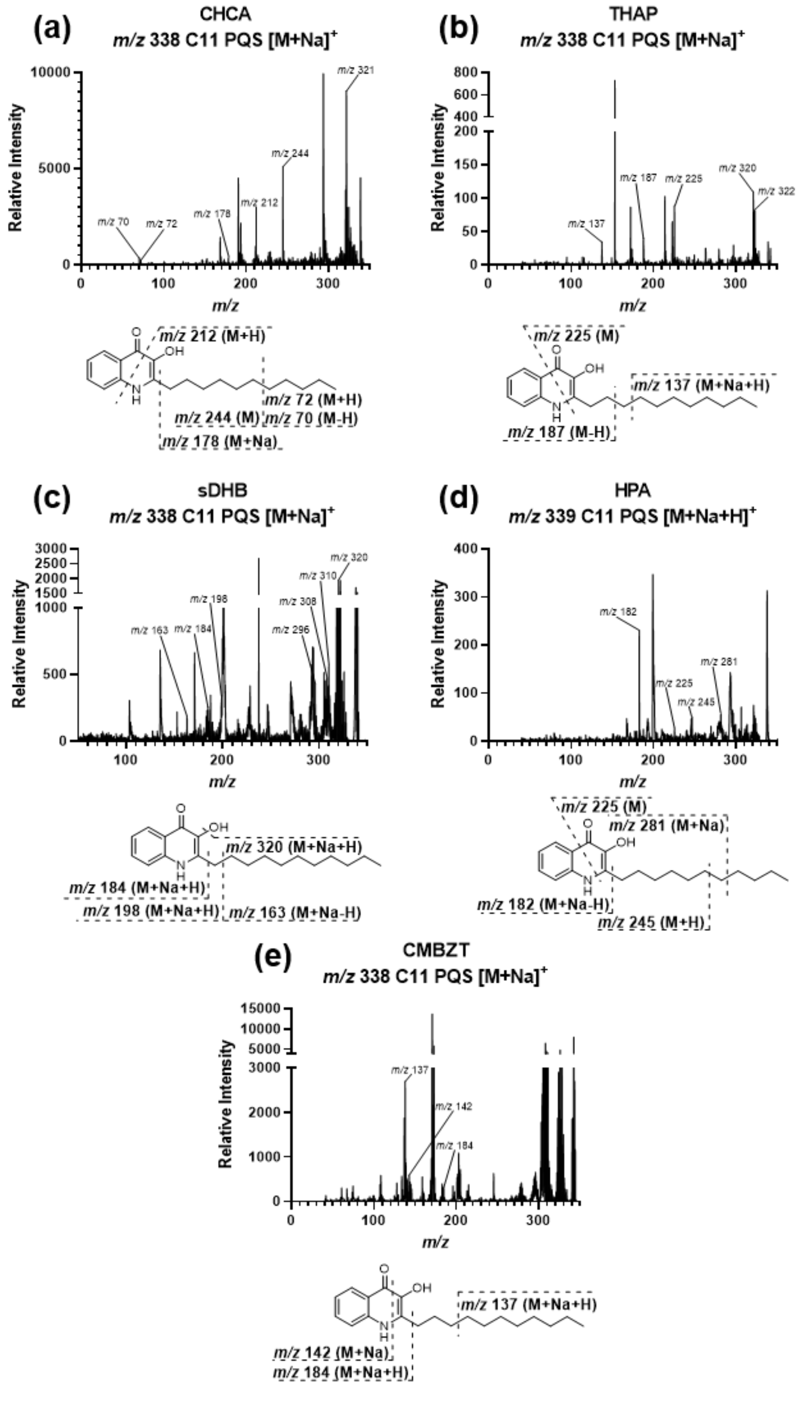

Figure S4. Fragmentation of single sodium adduct C11:PQS (a-e) CID spectra that were identified as C11:PQS from **(a)** CHCA, **(b)** THAP, **(c)** sDHB, **(d)** HPA, **(e)** CMBZT with the precursor adduct $[M+Na]^+$ or $[M+Na+H]^+$.

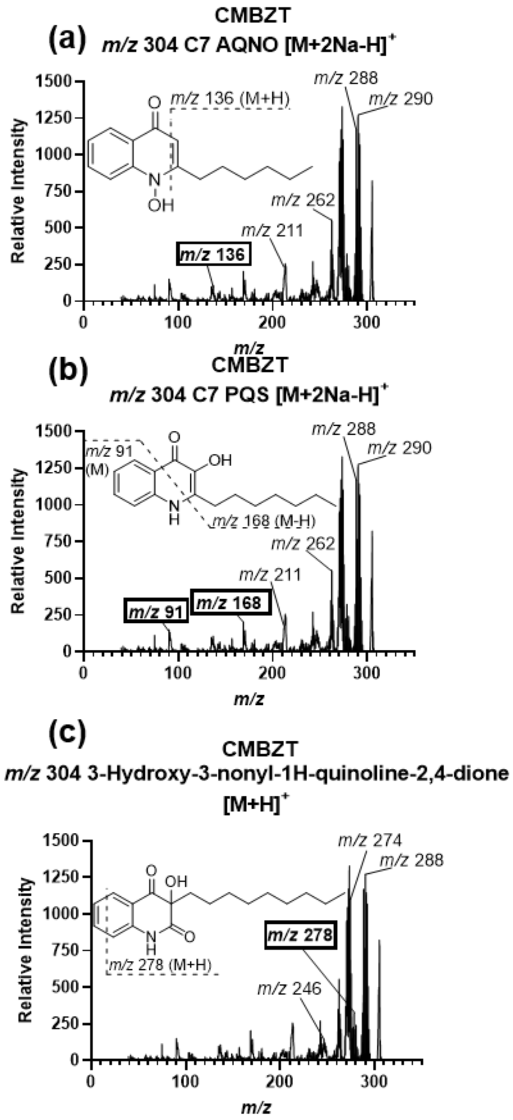

Figure S5. Fragmentation of CMBZT *m/z* 304 ion. (a) Collected CID spectrum identified as C7:AQNO $[M+Na-H]^+$. **(b)** Collected CID spectrum identified as C7:PQS $[M+2Na-H]^+$. **(c)** Collected CID spectrum identified as 3-Hydroxy-3-nonyl-1H-quinoline-2,4-dione $[M+H]^+$.

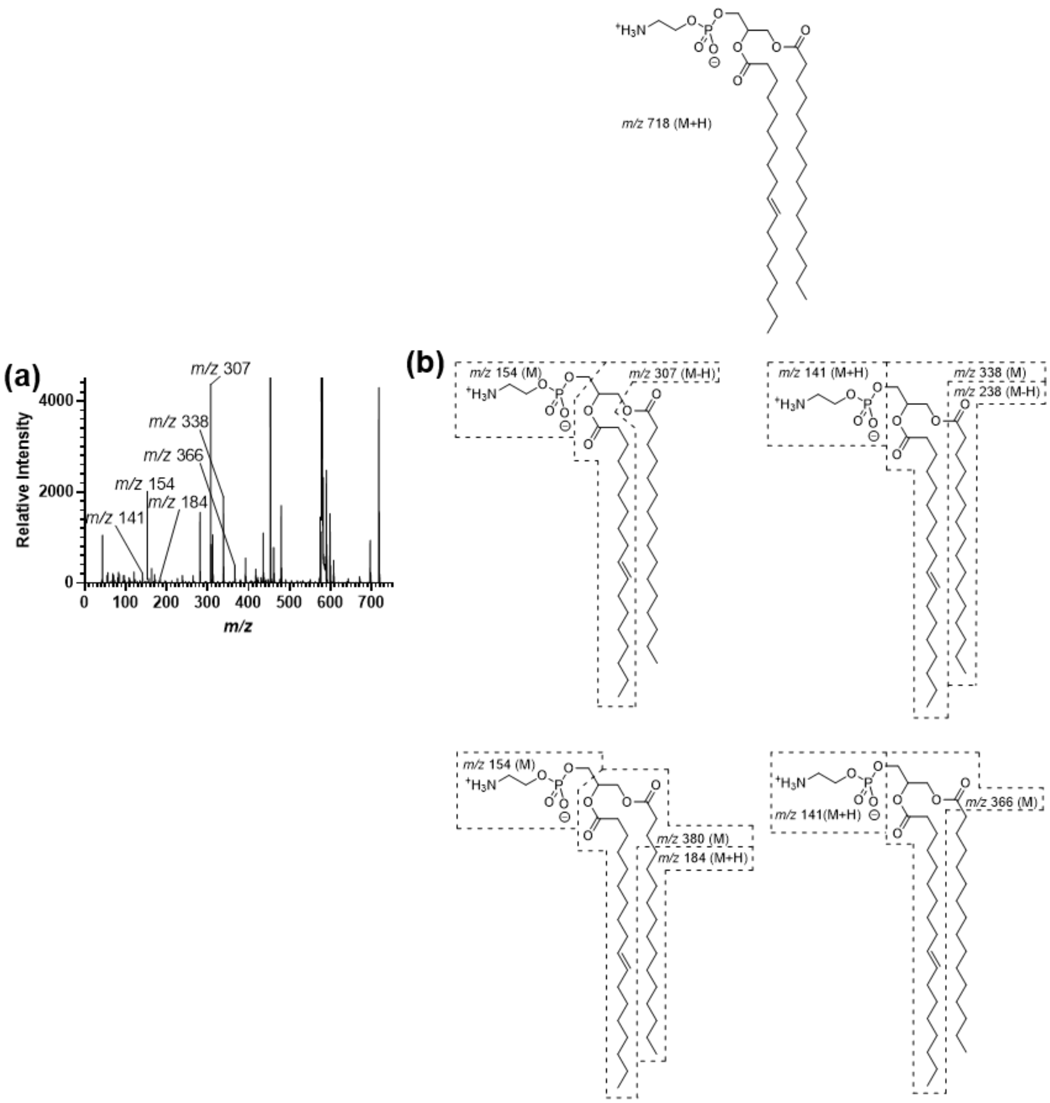

Figure S6. Lipid standard *m/z* 718 [M+H]⁺ double break fragmentation. (a) CID spectrum of lipid standard PE (18:1/16:0) [M+H]⁺ in CHCA. **(b)** Double break fragmentation of the PE (18:1/16:0) standard.

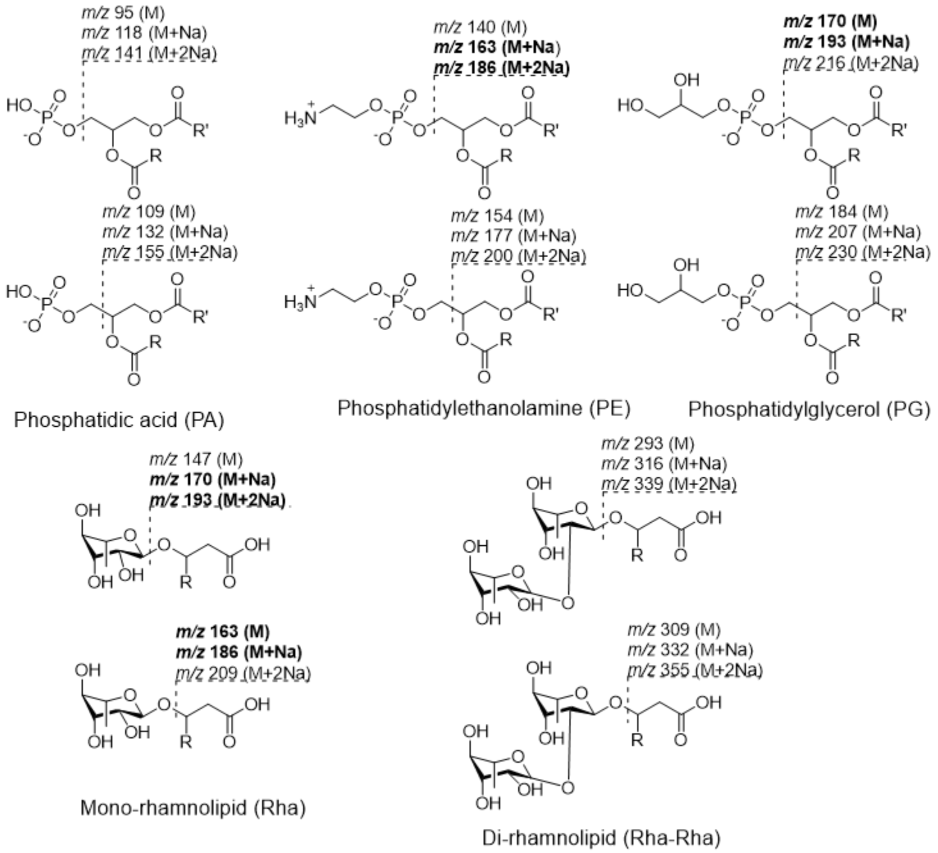

Figure S7. Major headgroup fragments. Structures of headgroup fragments possible for both phospholipids and rhamnolipids. The singly sodiated and doubly sodiated adduct values are listed. Highlighted fragments are those that are shared between two structures.

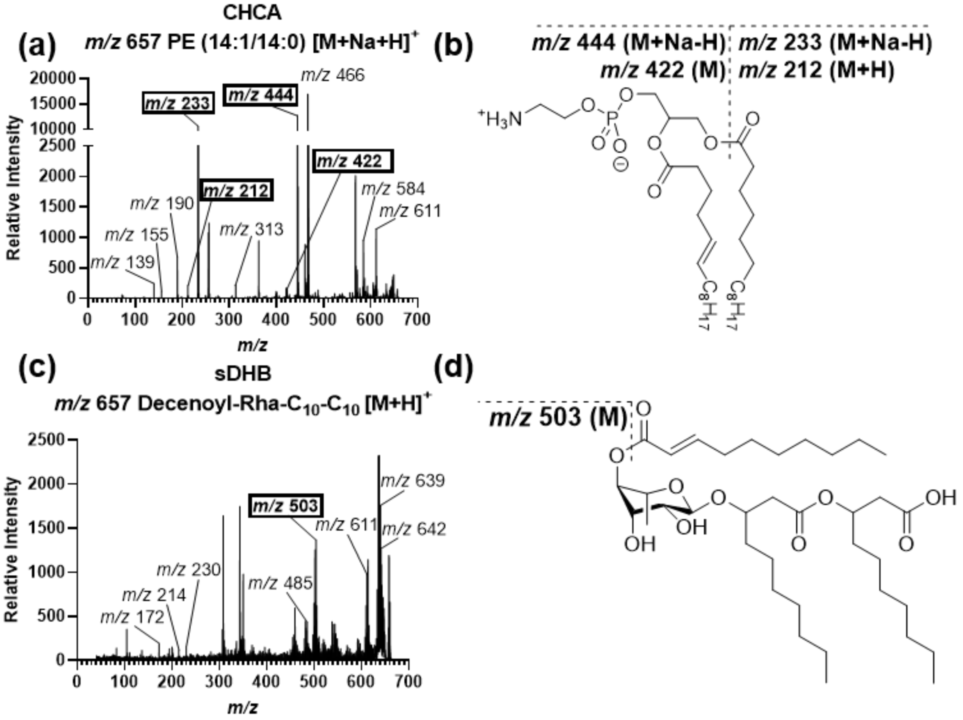

Figure S8. Differing fragmentation of *m/z* 657 ion. (a) Collected CID spectrum of *m/z* 657 in CHCA identified as PE (14:1/14:0) $[M+Na+H]^+$. **(b)** Structure of PE (14:1/14:0). **(c)** Collected CID spectrum of *m/z* 657 in sDHB identified as Decenoyl-Rha-C_{10}-C_{10} $[M+H]^+$. **(d)** Structure of Decenoyl-Rha-C_{10}-C_{10}.

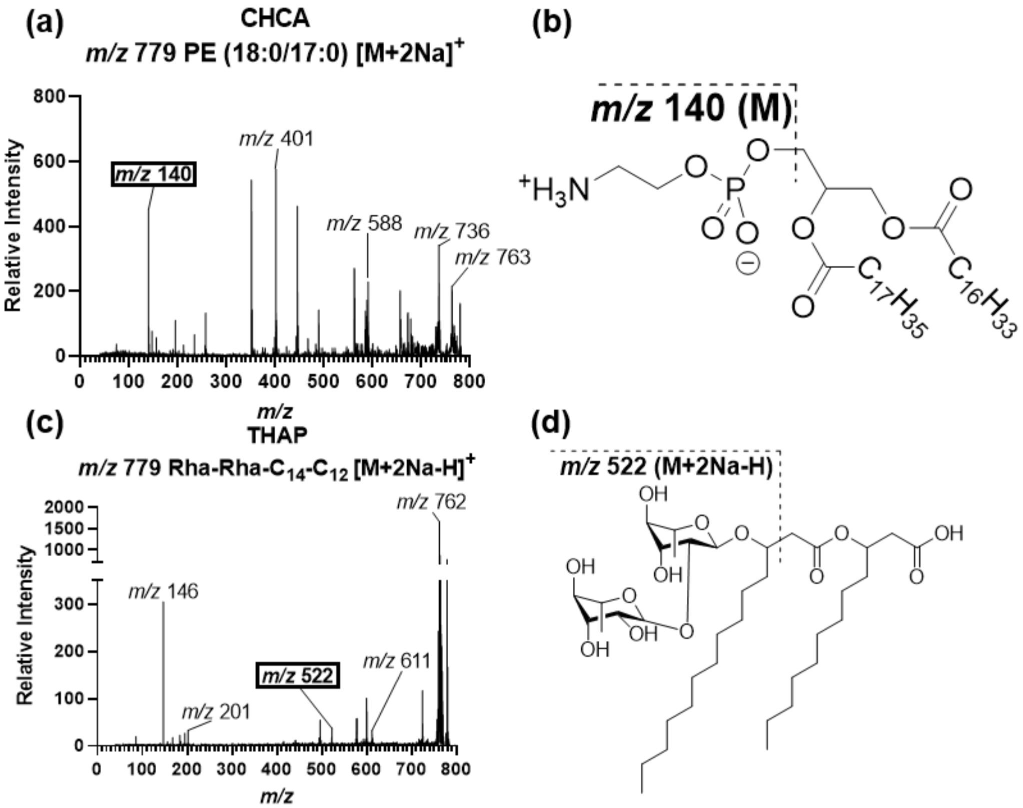

Figure S9. Differences in matrix fragmentation at *m/z* 779 ion. (a) Collected CID spectrum of *m/z* 779 in CHCA identified as PE (18:0/17:0) $[M+2Na]^+$. **(b)** Structure of PE (18:0/17:0). **(c)** Collected spectrum of *m/z* 779 in THAP identified as Rha-Rha-C_{14}-C_{12} $[M+2Na-H]^+$. **(d)** Structure of Rha-Rha-C_{14}-C_{12}.

Table S1. Initial nominal ions of interest scans. Orange highlighted ion values correspond to ions of interest that could not be fragmented by CID analysis. Green highlighted ones correspond to those ions of interest that were identified via CID. *Indicates ion was identified as multiple molecules.

9AA	CHCA	CMBZT	sDHB	HPA	THAP
Total Identified	Total Identified	Total Identified	Total Identified	Total Identified	Total Identified
5/30 (17%)	23/25 (92%)	17/33 (52%)	24/54 (44%)	17/35 (49%)	23/34 (68%)
191.05	190.46	191.13	191.85	212.00	238.42
194.97	198.67	194.98	192.87	215.86	244.53
209.02	211.40	195.99	194.96	231.02	249.42
211.00	219.54	198.89	196.95	233.00	257.60
212.01	230.61	200.84	198.88	235.02	260.65
217.00	260.53	205.99	210.99	260.15	270.70
223.06	263.62	211.03	212.01	268.20	272.71
228.99	270.60	212.05	214.88	268.94	282.58
256.28	272.62	230.95	230.89	270.19	284.81
257.14	272.62*	244.19	232.89	272.21	286.77
268.18	284.74	254.20	235.06	277.06	288.79
277.05	288.63	256.34	244.13	279.07	294.68
279.20	338.69	257.22	254.14	282.17	298.77
284.35	357.64	260.22	256.28	286.20	310.79
291.05	379.68	270.26	257.13	288.23	325.75
299.19	452.97	272.26	260.15	296.12	335.71
314.19	489.00	277.09	266.15	297.03	338.85
338.23	554.17	284.42	270.18	301.09	357.65
372.27	554.17*	288.29	272.21	339.09	359.85
385.28	605.24	304.44	282.15	356.19	379.76
387.30	656.94	304.44*	284.35	361.10	390.85
403.31	698.00	304.44*	286.21	472.23	403.87
474.18	763.58	310.00	288.22	500.26	528.40
476.17	779.58	332.51	294.22	532.23	674.75
740.88	813.19	337.05	298.26	554.24	702.85
756.87		338.30	300.27	556.23	741.24
760.96		403.06	308.20	595.39	742.06
762.89		575.68	310.22	633.36	758.03
782.97		617.81	314.28	671.36	758.03*
809.89		734.68	325.14	677.37	762.04
		761.03	326.32	695.35	763.29
		762.96	332.41	715.36	779.28
		783.08	338.23	747.33	784.13
			360.44	749.33	827.05
			575.62	848.45	
			577.82		
			657.64		
			674.73		
			696.72		
			697.67		
			712.72		
			740.92		
			754.89		
			756.90		
			761.00		
			762.91		
			776.88		
			782.99		
			787.93		
			792.87		
			798.98		
			809.92		
			820.92		
			827.83		

Table S2. Commercial lactone standard spectra and fragments. Collected spectra and fragments of commercial lactone standard that ionized in each of the matrices. The matrices THAP and HPA failed to ionize the standard. The matrix 9AA was not attempted.

CHCA *m/z* 244
N-Hexanoyl-L-Homoserine Lactone Standard
[M+2Na-H]$^+$

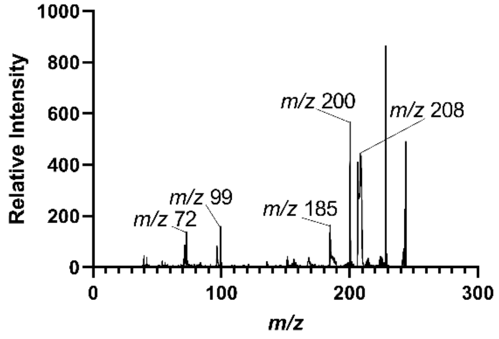

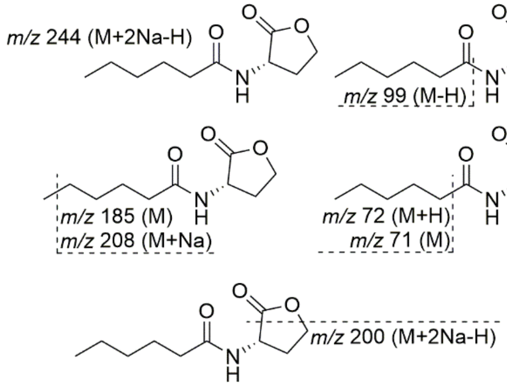

CMBZT *m/z* 222
N-Hexanoyl-L-Homoserine Lactone Standard
[M+Na]$^+$

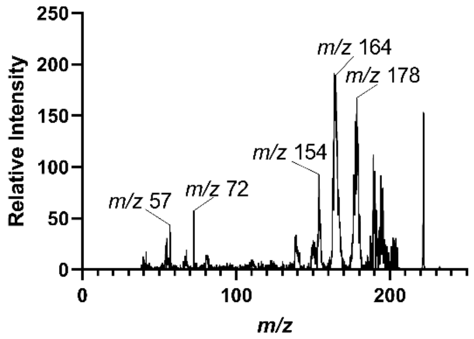

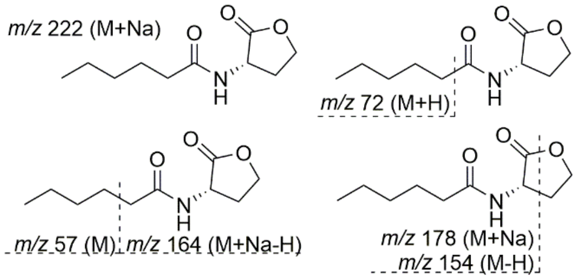

sDHB *m/z* 222
N-Hexanoyl-L-Homoserine Lactone Standard
[M+Na]$^+$

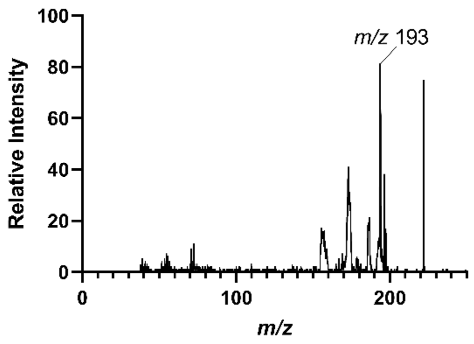

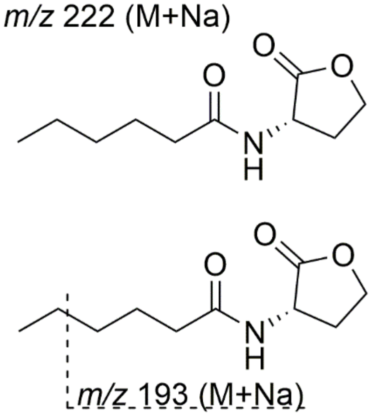

Table S3. Commercial quinolone standards spectra and fragments. Collected spectra and identified fragments of commercial quinolone standards. The matrices HPA and THAP failed to ionize the HHQ standard, 9AA failed to ionize the PQS standard. The matrix 9AA was not used for HHQ and HQNO standards.

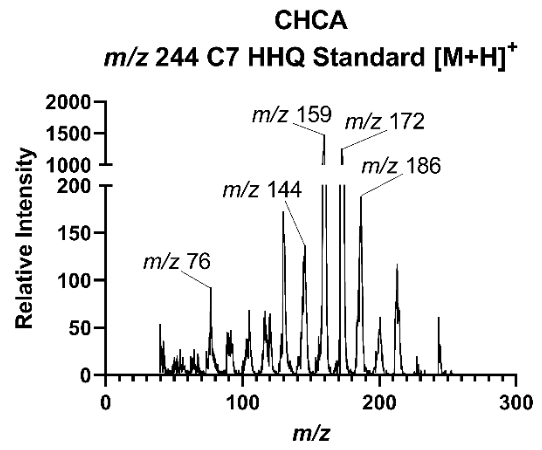

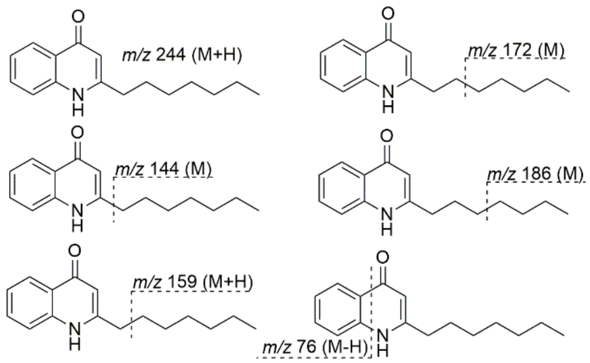

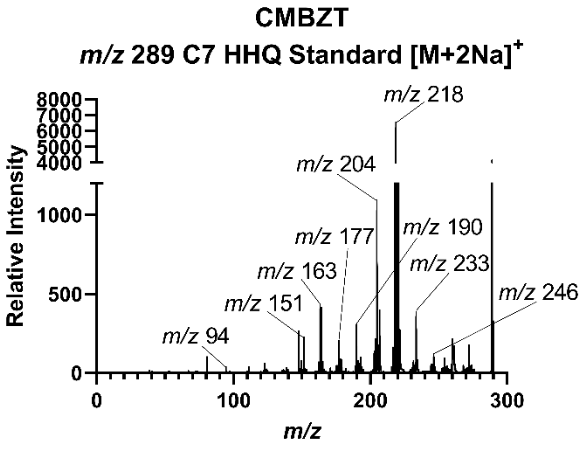

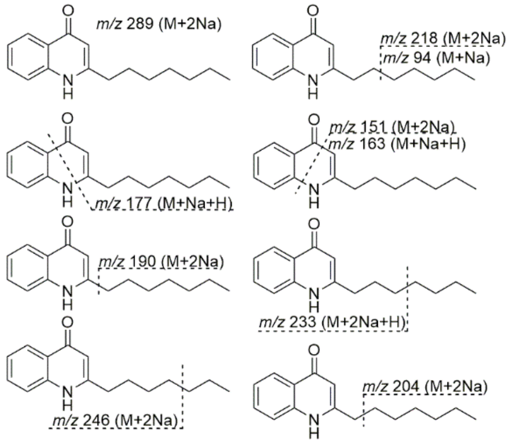

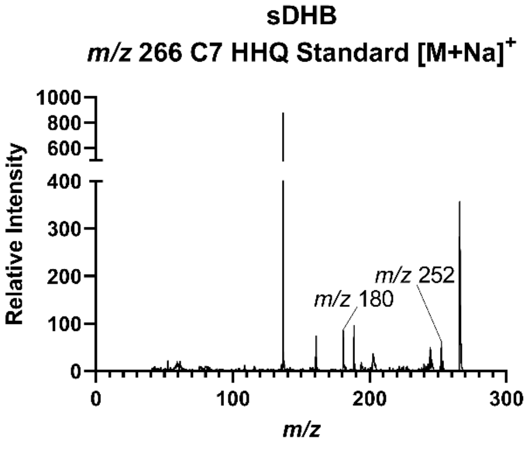

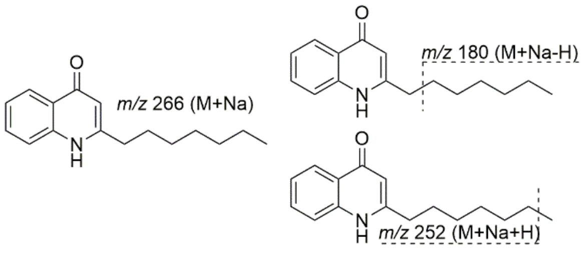

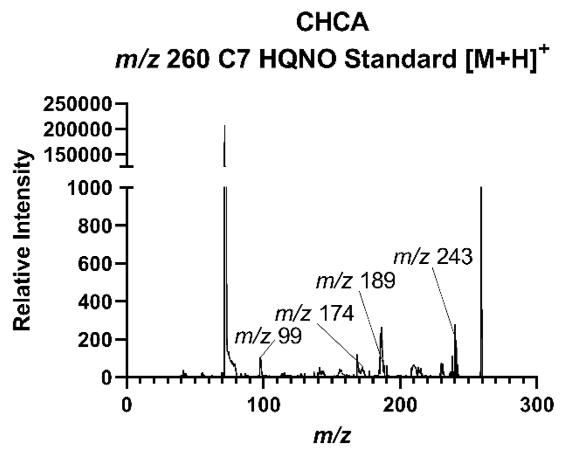
CHCA
m/z 260 C7 HQNO Standard [M+H]+
Relative Intensity
m/z
m/z 243
m/z 189
m/z 174
m/z 99
250000
200000
150000
1000
800
600
400
200
0
0
100
200
300

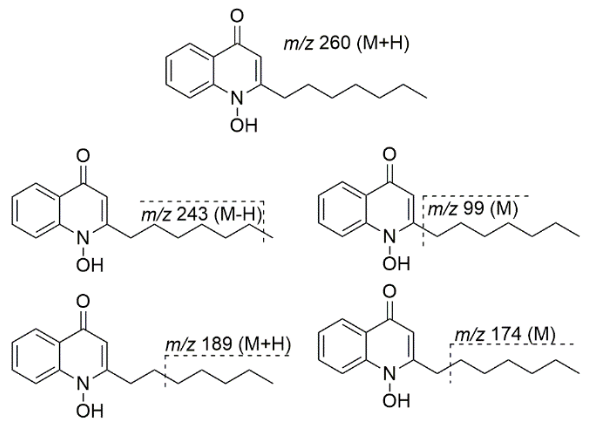
m/z 260 (M+H)
m/z 243 (M-H)
m/z 99 (M)
m/z 189 (M+H)
m/z 174 (M)
O
N
OH

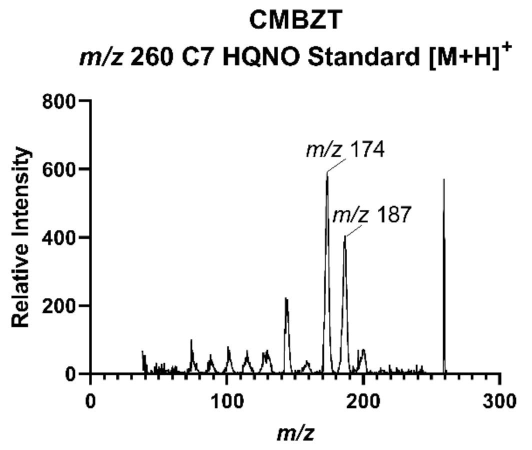
CMBZT
m/z 260 C7 HQNO Standard [M+H]+
Relative Intensity
m/z
m/z 174
m/z 187
800
600
400
200
0
0
100
200
300

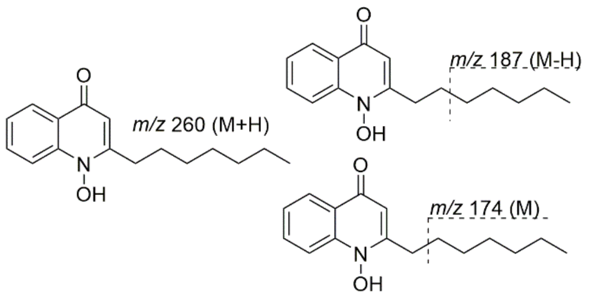
m/z 260 (M+H)
m/z 187 (M-H)
m/z 174 (M)
O
N
OH

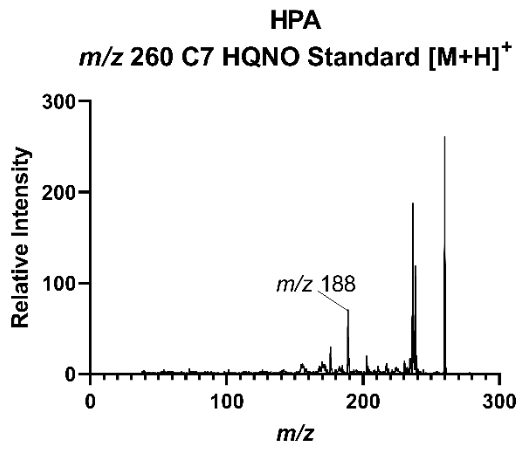
HPA
m/z 260 C7 HQNO Standard [M+H]+
Relative Intensity
m/z
m/z 188
300
200
100
0
0
100
200
300

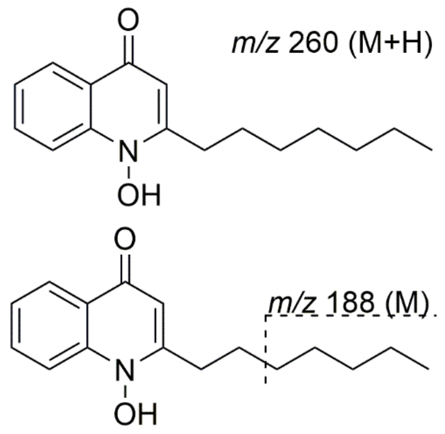
m/z 260 (M+H)
m/z 188 (M)
O
N
OH

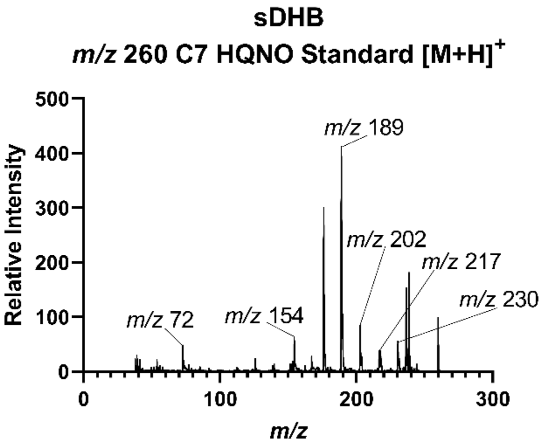
sDHB
m/z 260 C7 HQNO Standard [M+H]+
Relative Intensity
m/z
m/z 189
m/z 202
m/z 217
m/z 230
m/z 72
m/z 154

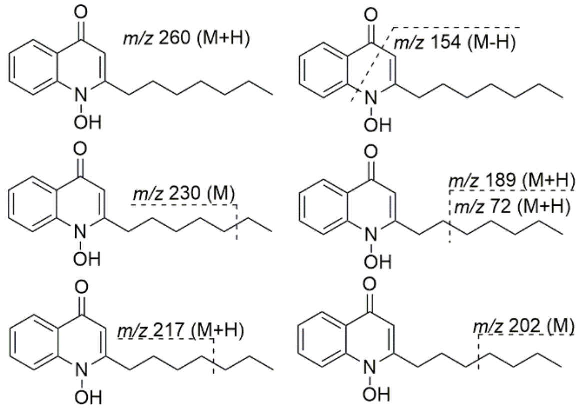
m/z 260 (M+H)
m/z 154 (M-H)
m/z 230 (M)
m/z 189 (M+H)
m/z 72 (M+H)
m/z 217 (M+H)
m/z 202 (M)

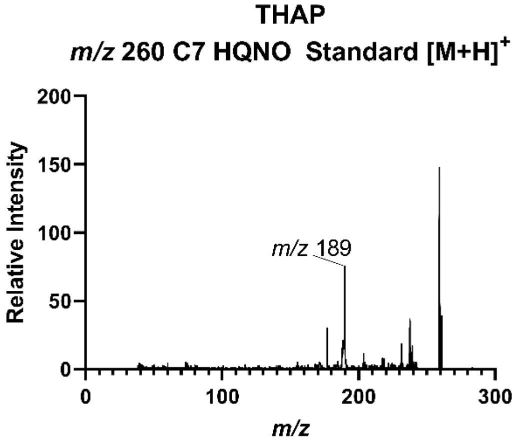
THAP
m/z 260 C7 HQNO Standard [M+H]+
Relative Intensity
m/z
m/z 189

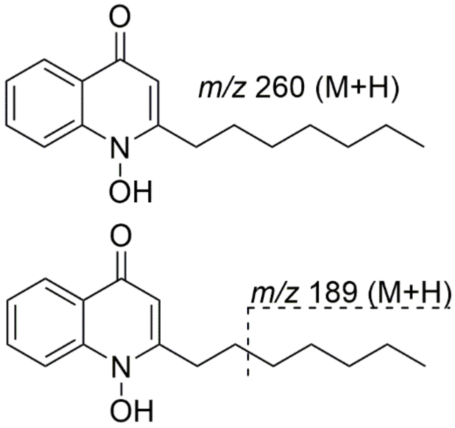
m/z 260 (M+H)
m/z 189 (M+H)

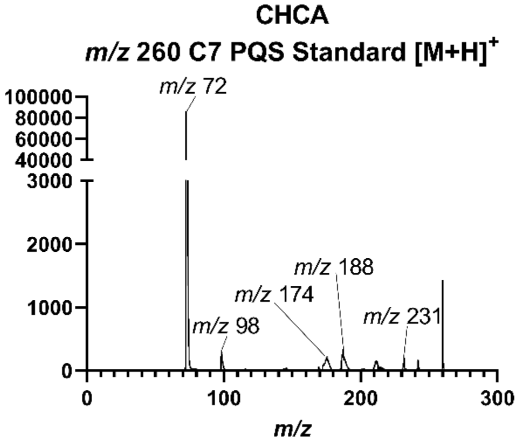
CHCA
m/z 260 C7 PQS Standard [M+H]+
m/z
m/z 72
m/z 188
m/z 174
m/z 98
m/z 231

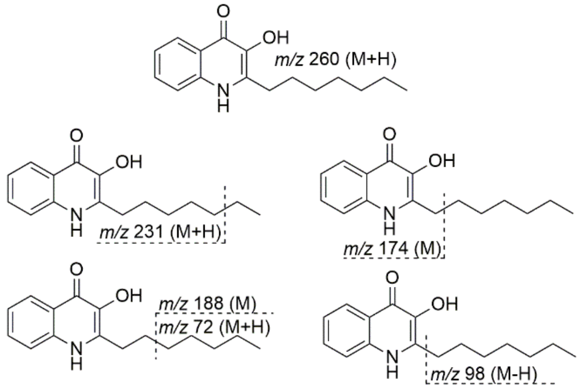
m/z 260 (M+H)
m/z 231 (M+H)
m/z 174 (M)
m/z 188 (M)
m/z 72 (M+H)
m/z 98 (M-H)

CMBZT

***m/z* 282 C7 PQS Standard [M+Na]$^+$**

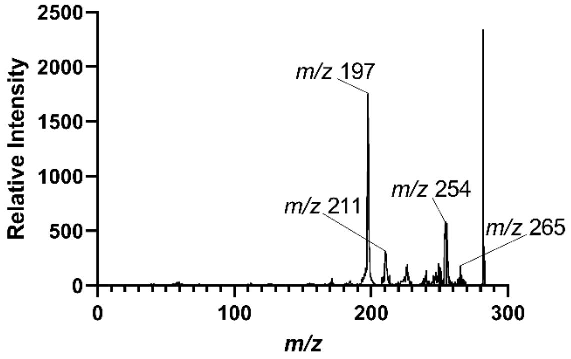

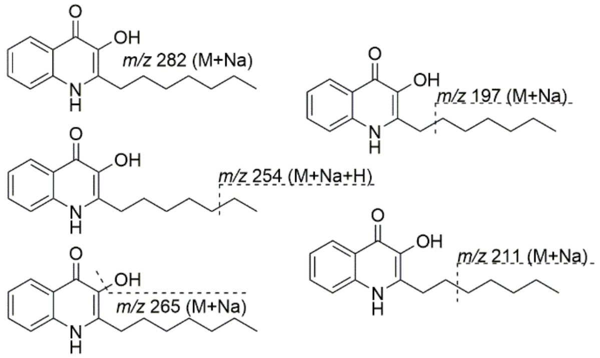

HPA

***m/z* 260 C7 PQS Standard [M+H]$^+$**

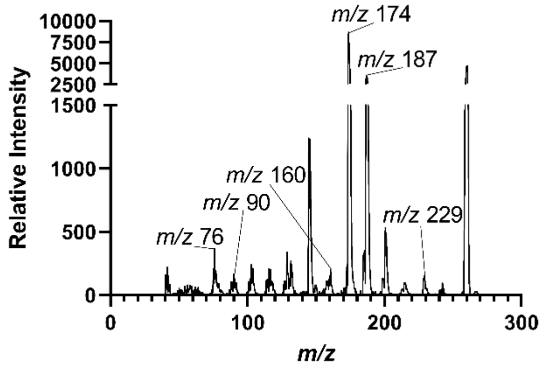

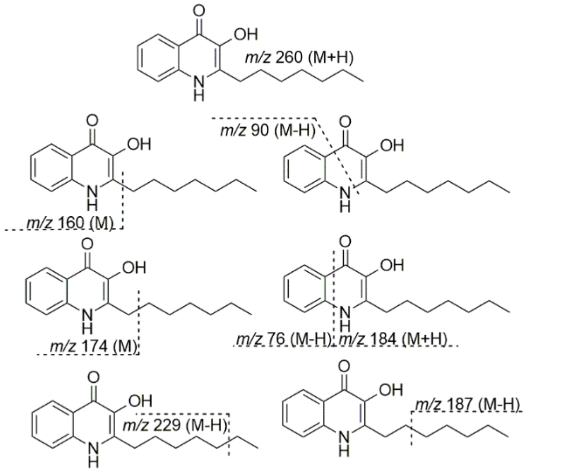

sDHB

***m/z* 260 C7 PQS Standard [M+H]$^+$**

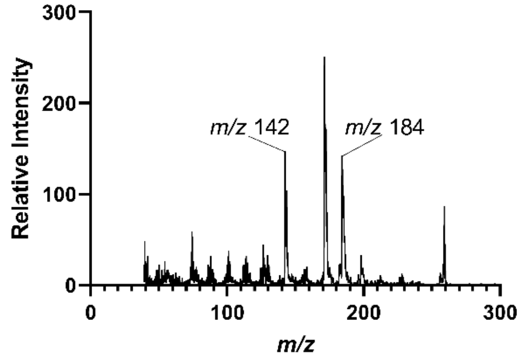

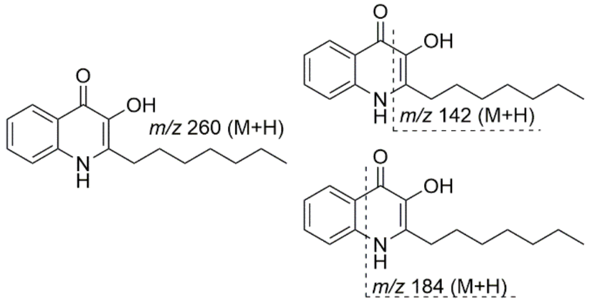

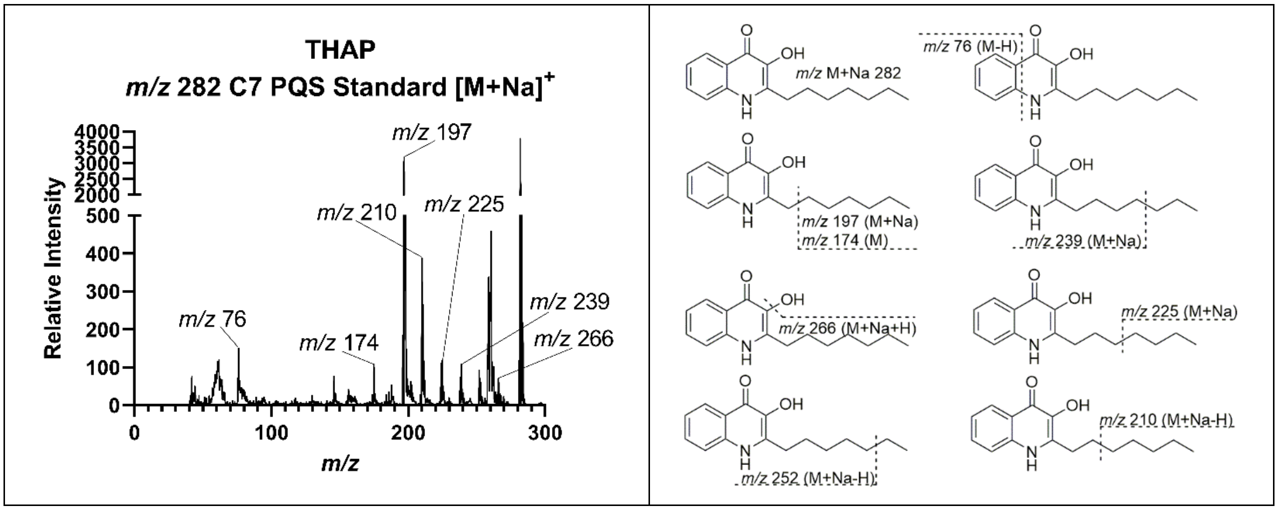
THAP
m/z 282 C7 PQS Standard [M+Na]+
Relative Intensity
m/z
m/z 76
m/z 174
m/z 197
m/z 210
m/z 225
m/z 239
m/z 266
0
100
200
300
400
500
2000
2500
3000
3500
4000
m/z M+Na 282
m/z 76 (M-H)
m/z 197 (M+Na)
m/z 174 (M)
m/z 239 (M+Na)
m/z 266 (M+Na+H)
m/z 225 (M+Na)
m/z 252 (M+Na-H)
m/z 210 (M+Na-H)

Table S4. Commercial phospholipid standards spectra and fragments. Collected spectra and identified fragments for phospholipid and lysophospholipids standards. The lysophospholipids standard was looked at with CHCA, HPA, and 9AA due to relevance to the matrix for the compound in this analysis.

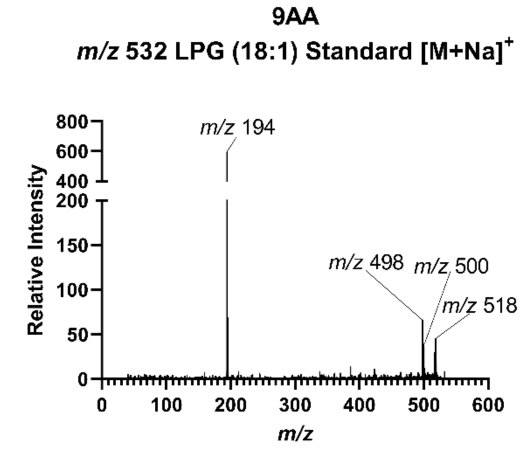

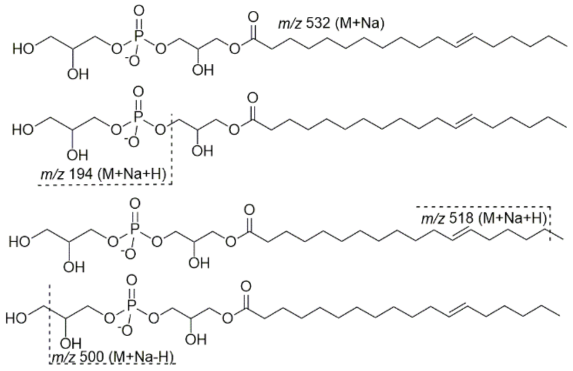

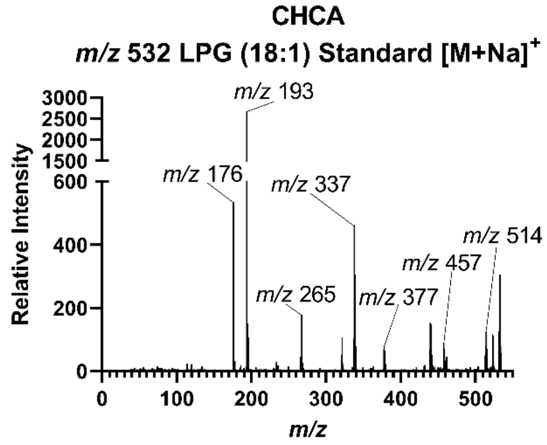

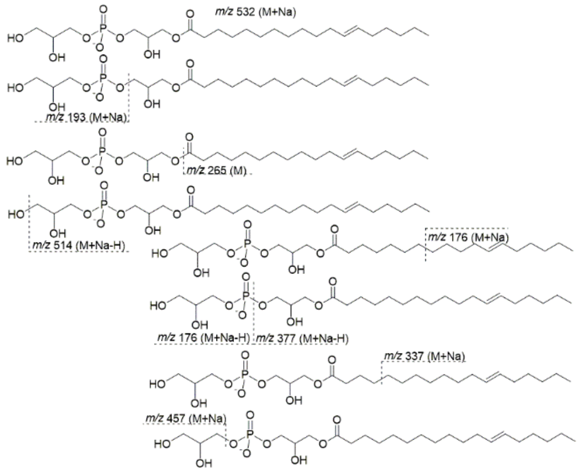

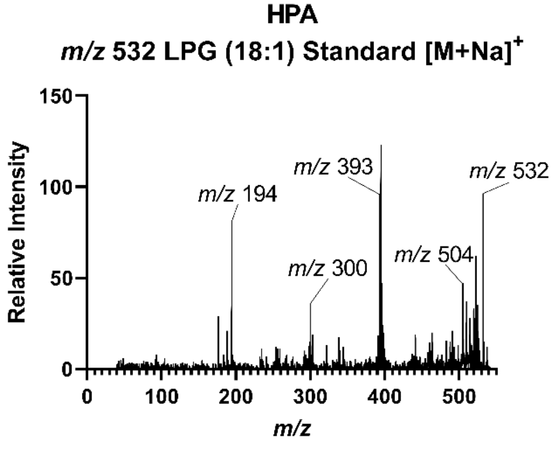

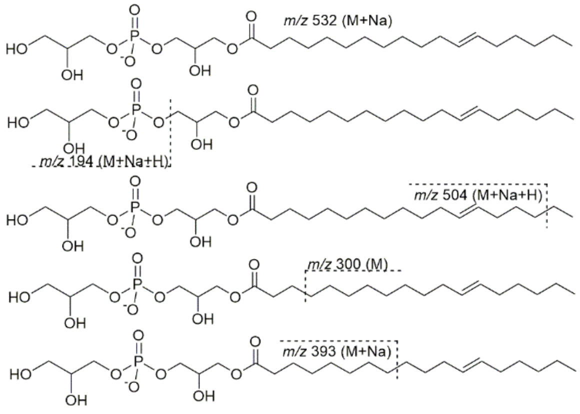

9AA

***m/z* 696 PA (18:1/16:0) Standard [M+Na]$^+$**

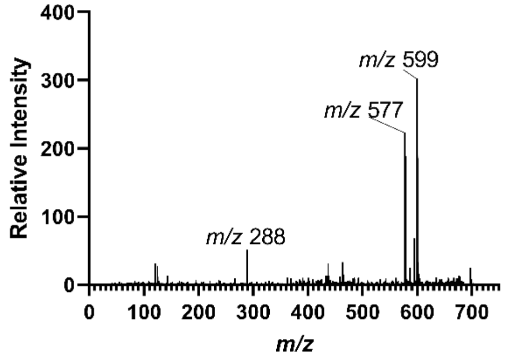

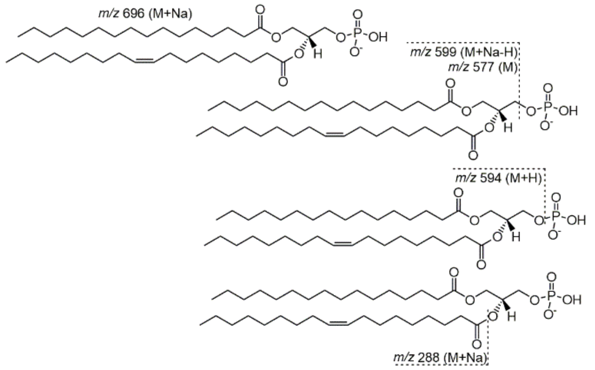

CHCA

***m/z* 696 PA (18:1/16:0) Standard [M+Na]$^+$**

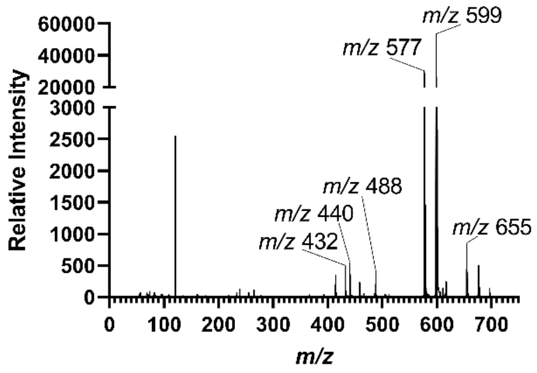

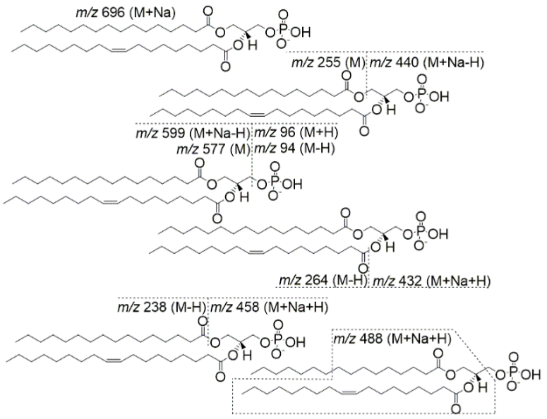

CMBZT

***m/z* 696 PA (18:1/16:0) Standard [M+Na]$^+$**

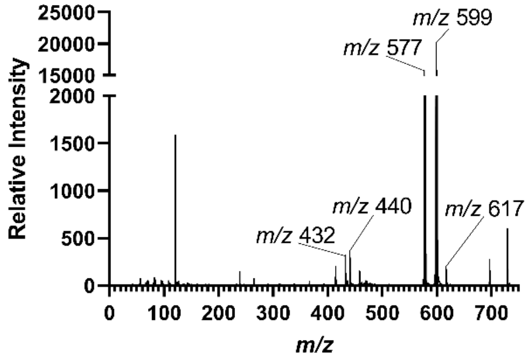

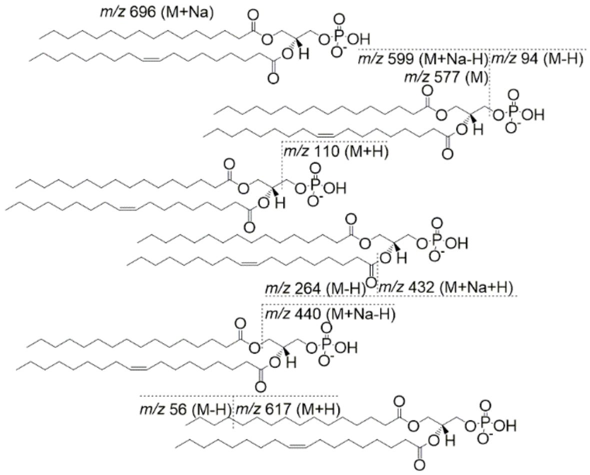

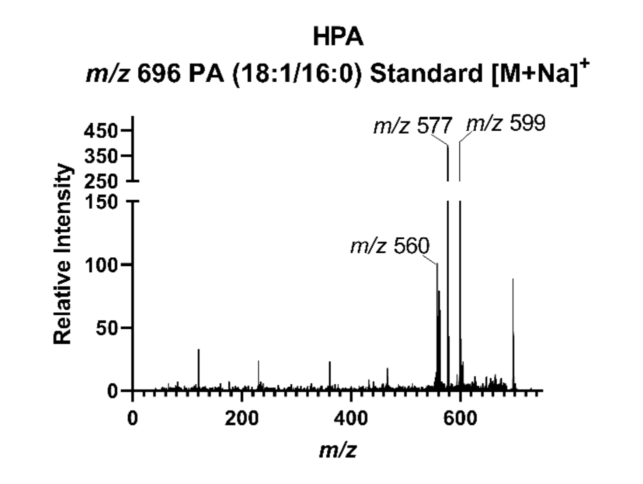
HPA
m/z 696 PA (18:1/16:0) Standard [M+Na]+
Relative Intensity
m/z
m/z 577
m/z 599
m/z 560
450
350
250
150
100
50
0
0
200
400
600

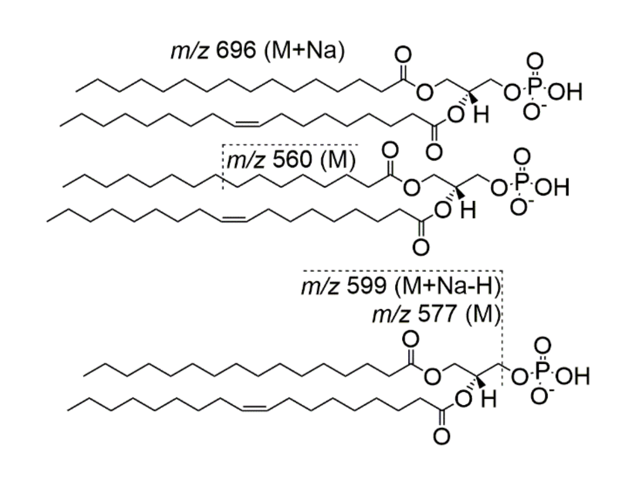
m/z 696 (M+Na)
m/z 560 (M)
m/z 599 (M+Na-H)
m/z 577 (M)

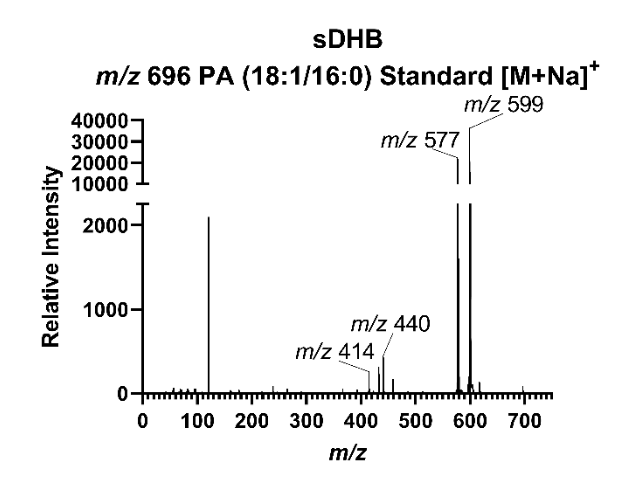
sDHB
m/z 696 PA (18:1/16:0) Standard [M+Na]+
Relative Intensity
m/z
m/z 599
m/z 577
m/z 440
m/z 414
40000
30000
20000
10000
2000
1000
0
0
100
200
300
400
500
600
700

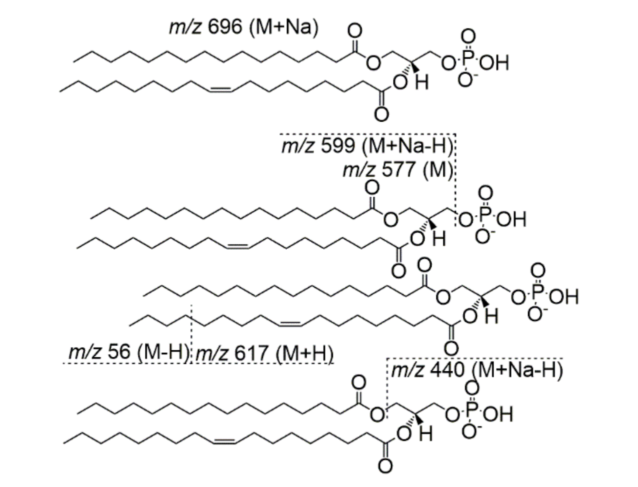
m/z 696 (M+Na)
m/z 599 (M+Na-H)
m/z 577 (M)
m/z 56 (M-H)
m/z 617 (M+H)
m/z 440 (M+Na-H)

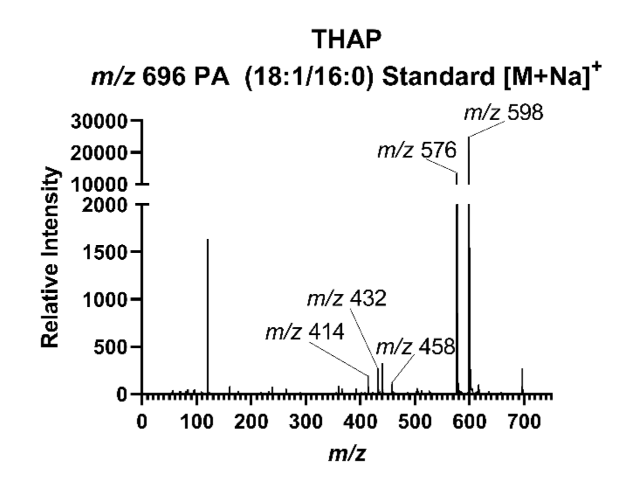
THAP
m/z 696 PA (18:1/16:0) Standard [M+Na]+
Relative Intensity
m/z
m/z 598
m/z 576
m/z 432
m/z 414
m/z 458
30000
20000
10000
2000
1500
1000
500
0
0
100
200
300
400
500
600
700

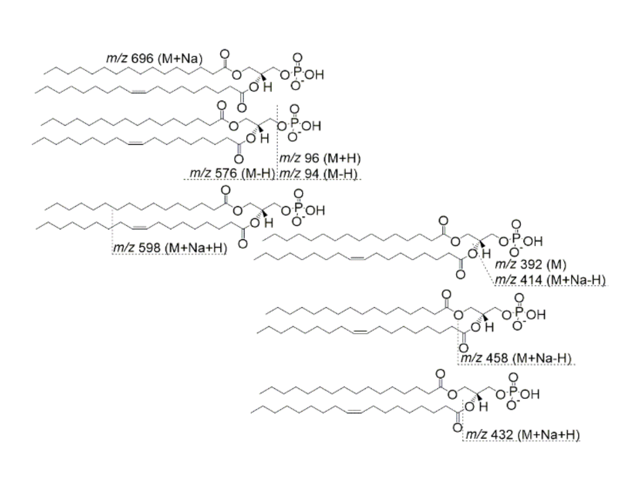
m/z 696 (M+Na)
m/z 96 (M+H)
m/z 576 (M-H)
m/z 94 (M-H)
m/z 598 (M+Na+H)
m/z 392 (M)
m/z 414 (M+Na-H)
m/z 458 (M+Na-H)
m/z 432 (M+Na+H)

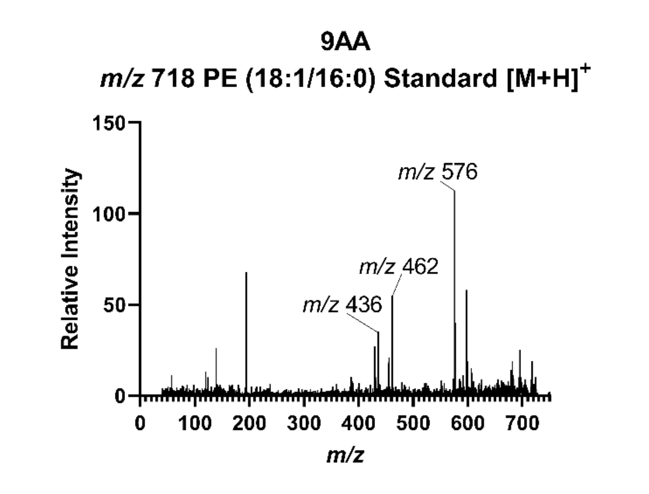
9AA
m/z 718 PE (18:1/16:0) Standard [M+H]+
Relative Intensity
m/z
m/z 576
m/z 462
m/z 436
0
50
100
150
0 100 200 300 400 500 600 700

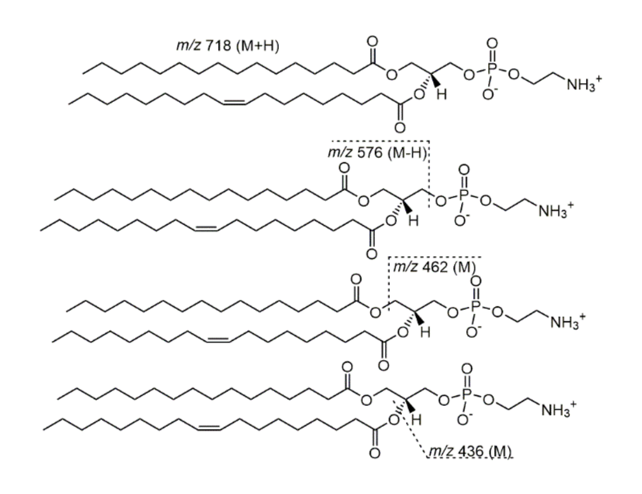
m/z 718 (M+H)
m/z 576 (M-H)
m/z 462 (M)
m/z 436 (M)
NH3+

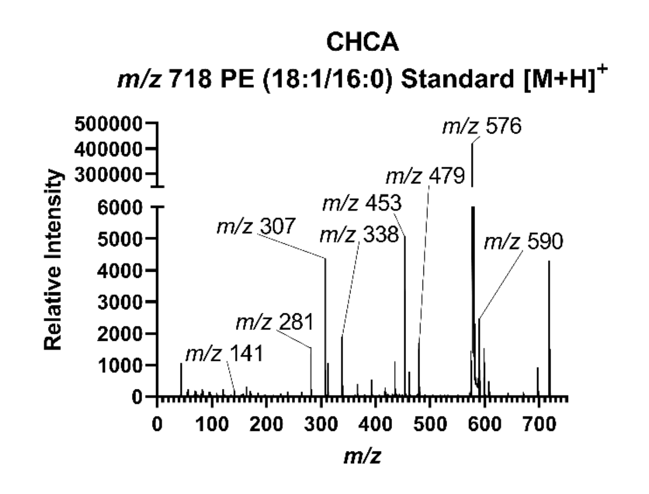
CHCA
m/z 718 PE (18:1/16:0) Standard [M+H]+
Relative Intensity
m/z
m/z 576
m/z 479
m/z 453
m/z 307
m/z 338
m/z 590
m/z 281
m/z 141
0
1000
2000
3000
4000
5000
6000
300000
400000
500000
0 100 200 300 400 500 600 700

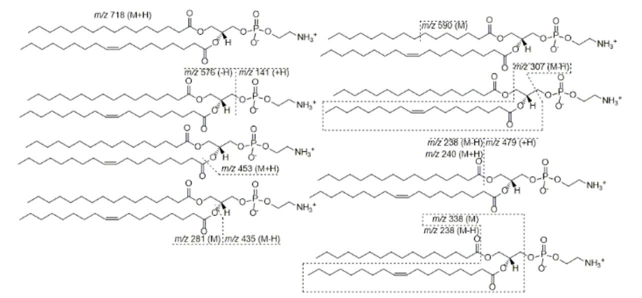
m/z 718 (M+H)
m/z 590 (M)
m/z 307 (M-H)
m/z 453 (M+H)
m/z 479 (+H)
m/z 338 (M)
m/z 281 (M)
NH3+

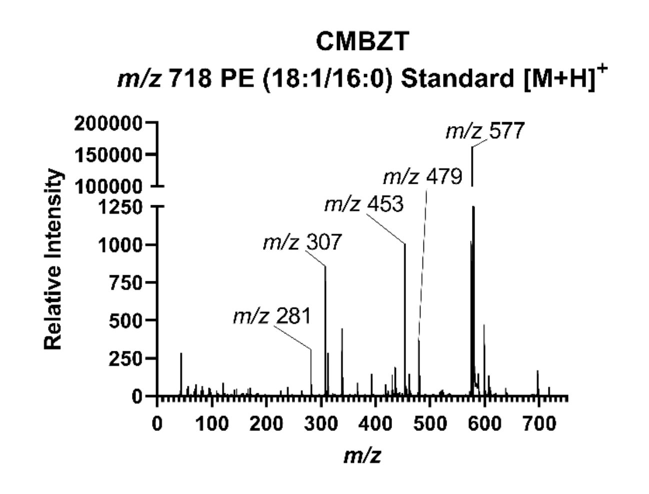
CMBZT
m/z 718 PE (18:1/16:0) Standard [M+H]+
Relative Intensity
m/z
m/z 577
m/z 479
m/z 453
m/z 307
m/z 281
0
250
500
750
1000
1250
100000
150000
200000
0 100 200 300 400 500 600 700

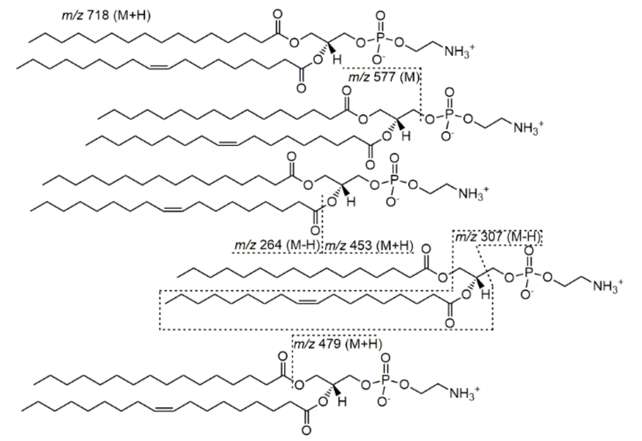
m/z 718 (M+H)
m/z 577 (M)
m/z 264 (M-H)
m/z 453 (M+H)
m/z 307 (M-H)
m/z 479 (M+H)
NH3+

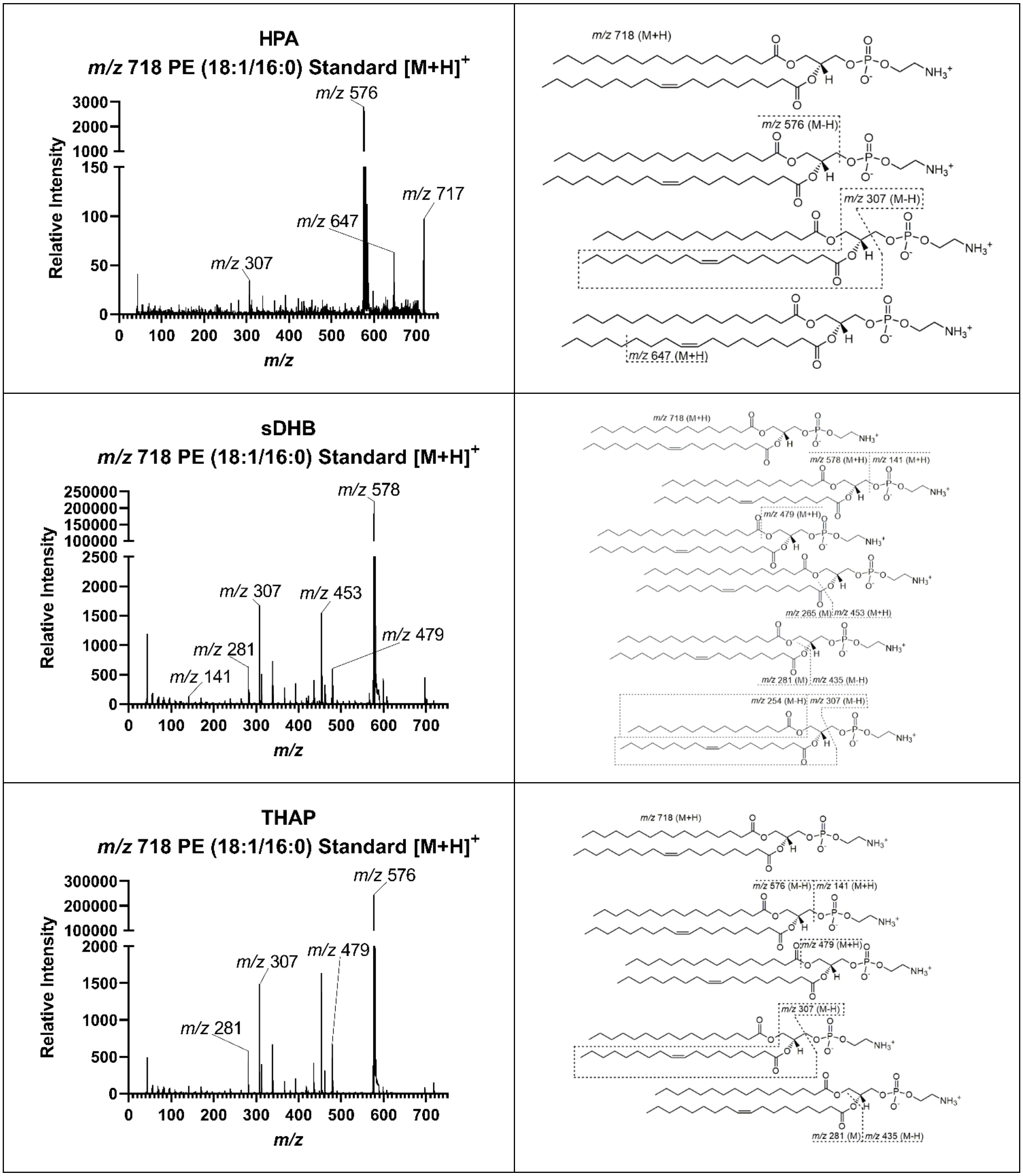
HPA
m/z 718 PE (18:1/16:0) Standard [M+H]+
Relative Intensity
m/z
m/z 576
m/z 717
m/z 647
m/z 307
m/z 718 (M+H)
m/z 576 (M-H)
m/z 307 (M-H)
m/z 647 (M+H)
sDHB
m/z 718 PE (18:1/16:0) Standard [M+H]+
m/z 578
m/z 307
m/z 453
m/z 479
m/z 281
m/z 141
m/z 578 (M+H)
m/z 141 (M+H)
m/z 479 (M+H)
m/z 265 (M)
m/z 453 (M+H)
m/z 281 (M)
m/z 435 (M-H)
m/z 254 (M-H)
THAP
m/z 718 PE (18:1/16:0) Standard [M+H]+
m/z 576
m/z 307
m/z 479
m/z 281
m/z 576 (M-H)
m/z 141 (M+H)
m/z 479 (M+H)
m/z 307 (M-H)
m/z 281 (M)
m/z 435 (M-H)

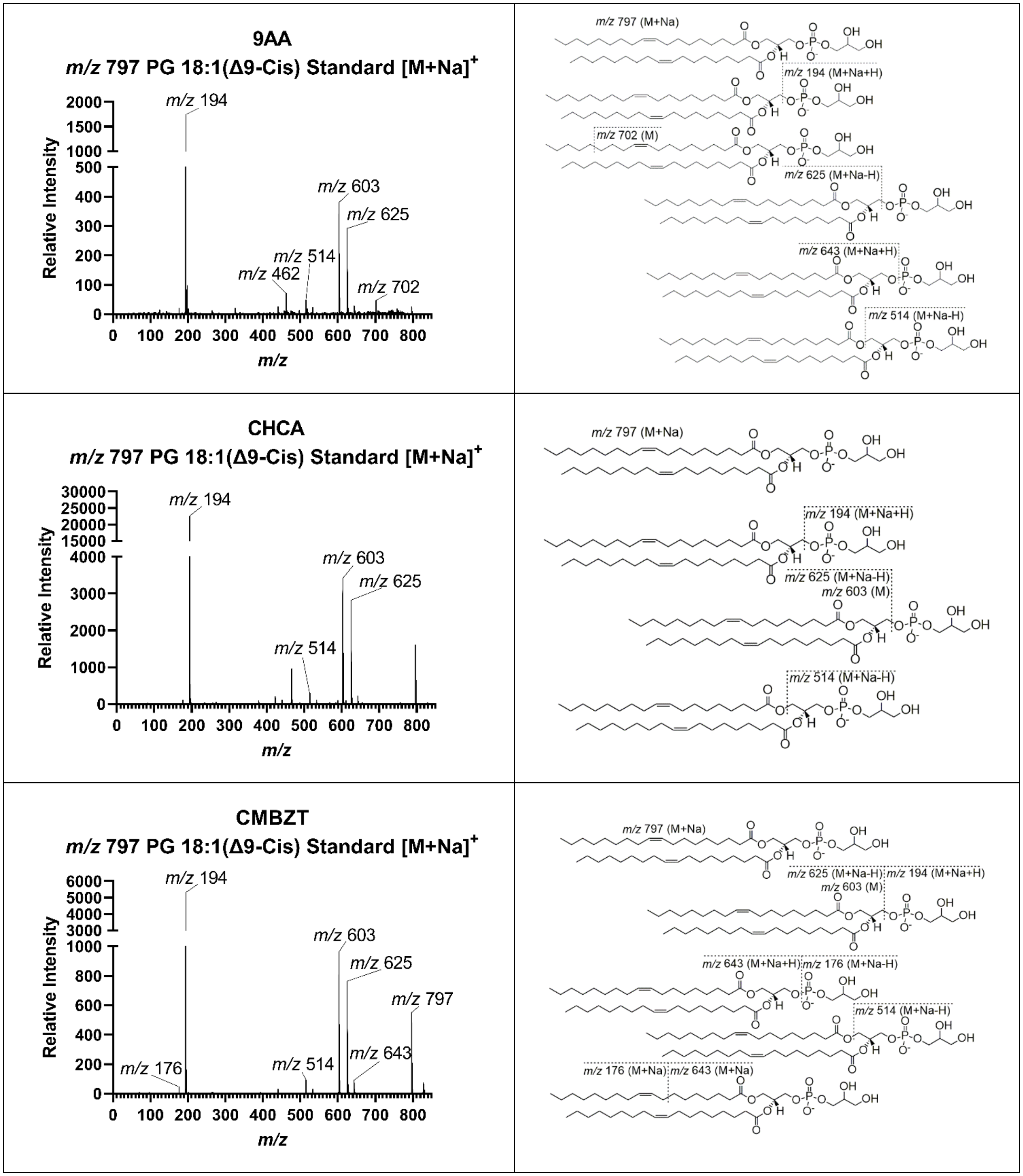
9AA
m/z 797 PG 18:1(Δ9-Cis) Standard [M+Na]+
Relative Intensity
m/z
m/z 194
m/z 603
m/z 625
m/z 514
m/z 462
m/z 702
m/z 797 (M+Na)
m/z 194 (M+Na+H)
m/z 702 (M)
m/z 625 (M+Na-H)
m/z 643 (M+Na+H)
m/z 514 (M+Na-H)
CHCA
m/z 797 PG 18:1(Δ9-Cis) Standard [M+Na]+
Relative Intensity
m/z
m/z 194
m/z 603
m/z 625
m/z 514
m/z 797 (M+Na)
m/z 194 (M+Na+H)
m/z 625 (M+Na-H)
m/z 603 (M)
m/z 514 (M+Na-H)
CMBZT
m/z 797 PG 18:1(Δ9-Cis) Standard [M+Na]+
Relative Intensity
m/z
m/z 194
m/z 603
m/z 625
m/z 797
m/z 176
m/z 514
m/z 643
m/z 797 (M+Na)
m/z 625 (M+Na-H)
m/z 194 (M+Na+H)
m/z 603 (M)
m/z 643 (M+Na+H)
m/z 176 (M+Na-H)
m/z 514 (M+Na-H)
m/z 176 (M+Na)
m/z 643 (M+Na)

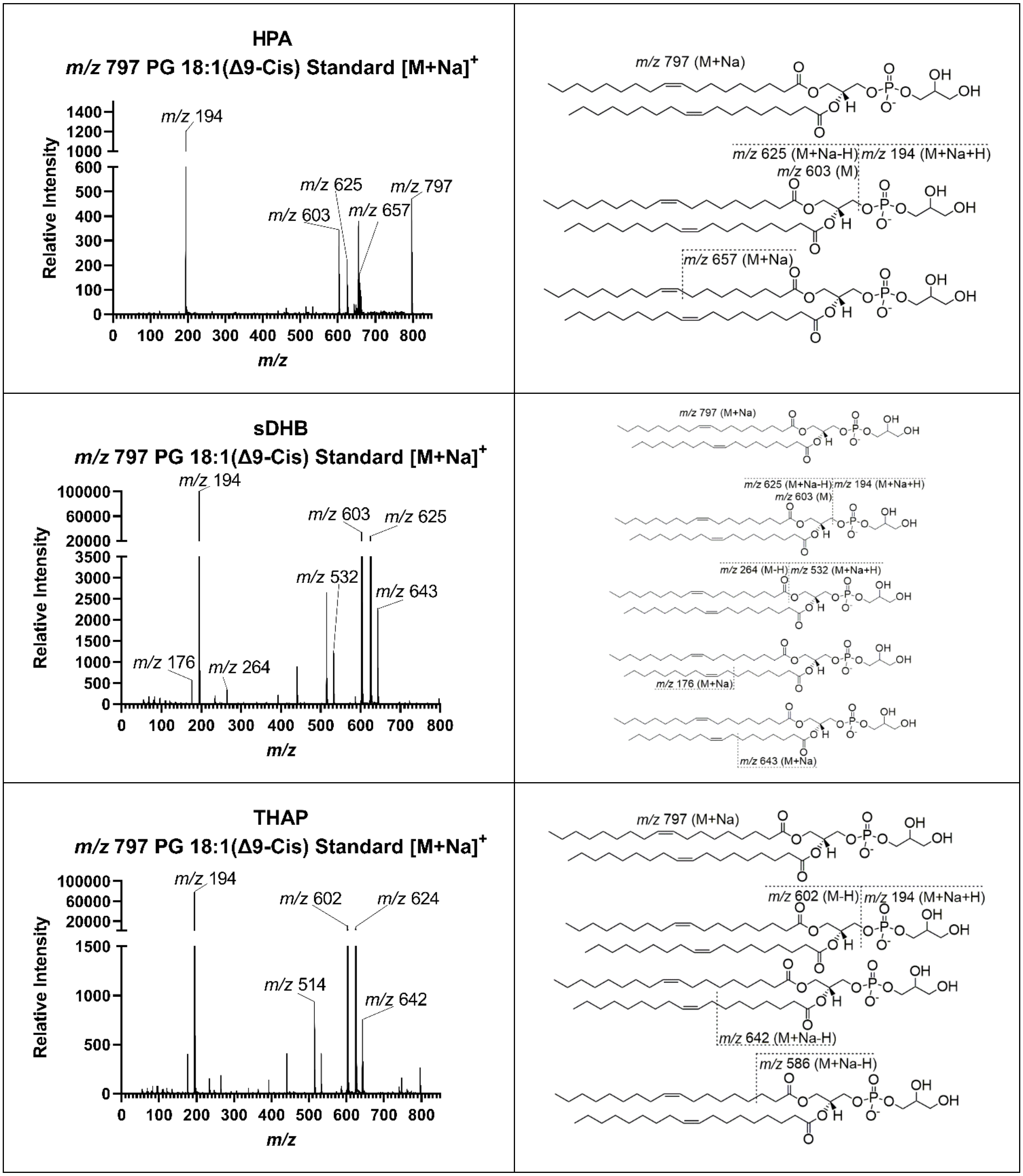
HPA
m/z 797 PG 18:1(Δ9-Cis) Standard [M+Na]+
Relative Intensity
m/z
m/z 194
m/z 625
m/z 797
m/z 603
m/z 657
m/z 797 (M+Na)
m/z 625 (M+Na-H)
m/z 194 (M+Na+H)
m/z 603 (M)
m/z 657 (M+Na)
sDHB
m/z 797 PG 18:1(Δ9-Cis) Standard [M+Na]+
m/z 194
m/z 603
m/z 625
m/z 532
m/z 643
m/z 176
m/z 264
m/z 797 (M+Na)
m/z 625 (M+Na-H)
m/z 194 (M+Na+H)
m/z 603 (M)
m/z 264 (M-H)
m/z 532 (M+Na+H)
m/z 176 (M+Na)
m/z 643 (M+Na)
THAP
m/z 797 PG 18:1(Δ9-Cis) Standard [M+Na]+
m/z 194
m/z 602
m/z 624
m/z 514
m/z 642
m/z 797 (M+Na)
m/z 602 (M-H)
m/z 194 (M+Na+H)
m/z 642 (M+Na-H)
m/z 586 (M+Na-H)

Table S5. Commercial rhamnolipid standards spectra and fragments. Collected spectra and identified fragments for mono-rhamnolipid and di-rhamnolipid standards. THAP and 9AA were not analyzed for these standards.

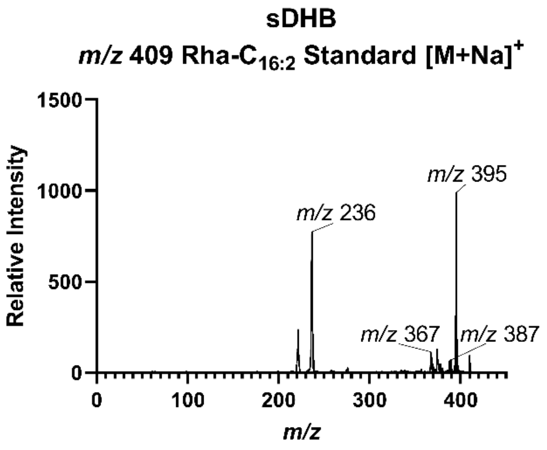

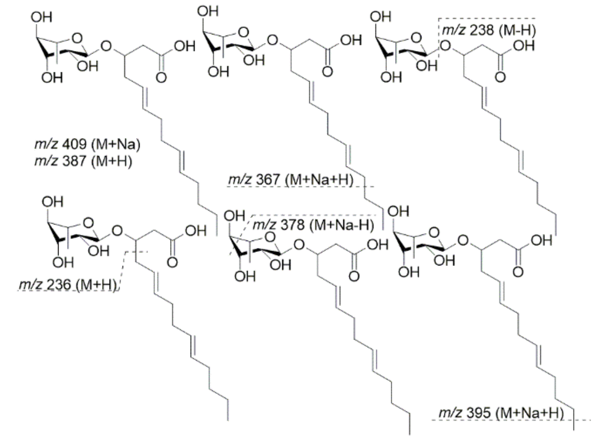

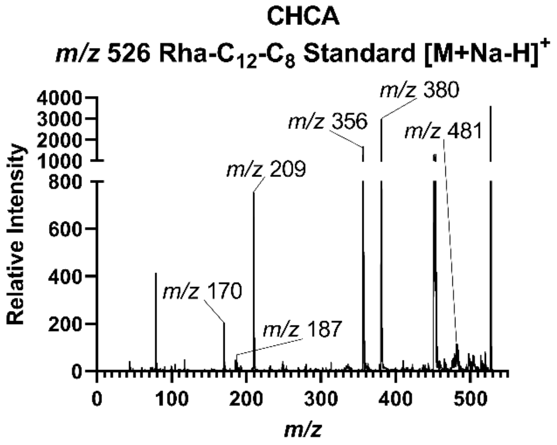

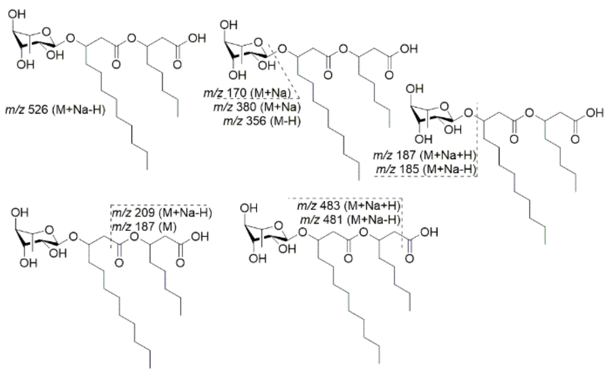

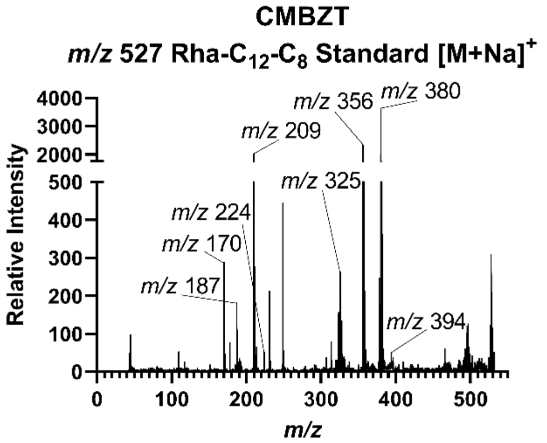

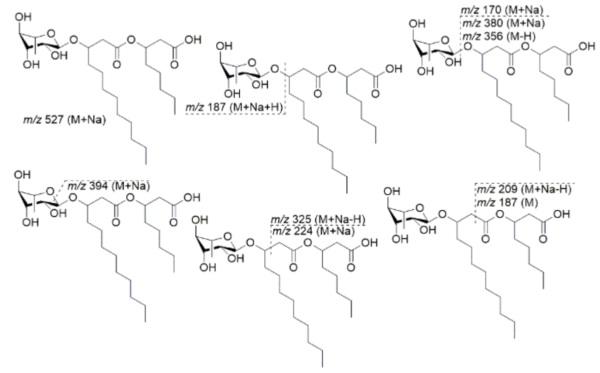

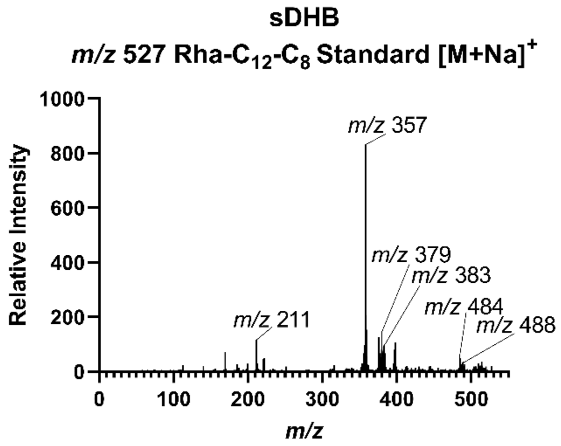
sDHB
m/z 527 Rha-C12-C8 Standard [M+Na]+
Relative Intensity
m/z
m/z 357
m/z 379
m/z 383
m/z 484
m/z 488
m/z 211

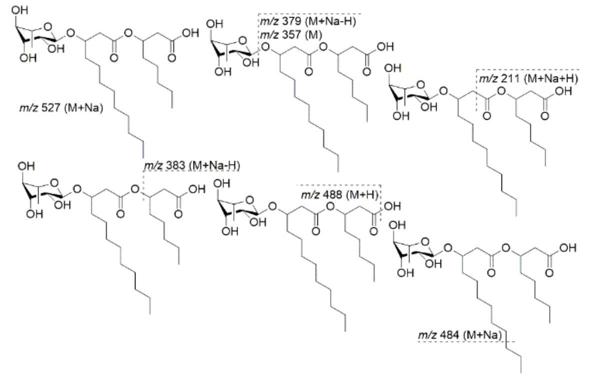
m/z 527 (M+Na)
m/z 379 (M+Na-H)
m/z 357 (M)
m/z 211 (M+Na+H)
m/z 383 (M+Na-H)
m/z 488 (M+H)
m/z 484 (M+Na)

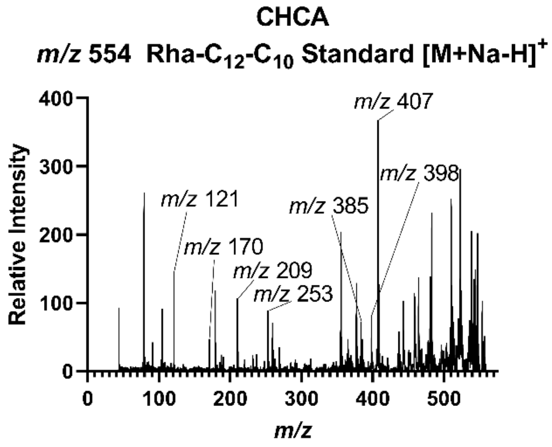
CHCA
m/z 554 Rha-C12-C10 Standard [M+Na-H]+
Relative Intensity
m/z
m/z 407
m/z 398
m/z 121
m/z 385
m/z 170
m/z 209
m/z 253

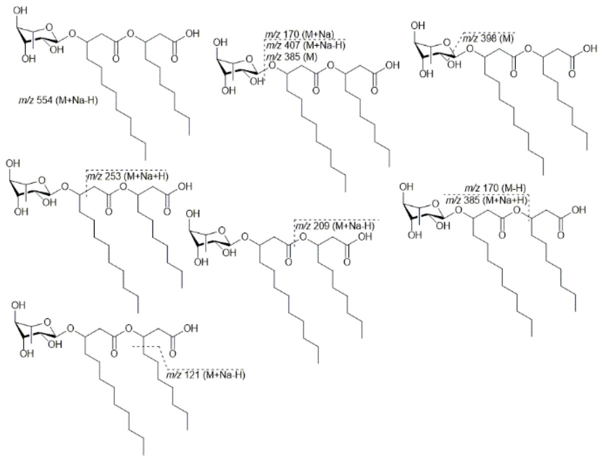
m/z 554 (M+Na-H)
m/z 170 (M+Na)
m/z 407 (M+Na-H)
m/z 385 (M)
m/z 398 (M)
m/z 253 (M+Na+H)
m/z 209 (M+Na-H)
m/z 170 (M-H)
m/z 385 (M+Na+H)
m/z 121 (M+Na-H)

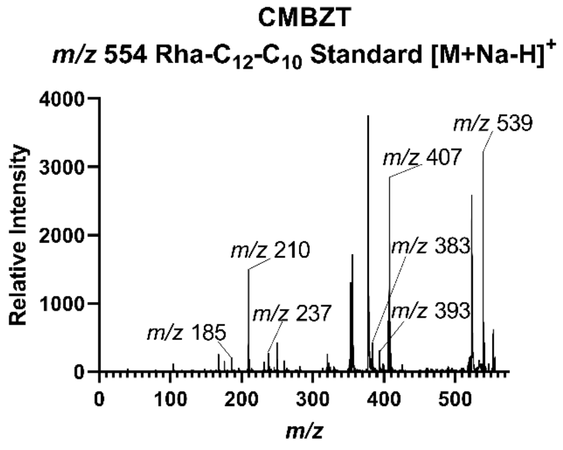
CMBZT
m/z 554 Rha-C12-C10 Standard [M+Na-H]+
Relative Intensity
m/z
m/z 539
m/z 407
m/z 210
m/z 383
m/z 237
m/z 393
m/z 185

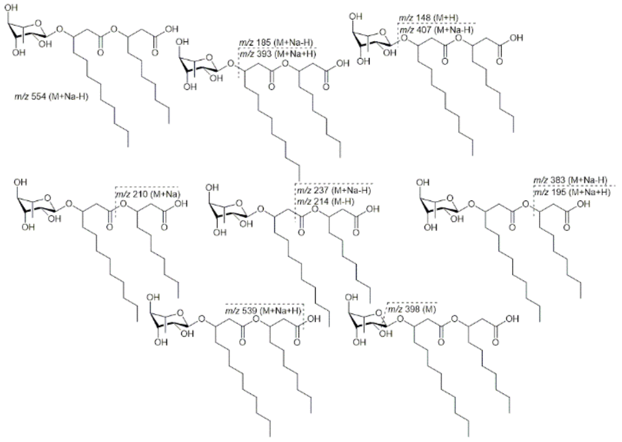
m/z 554 (M+Na-H)
m/z 185 (M+Na-H)
m/z 393 (M+Na+H)
m/z 148 (M+H)
m/z 407 (M+Na-H)
m/z 210 (M+Na)
m/z 237 (M+Na-H)
m/z 214 (M-H)
m/z 383 (M+Na-H)
m/z 196 (M+Na+H)
m/z 539 (M+Na+H)
m/z 398 (M)

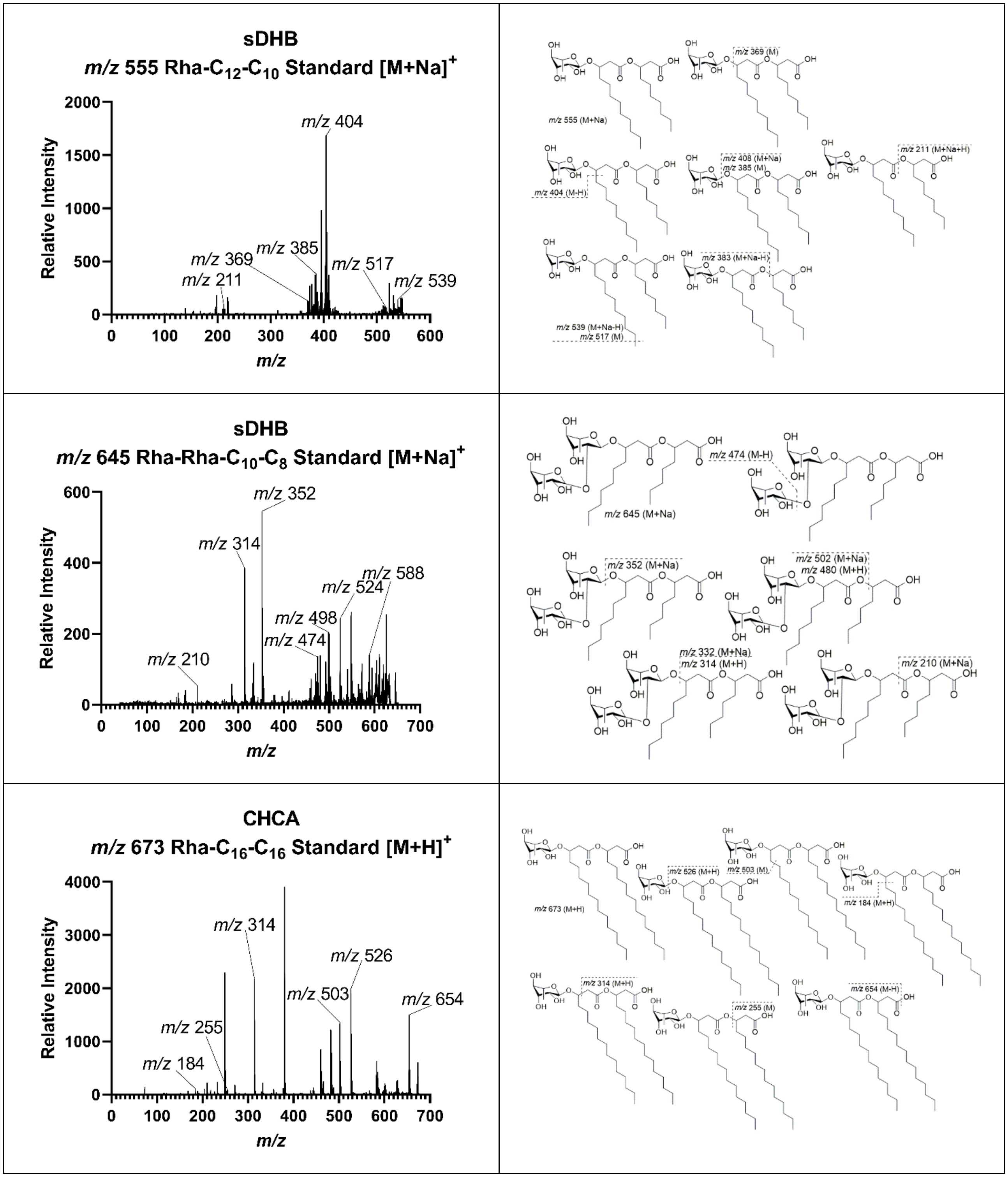

sDHB
m/z 555 Rha-C12-C10 Standard [M+Na]+
Relative Intensity
m/z
m/z 404
m/z 385
m/z 369
m/z 211
m/z 517
m/z 539
m/z 555 (M+Na)
m/z 369 (M)
m/z 404 (M-H)
m/z 408 (M+Na)
m/z 385 (M)
m/z 211 (M+Na+H)
m/z 383 (M+Na-H)
m/z 539 (M+Na-H)
m/z 517 (M)
sDHB
m/z 645 Rha-Rha-C10-C8 Standard [M+Na]+
Relative Intensity
m/z
m/z 352
m/z 314
m/z 588
m/z 524
m/z 498
m/z 474
m/z 210
m/z 645 (M+Na)
m/z 474 (M-H)
m/z 352 (M+Na)
m/z 502 (M+Na)
m/z 480 (M+H)
m/z 332 (M+Na)
m/z 314 (M+H)
m/z 210 (M+Na)
CHCA
m/z 673 Rha-C16-C16 Standard [M+H]+
Relative Intensity
m/z
m/z 314
m/z 526
m/z 503
m/z 654
m/z 255
m/z 184
m/z 673 (M+H)
m/z 526 (M+H)
m/z 503 (M)
m/z 184 (M+H)
m/z 314 (M+H)
m/z 255 (M)
m/z 654 (M-H)

HPA

***m/z* 673 Rha-C_{16}-C_{16} Standard $[M+H]^+$**

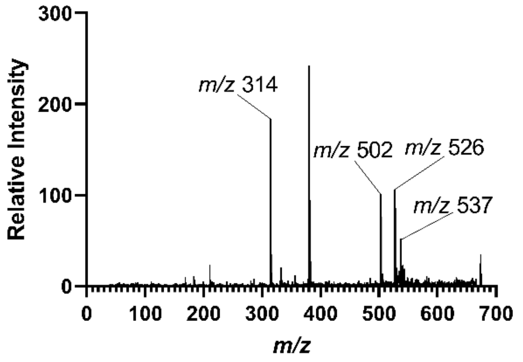

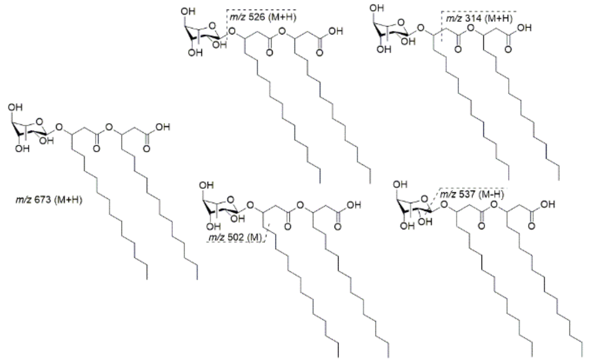

sDHB

***m/z* 673 Rha-C_{16}-C_{16} Standard $[M+H]^+$**

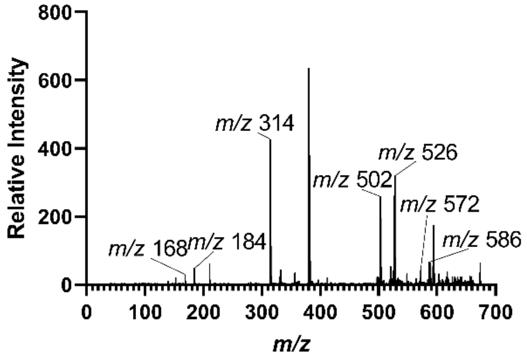

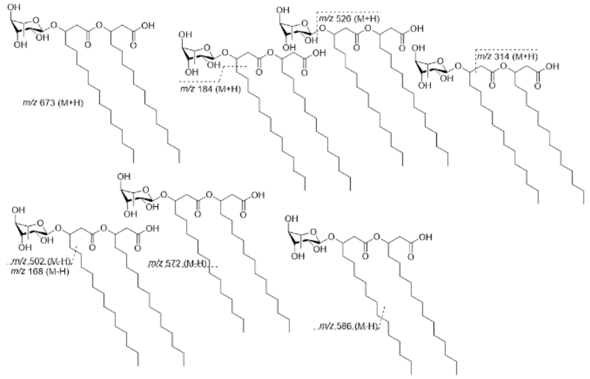

CMBZT

***m/z* 678 Decenoyl-Rha-C_{10}-C_{10}**

Standard $[M+Na-H]^+$

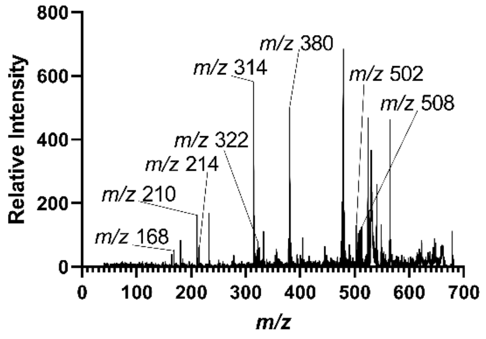

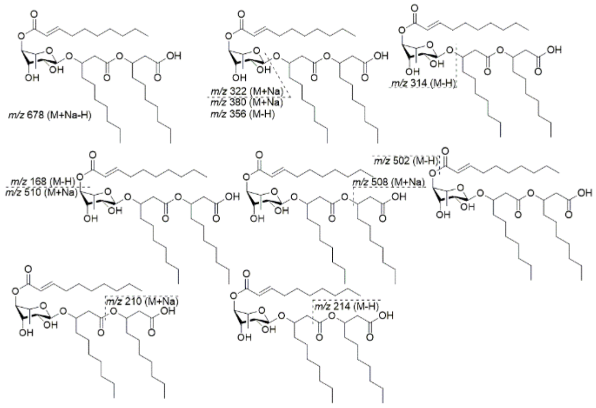

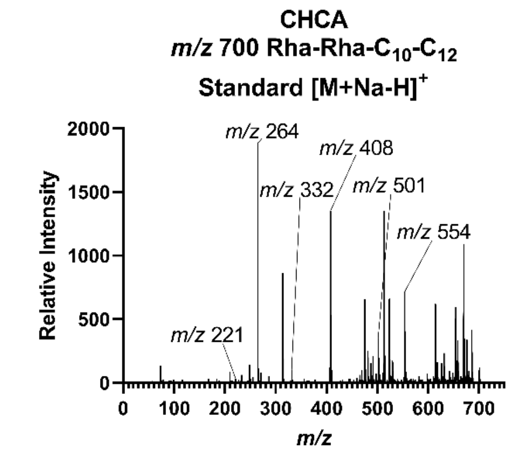
CHCA
m/z 700 Rha-Rha-C10-C12
Standard [M+Na-H]+
Relative Intensity
m/z
m/z 264
m/z 408
m/z 332
m/z 501
m/z 554
m/z 221
2000
1500
1000
500
0
0 100 200 300 400 500 600 700

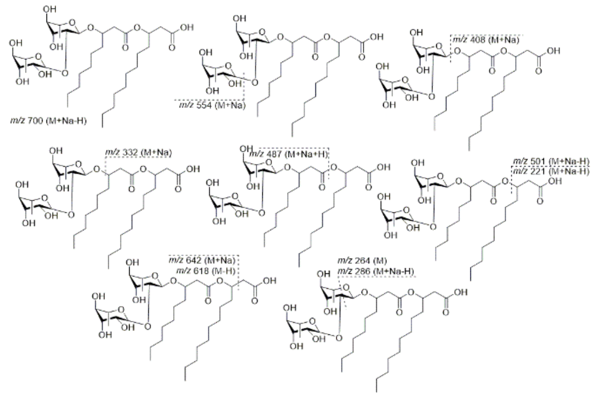
m/z 700 (M+Na-H)
m/z 554 (M+Na)
m/z 408 (M+Na)
m/z 332 (M+Na)
m/z 487 (M+Na+H)
m/z 501 (M+Na-H)
m/z 221 (M+Na-H)
m/z 642 (M+Na)
m/z 618 (M-H)
m/z 264 (M)
m/z 286 (M+Na-H)

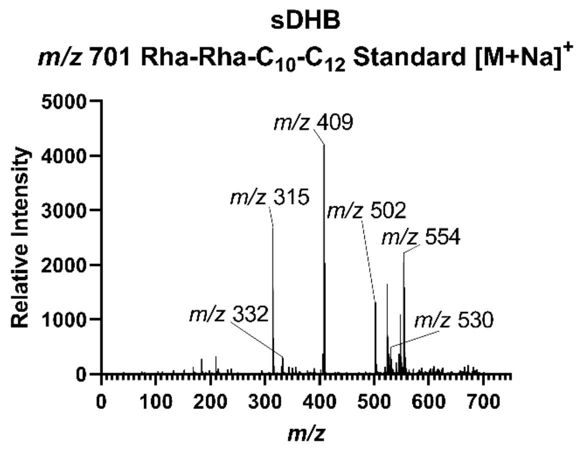
sDHB
m/z 701 Rha-Rha-C10-C12 Standard [M+Na]+
Relative Intensity
m/z
m/z 409
m/z 315
m/z 502
m/z 554
m/z 332
m/z 530
5000
4000
3000
2000
1000
0
0 100 200 300 400 500 600 700

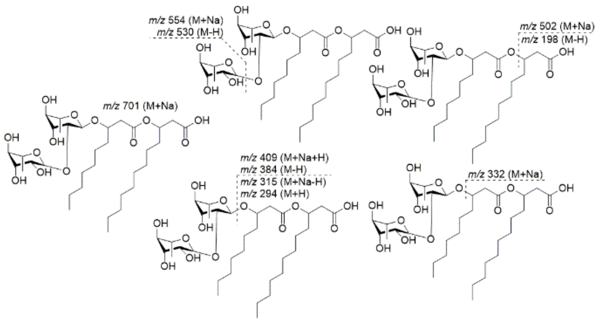
m/z 554 (M+Na)
m/z 530 (M-H)
m/z 502 (M+Na)
m/z 198 (M-H)
m/z 701 (M+Na)
m/z 409 (M+Na+H)
m/z 384 (M-H)
m/z 315 (M+Na-H)
m/z 294 (M+H)
m/z 332 (M+Na)

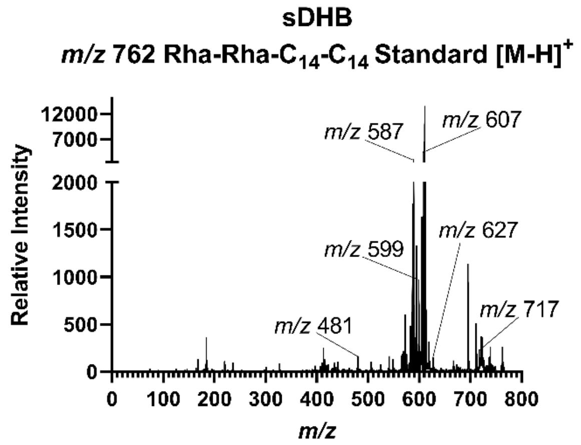
sDHB
m/z 762 Rha-Rha-C14-C14 Standard [M-H]+
Relative Intensity
m/z
m/z 587
m/z 607
m/z 627
m/z 599
m/z 481
m/z 717
12000
7000
2000
1500
1000
500
0
0 100 200 300 400 500 600 700 800

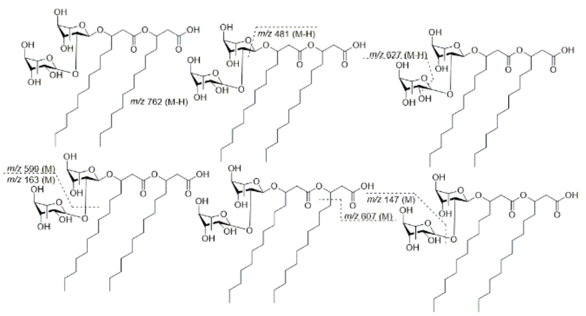
m/z 481 (M-H)
m/z 627 (M-H)
m/z 762 (M-H)
m/z 163 (M)
m/z 147 (M)
m/z 607 (M)

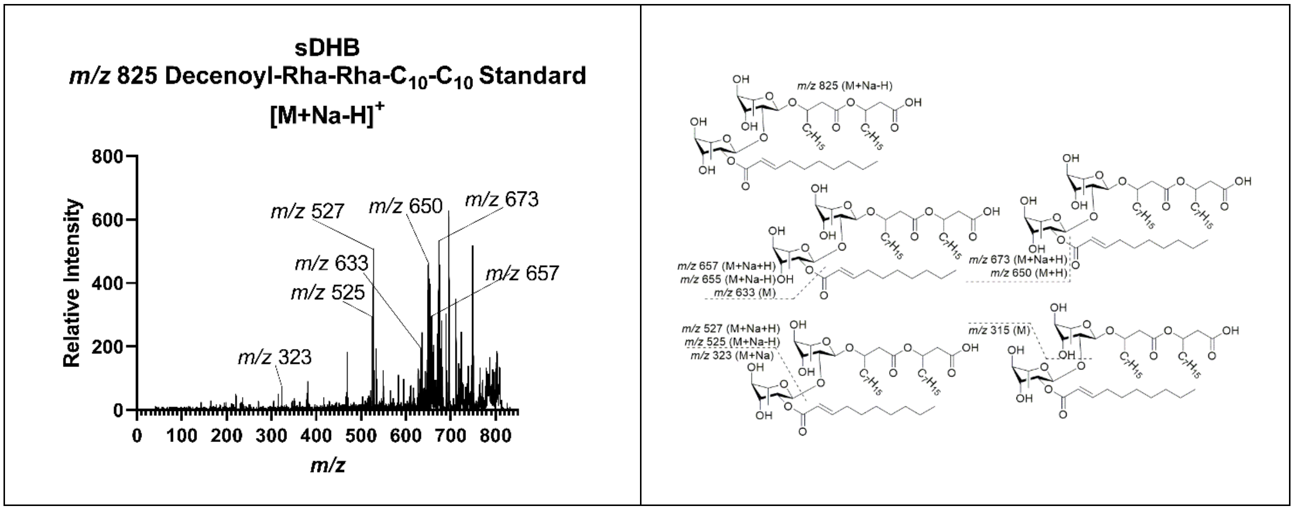
sDHB
m/z 825 Decenoyl-Rha-Rha-C10-C10 Standard
[M+Na-H]+
Relative Intensity
m/z
m/z 527
m/z 650
m/z 673
m/z 633
m/z 525
m/z 657
m/z 323
m/z 825 (M+Na-H)
m/z 657 (M+Na+H)
m/z 655 (M+Na-H)
m/z 633 (M)
m/z 673 (M+Na+H)
m/z 650 (M+H)
m/z 527 (M+Na+H)
m/z 525 (M+Na-H)
m/z 323 (M+Na)
m/z 315 (M)

Table S6. **Adducts of identified molecules.** Table of all identified molecules and adducts forms.
* Indicates molecule adduct was identified as part of a mixture.

NAME	MW	sDHB	CHCA	CMBZT	HPA	THAP	9AA
**QUINOLONE**							
C7 PQS	259.16			*m/z* 304.44 M+2Na-H*			
C7:1 PQS	257.15					*m/z* 257.60 M	
C8 PQS	273.19				*m/z* 297.03 M+Na+H		
C8:1 PQS	271.16					*m/z* 294.68 M+Na	
C9 PQS	287.20	*m/z* 310.22 M+Na		*m/z* 332.51 M+2Na-H		*m/z* 310.79 M+Na	
C11 PQS	315.23	*m/z* 338.23 M+Na *m/z* 360.44 M+2Na-H	*m/z* 338.69 M+Na	*m/z* 338.30 M+Na	*m/z* 339.09 M+Na+H	*m/z* 338.85 M+Na	
C11:2 PQS	311.43					*m/z* 357.65 M+2Na	
C7 AQNO	259.35			*m/z* 304.44 M+2Na-H*			
C8:1 AQNO	271.31			*m/z* 272.26 M+H			
C9 AQNO	287.40			*m/z* 310.00 M+Na			
C11:1 AQNO	313.21	*m/z* 314.28 M+H					
C11:2 AQNO	311.43		*m/z* 357.64 M+2Na		*m/z* 356.19 M+2Na-H		
C12:1 AQNO	327.23						*m/z* 372.27 M+2Na-H
C5 AHQ	215.14		*m/z* 260.53 M+2Na-H				
C7 AHQ	243.17		*m/z* 288.63 M+2Na-H	*m/z* 288.29 M+2Na-H			
C7:1 AHQ	241.15		*m/z* 263.62 M+Na-H				
C8:1 AHQ	255.17			*m/z* 277.09 M+Na-H			
C9 AHQ	271.41		*m/z* 272.62 M+H*				
C10:1 AHQ	283.20		*m/z* 284.74 M+H				
C11:1 AHQ	297.21					*m/z* 298.77 M+H	
C12:1 AHQ	311.23					*m/z* 335.71 M+Na+H	
3-Hydroxy-3-nonyl-1H-quinoline-2,4-dione	303.40			*m/z* 304.44 M+H*		*m/z* 325.75 M+Na-H	
**NAME**	**MW**	**sDHB**	**CHCA**	**CMBZT**	**HPA**	**THAP**	**9AA**
**PHOSPHOLIPID**							
LPA (16:1)	407.22		*m/z* 452.97 M+2Na				
LPE (17:0)	467.30		*m/z* 489.00 M+Na-H				
LPG (18:0)	511.61				*m/z* 556.23 M+2Na-H		
LPG (18:1)	509.29		*m/z* 554.17 M+2Na-H*		*m/z* 532.23 M+Na *m/z* 554.24 M+2Na-H		

NAME	MW	sDHB	CHCA	CMBZT	HPA	THAP	9AA
**PHOSPHOLIPID**							
PA (16:0/15:1)	631.43				*m/z* 677.37 M+2Na		
PA (18:0/16:0)	675.50		*m/z* 698.00 M+Na				
PA (18:0/17:0)	689.51	*m/z* 712.72 M+Na					
PA (18:1/16:0)	673.48	*m/z* 696.72 M+Na *m/z* 697.67 M+Na+H					
PE (14:1/14:0)	633.44		*m/z* 656.94 M+Na+H				
PE (16:0/16:0)	691.51				*m/z* 715.36 M+Na+H		
PE (16:1/16:0)	689.50			*m/z* 734.68 M+2Na-H			
PE (17:2/16:0)	701.50					*m/z* 702.85 M+H	
PE (18:0/16:0)	719.55					*m/z* 742.06 M+Na	
PE (18:0/17:0)	733.56		*m/z* 779.58 M+2Na				
PE (18:1/16:0)	717.53	*m/z* 740.92 M+Na *m/z* 762.91 M+2Na	*m/z* 763.58 M+2Na	*m/z* 762.96 M+2Na		*m/z* 741.24 M+Na+H *m/z* 763.29 M+2Na	*m/z* 762.89 M+2Na
PE (18:1/16:1)	715.52						*m/z* 760.96 M+2Na
PE (18:1/17:1)	729.53	*m/z* 776.88 M+2Na+H					
PE (18:2/16:0)	715.52	*m/z* 761.00 M+2Na		*m/z* 761.03 M+2Na		*m/z* 762.04 M+2Na+H	
PE (18:2/16:1)	713.50					*m/z* 758.03 M+2Na-H*	
PE (19:1/16:0)	731.55	*m/z* 754.89 M+Na					
PE (20:0/19:0)	789.62		*m/z* 813.19 M+Na				
PG (16:1/15:1)	703.92				*m/z* 749.33 M+2Na		
PG (18:0/17:0)	763.55	*m/z* 809.92 M+2Na					
PG (18:1/16:0)	747.52	*m/z* 792.87 M+2Na-H					
PG (18:1/17:0)	761.53					*m/z* 784.13 M+Na	
PG (18:1/17:1)	760.02	*m/z* 782.99 M+Na		*m/z* 783.08 M+Na			
PG (18:1/18:1)	774.05	*m/z* 798.98 M+Na+H *m/z* 820.92 M+2Na+H					
PG (19:0/16:0)	763.55	*m/z* 787.93 M+Na+H					
PG (19:1/19:0)	803.58					*m/z* 827.05 M+Na+H	
**NAME**	**MW**	**sDHB**	**CHCA**	**CMBZT**	**HPA**	**THAP**	**9AA**
**RHAMNOLIPID**							
Rha-C_{10}	334.41		*m/z* 379.68 M+2Na-H				

NAME	MW	sDHB	CHCA	CMBZT	HPA	THAP	9AA
**RHAMNOLIPID**							
Rha-$C_{12:1}$	360.45				*m/z* 361.10 M+H		
Rha-$C_{12:2}$	358.43			*m/z* 403.06 M+2Na-H		*m/z* 359.85 M+H *m/z* 403.87 M+2Na-H	
Rha-C_{14}	390.52					*m/z* 390.85 M	
Rha-C_8-C_8	448.55				*m/z* 472.23 M+Na+H		
Rha-C_8-C_{12}	504.65					*m/z* 528.40 M+Na+H	
Rha-C_{10}-C_8	476.6				*m/z* 500.26 M+Na+H		
Rha-C_{10}-C_{12}	532.71		*m/z* 554.17 M+Na-H*				
Rha-C_{12}-C_{14}	588.81				*m/z* 633.36 M+2Na-H		
Rha-$C_{12:1}$-C_{10}	530.69	*m/z* 575.62 M+2Na-H					
Rha-C_{14}-C_8	532.72	*m/z* 577.82 M+2Na-H					
Rha-$C_{14:1}$-C_8	530.70			*m/z* 575.68 M+2Na-H			
Rha-C_{14}-C_{10}	560.76		*m/z* 605.24 M+2Na-H				
Rha-C_{14}-C_{14}	616.87			*m/z* 617.81 M+H			
Rha-C_{16}-C_{16}	672.99				*m/z* 695.35 M+Na		
Rha-Rha-C_8	452.49						*m/z* 474.18 M+Na-H *m/z* 476.17 M+Na+H
Rha-Rha-C_{10}-C_{10}	650.79	*m/z* 674.73 M+Na+H				*m/z* 674.75 M+Na+H	
Rha-Rha-$C_{12:1}$-C_8	648.78				*m/z* 671.36 M+Na		
Rha-Rha-C_{12}-C_{14}	734.95					*m/z* 758.03 M+Na+H*	
Rha-Rha-C_{14}-C_{12}	734.95	*m/z* 756.90 M+Na-H				*m/z* 779.28 M+2Na-H	
Decenoyl-Rha-C_{10}-C_{10}	656.89	*m/z* 657.64 M+H					
Decenoyl-Rha-Rha-C_{10}-C_{10}	803.03	*m/z* 827.83 M+Na+H			*m/z* 848.45 M+2Na-H		
**NAME**	**MW**	**sDHB**	**CHCA**	**CMBZT**	**HPA**	**THAP**	**9AA**
**OTHER**							
3-OH-C4-AHL	187.20		*m/z* 211.40 M+Na+H				
3-Hydroxy-hexanoyl-L-homoserine lactone	215.25			*m/z* 260.22 M+2Na-H			
Phosphoenolpyruvate	168.04		*m/z* 190.46 M+Na-H				
Palmitic acid C16	256.43				*m/z* 301.09 M+2Na-H		
Kynurenine	208.22		*m/z* 230.61 M+Na-H				
Arginine	174.20		*m/z* 219.54 M+2Na-H				
Arogenic acid	227.22		*m/z* 272.62 M+2Na-H*				

Table S7. Identified ions of interest spectra and fragments. All collected spectra and fragments required for identification.

<table>
<tr><th>Spectrum</th><th>Structure Fragments</th></tr>
<tr><td>
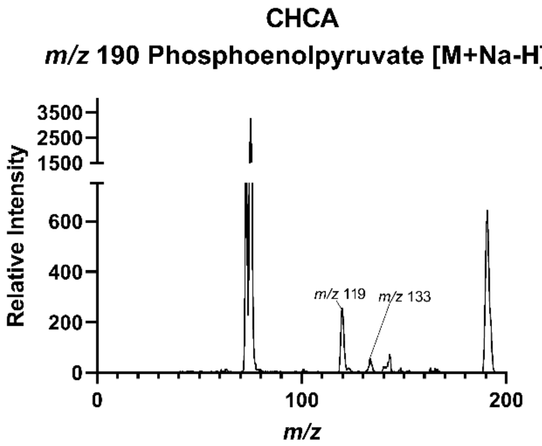

</td><td>
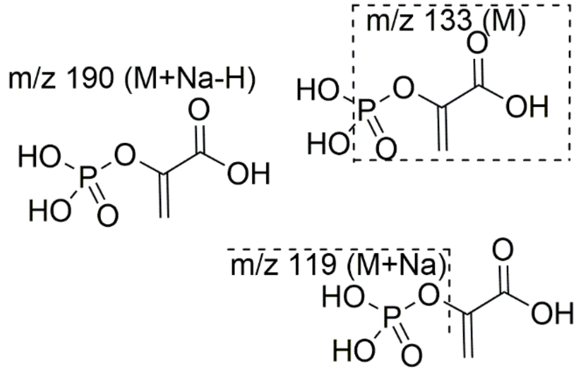

</td></tr>
<tr><td>
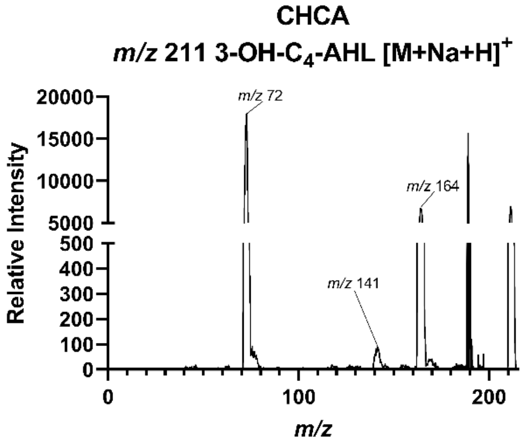

</td><td>
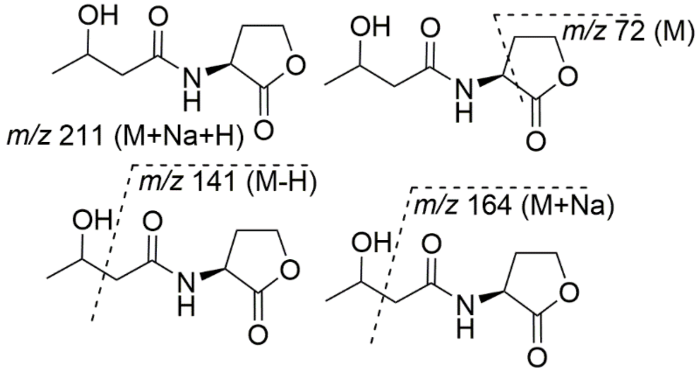

</td></tr>
<tr><td>
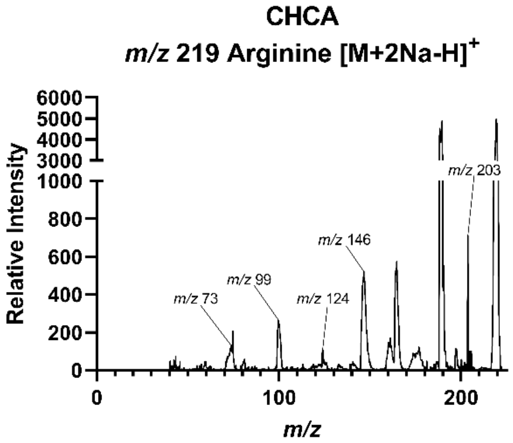

</td><td>
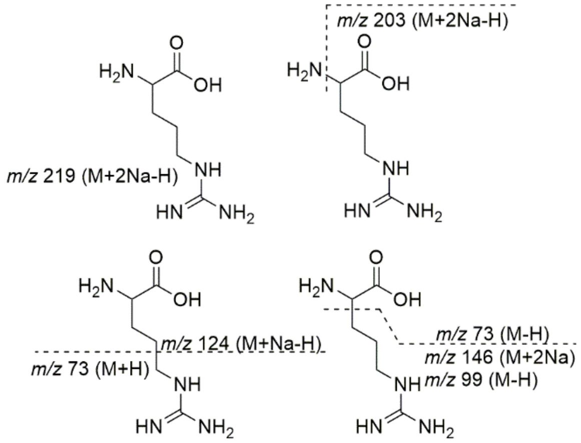

</td></tr>
</table>

CHCA
***m/z* 230 Kynurenine [M+Na-H]$^+$**

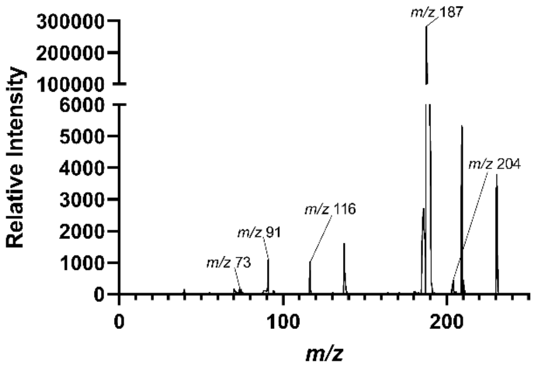

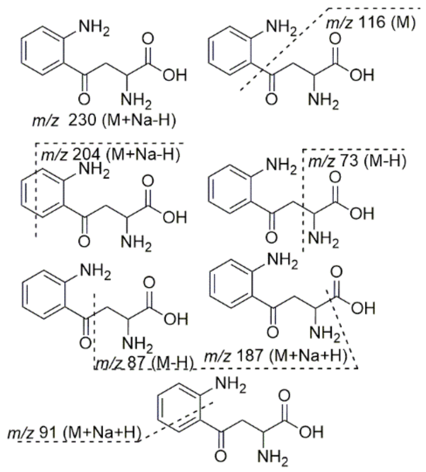

THAP
***m/z* 257 C7:1 PQS [M]$^+$**

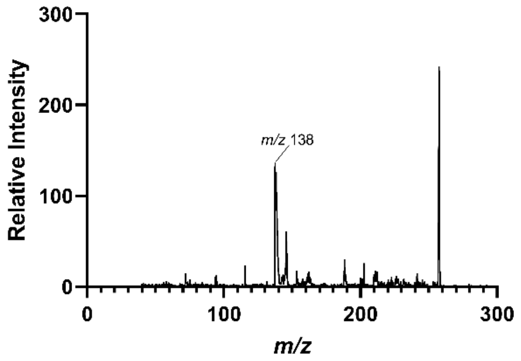

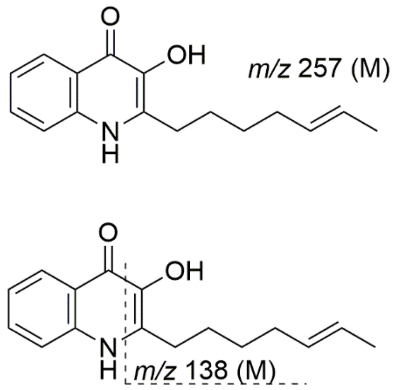

CHCA
***m/z* 260 C5 AHQ [M+2Na-H]$^+$**

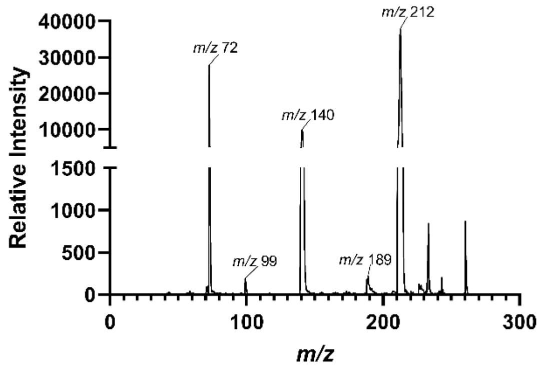

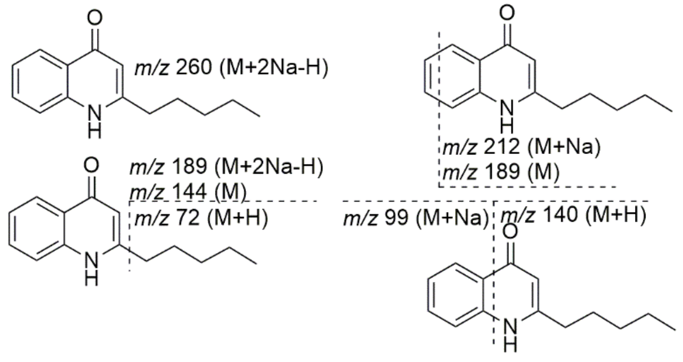

CMBZT
***m/z* 260 3-Hydroxy-hexanoyl-L-homoserine lactone**
[M+2Na-H]$^+$

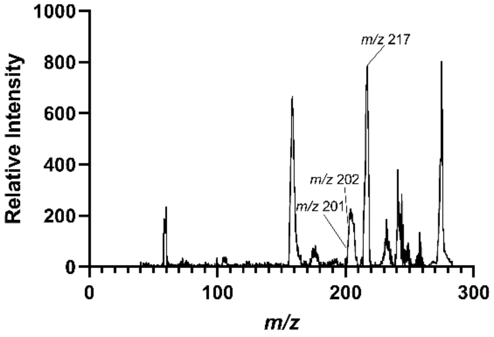

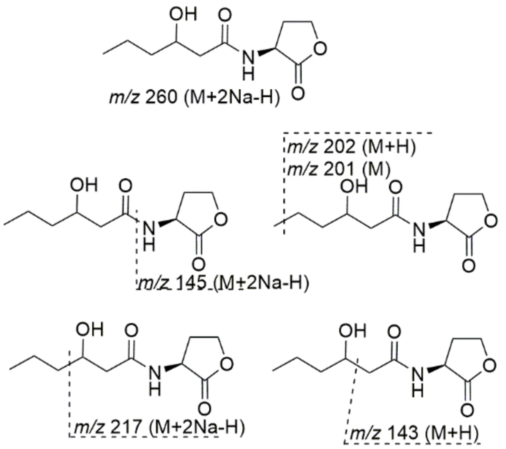

CHCA
***m/z* 263 C7:1 AHQ [M+Na-H]$^+$**

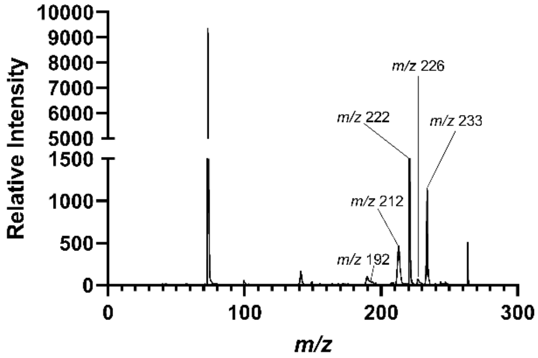

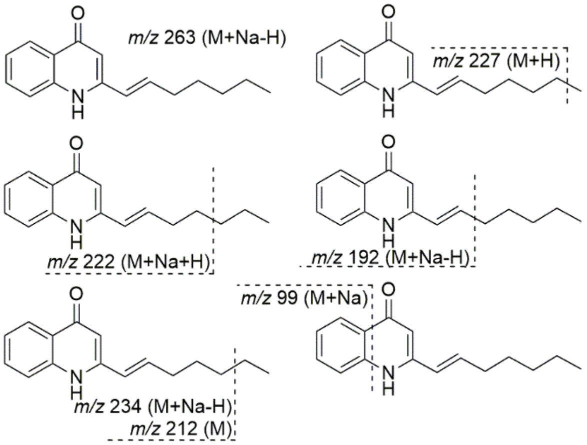

CMBZT
***m/z* 272 C8:1 AQNO [M+H]$^+$**

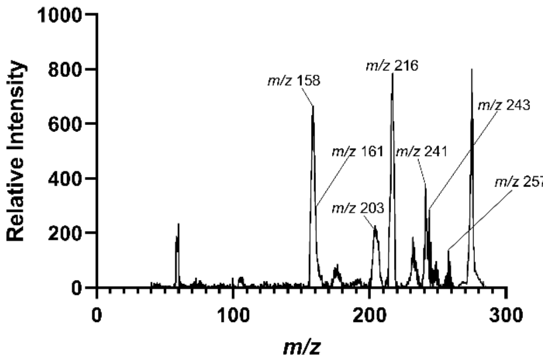

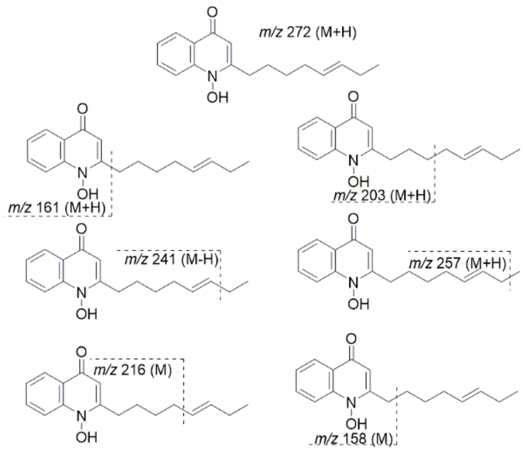

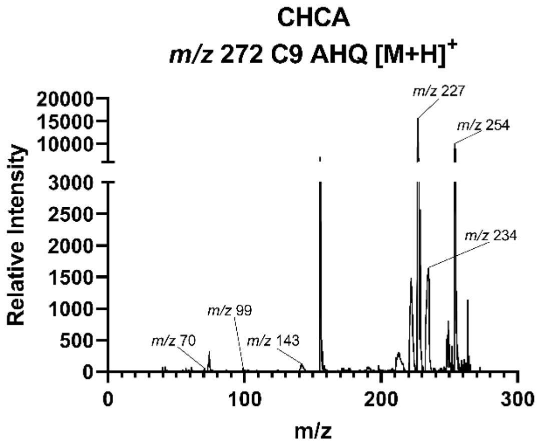
CHCA
m/z 272 C9 AHQ [M+H]+
Relative Intensity
m/z
m/z 227
m/z 254
m/z 234
m/z 70
m/z 99
m/z 143
20000
15000
10000
3000
2500
2000
1500
1000
500
0
100
200
300

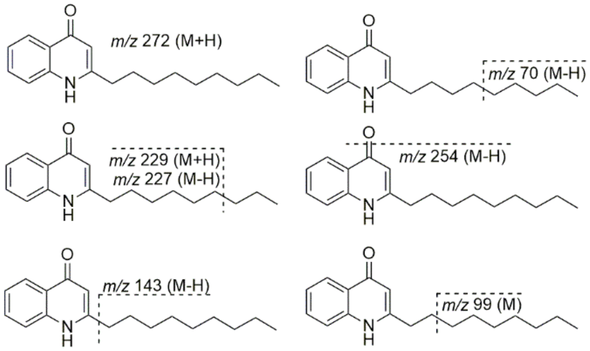
m/z 272 (M+H)
m/z 70 (M-H)
m/z 229 (M+H)
m/z 227 (M-H)
m/z 254 (M-H)
m/z 143 (M-H)
m/z 99 (M)

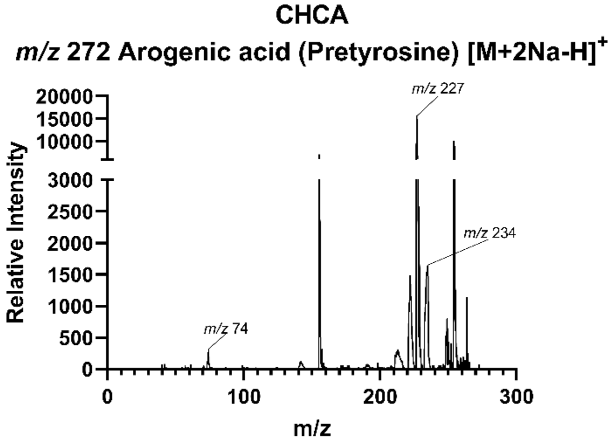
CHCA
m/z 272 Arogenic acid (Pretyrosine) [M+2Na-H]+
Relative Intensity
m/z
m/z 227
m/z 234
m/z 74
20000
15000
10000
3000
2500
2000
1500
1000
500
0
100
200
300

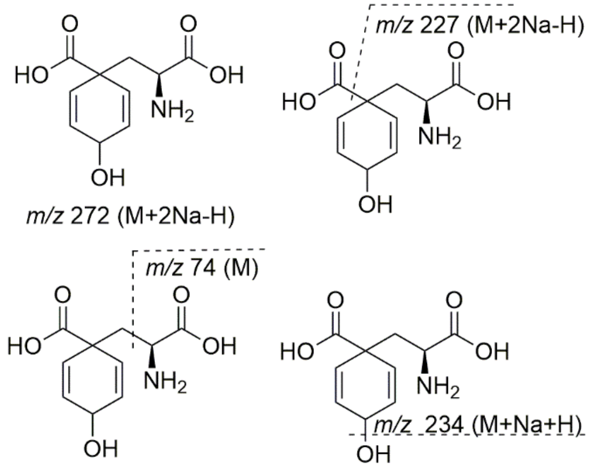
m/z 272 (M+2Na-H)
m/z 227 (M+2Na-H)
m/z 74 (M)
m/z 234 (M+Na+H)

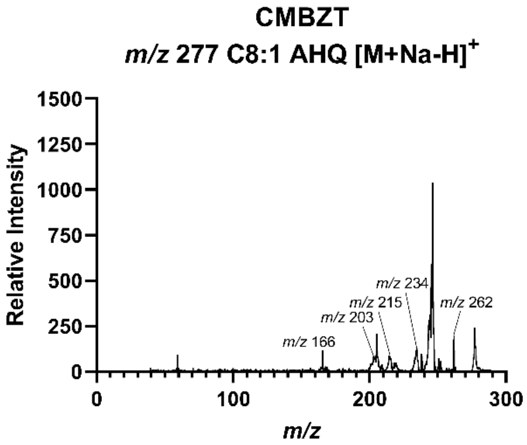
CMBZT
m/z 277 C8:1 AHQ [M+Na-H]+
Relative Intensity
m/z
m/z 166
m/z 203
m/z 215
m/z 234
m/z 262
1500
1250
1000
750
500
250
0
100
200
300

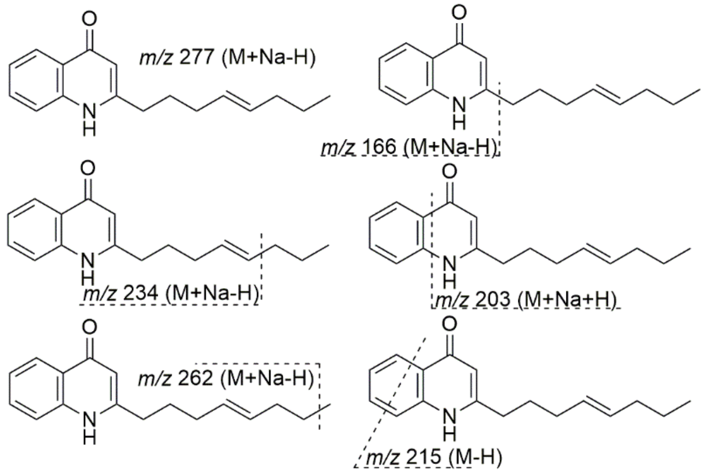
m/z 277 (M+Na-H)
m/z 166 (M+Na-H)
m/z 234 (M+Na-H)
m/z 203 (M+Na+H)
m/z 262 (M+Na-H)
m/z 215 (M-H)

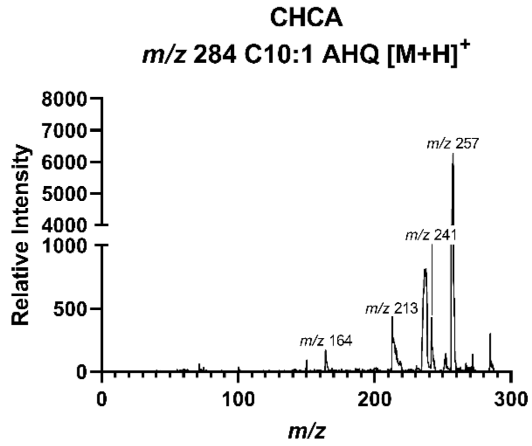
CHCA
m/z 284 C10:1 AHQ [M+H]+
Relative Intensity
m/z
m/z 257
m/z 241
m/z 213
m/z 164

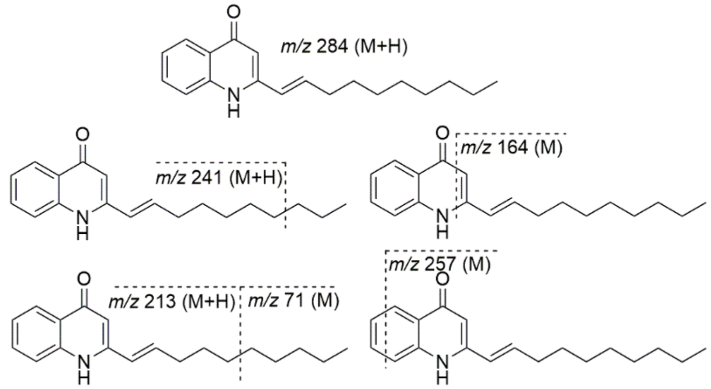
m/z 284 (M+H)
m/z 241 (M+H)
m/z 164 (M)
m/z 257 (M)
m/z 213 (M+H)
m/z 71 (M)

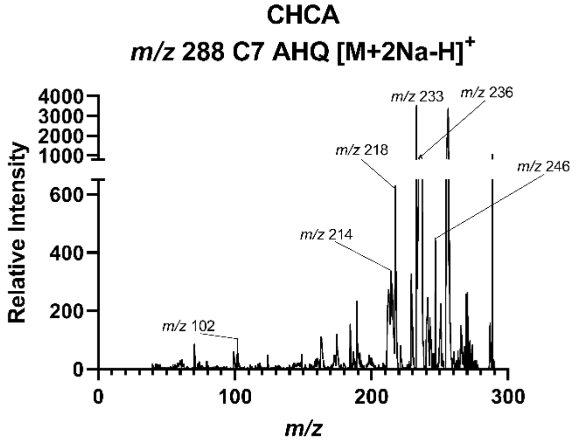
CHCA
m/z 288 C7 AHQ [M+2Na-H]+
Relative Intensity
m/z
m/z 233
m/z 236
m/z 218
m/z 246
m/z 214
m/z 102

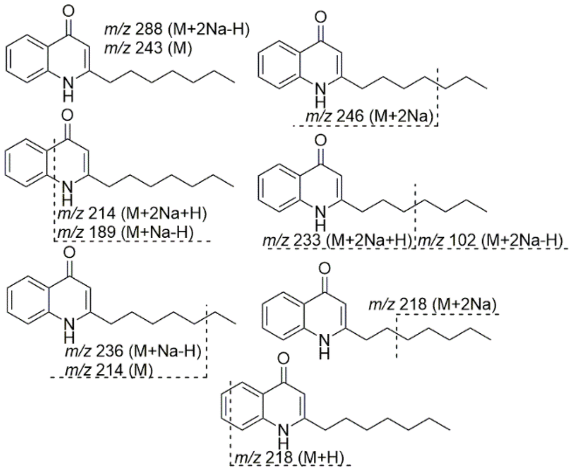
m/z 288 (M+2Na-H)
m/z 243 (M)
m/z 246 (M+2Na)
m/z 214 (M+2Na+H)
m/z 189 (M+Na-H)
m/z 233 (M+2Na+H)
m/z 102 (M+2Na-H)
m/z 236 (M+Na-H)
m/z 214 (M)
m/z 218 (M+2Na)
m/z 218 (M+H)

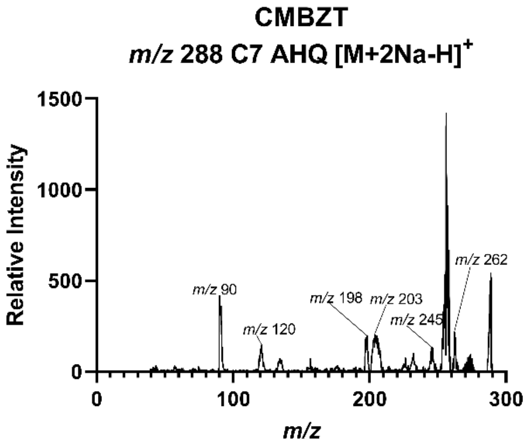
CMBZT
m/z 288 C7 AHQ [M+2Na-H]+
Relative Intensity
m/z
m/z 90
m/z 120
m/z 198
m/z 203
m/z 245
m/z 262

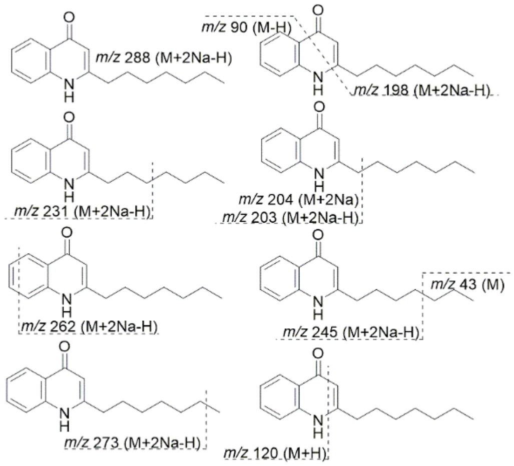
m/z 288 (M+2Na-H)
m/z 90 (M-H)
m/z 198 (M+2Na-H)
m/z 231 (M+2Na-H)
m/z 204 (M+2Na)
m/z 203 (M+2Na-H)
m/z 262 (M+2Na-H)
m/z 43 (M)
m/z 245 (M+2Na-H)
m/z 273 (M+2Na-H)
m/z 120 (M+H)

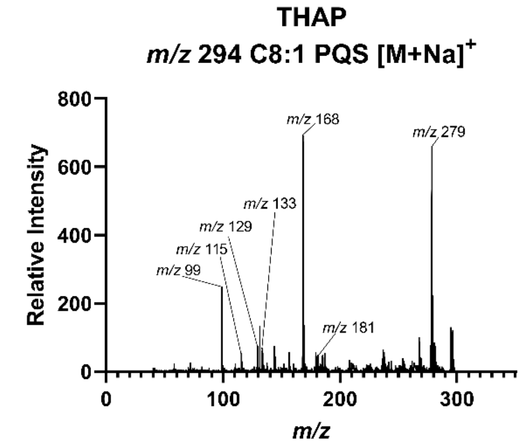
THAP
m/z 294 C8:1 PQS [M+Na]+
Relative Intensity
m/z
m/z 168
m/z 279
m/z 133
m/z 129
m/z 115
m/z 99
m/z 181

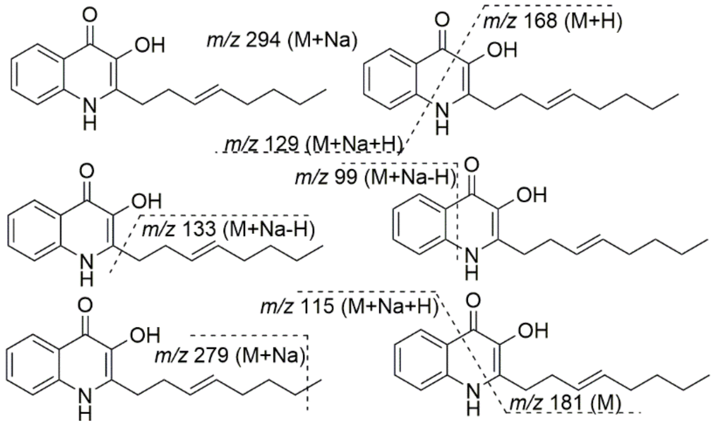
m/z 294 (M+Na)
m/z 168 (M+H)
m/z 129 (M+Na+H)
m/z 99 (M+Na-H)
m/z 133 (M+Na-H)
m/z 115 (M+Na+H)
m/z 279 (M+Na)
m/z 181 (M)

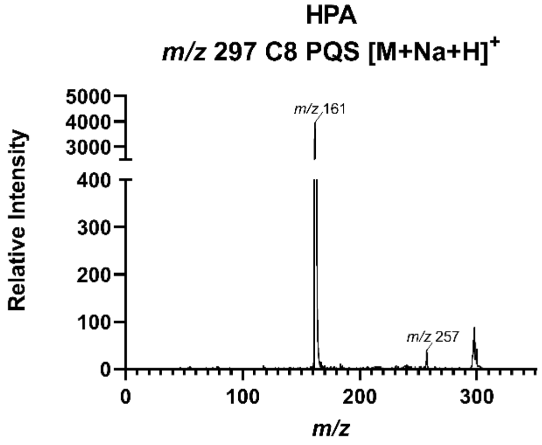
HPA
m/z 297 C8 PQS [M+Na+H]+
Relative Intensity
m/z
m/z 161
m/z 257

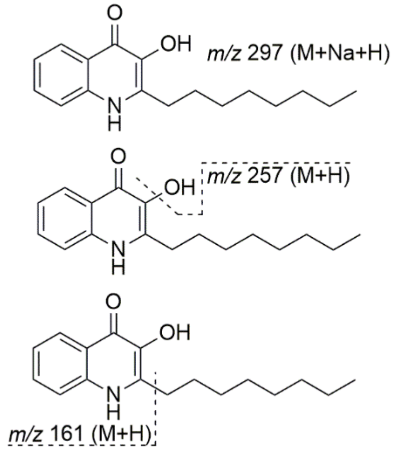
m/z 297 (M+Na+H)
m/z 257 (M+H)
m/z 161 (M+H)

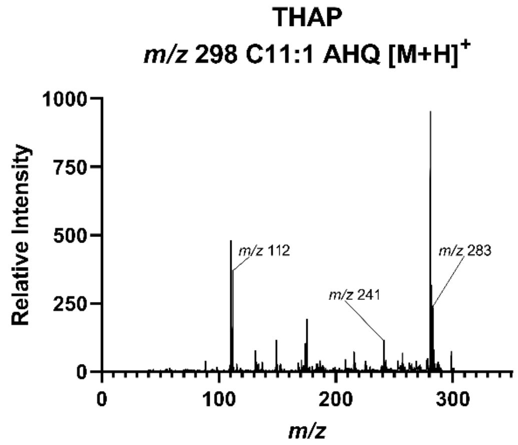
THAP
m/z 298 C11:1 AHQ [M+H]+
Relative Intensity
m/z
m/z 112
m/z 283
m/z 241

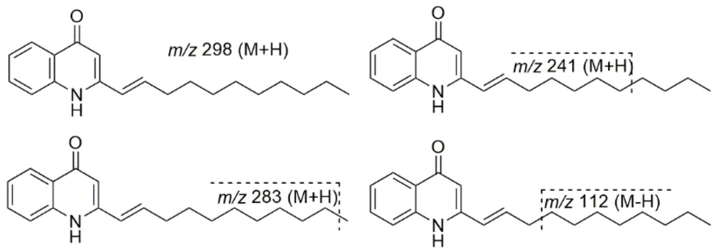
m/z 298 (M+H)
m/z 241 (M+H)
m/z 283 (M+H)
m/z 112 (M-H)

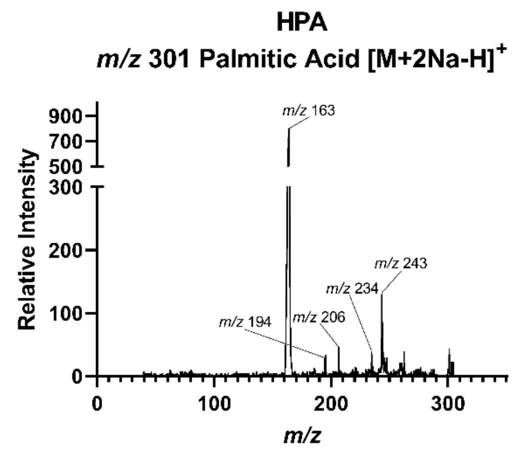
HPA
m/z 301 Palmitic Acid [M+2Na-H]+
Relative Intensity
m/z
m/z 163
m/z 243
m/z 234
m/z 206
m/z 194

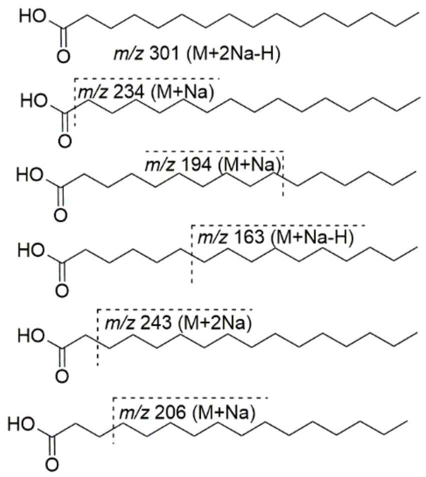
HO
O
m/z 301 (M+2Na-H)
m/z 234 (M+Na)
m/z 194 (M+Na)
m/z 163 (M+Na-H)
m/z 243 (M+2Na)
m/z 206 (M+Na)

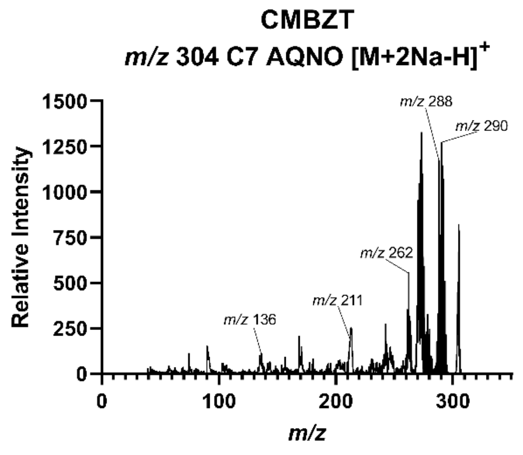
CMBZT
m/z 304 C7 AQNO [M+2Na-H]+
Relative Intensity
m/z
m/z 288
m/z 290
m/z 262
m/z 211
m/z 136

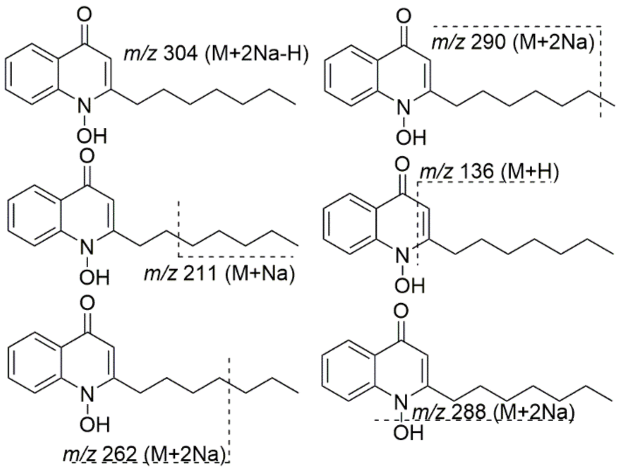
m/z 304 (M+2Na-H)
m/z 290 (M+2Na)
m/z 136 (M+H)
m/z 211 (M+Na)
m/z 288 (M+2Na)
m/z 262 (M+2Na)
N
OH

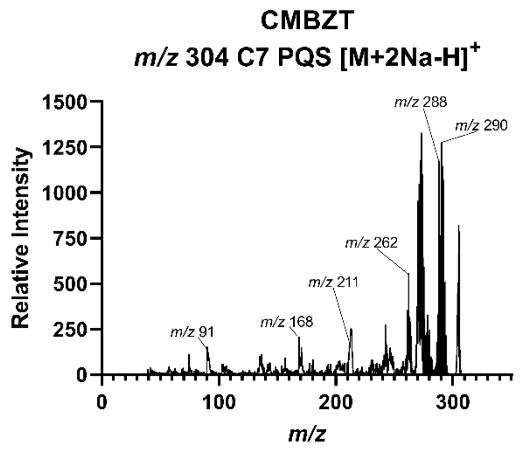
CMBZT
m/z 304 C7 PQS [M+2Na-H]+
Relative Intensity
m/z
m/z 288
m/z 290
m/z 262
m/z 211
m/z 168
m/z 91

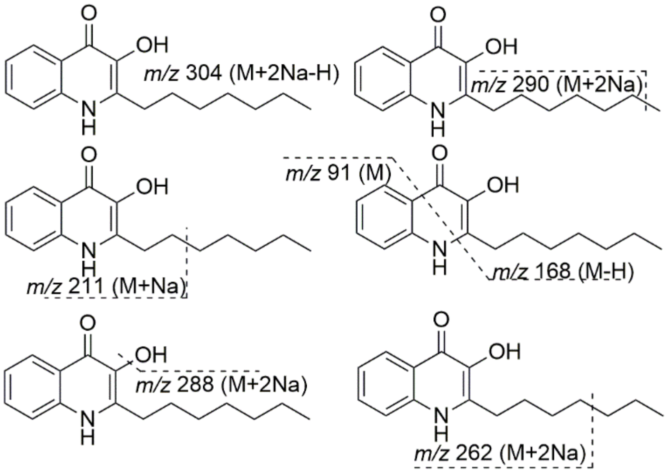
OH
m/z 304 (M+2Na-H)
m/z 290 (M+2Na)
m/z 91 (M)
m/z 211 (M+Na)
m/z 168 (M-H)
m/z 288 (M+2Na)
m/z 262 (M+2Na)
N
H

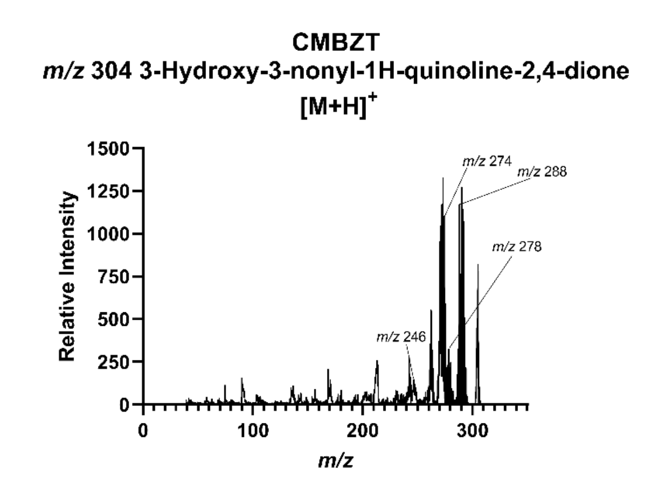
CMBZT
m/z 304 3-Hydroxy-3-nonyl-1H-quinoline-2,4-dione
[M+H]+
Relative Intensity
m/z
m/z 274
m/z 288
m/z 278
m/z 246

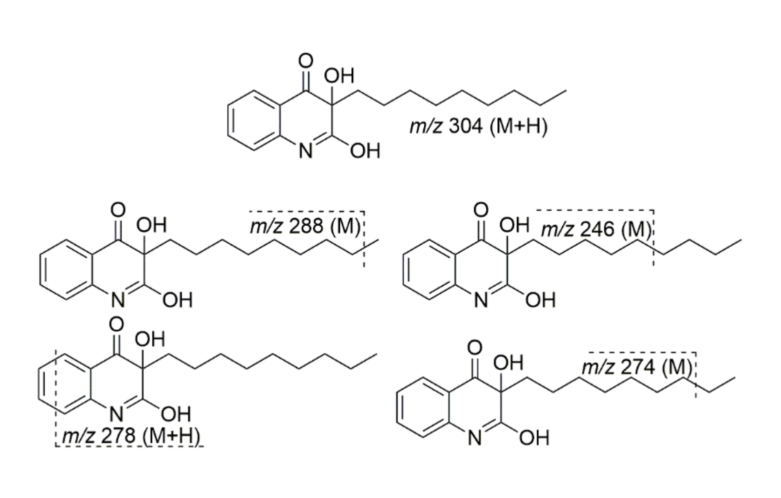
m/z 304 (M+H)
m/z 288 (M)
m/z 246 (M)
m/z 278 (M+H)
m/z 274 (M)

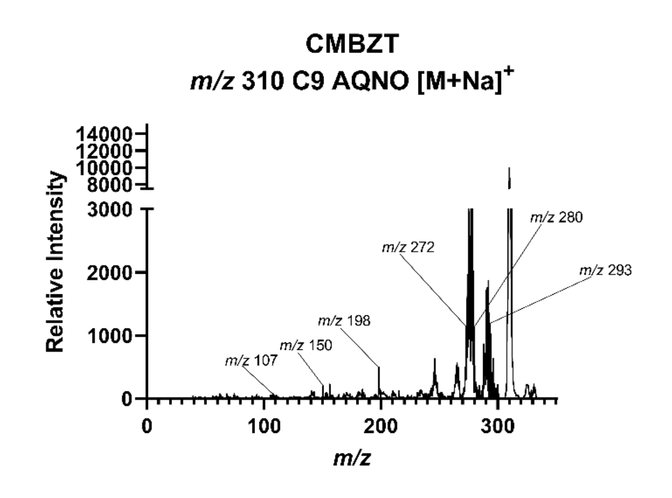
CMBZT
m/z 310 C9 AQNO [M+Na]+
Relative Intensity
m/z
m/z 280
m/z 272
m/z 293
m/z 198
m/z 150
m/z 107

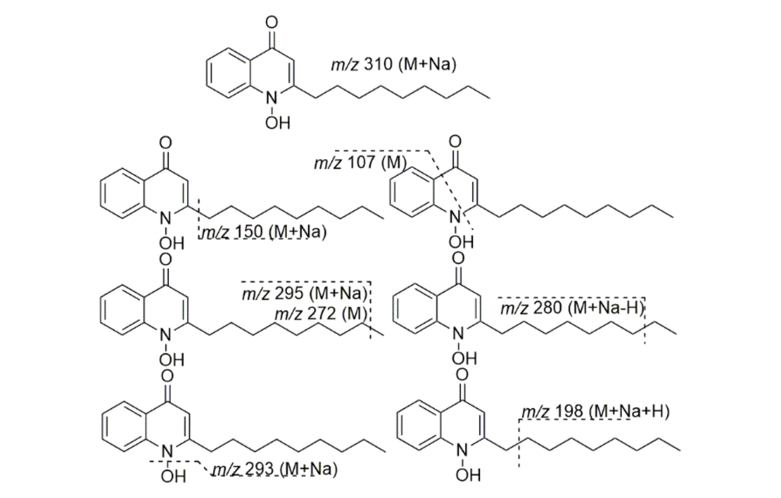
m/z 310 (M+Na)
m/z 107 (M)
m/z 150 (M+Na)
m/z 295 (M+Na)
m/z 272 (M)
m/z 280 (M+Na-H)
m/z 198 (M+Na+H)
m/z 293 (M+Na)

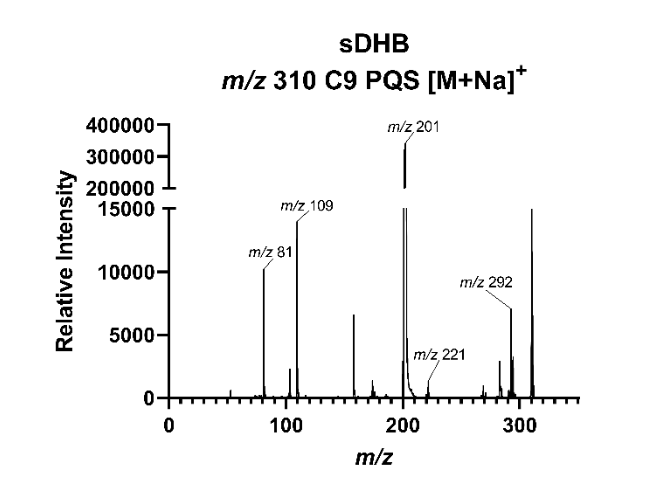
sDHB
m/z 310 C9 PQS [M+Na]+
Relative Intensity
m/z
m/z 201
m/z 109
m/z 81
m/z 292
m/z 221

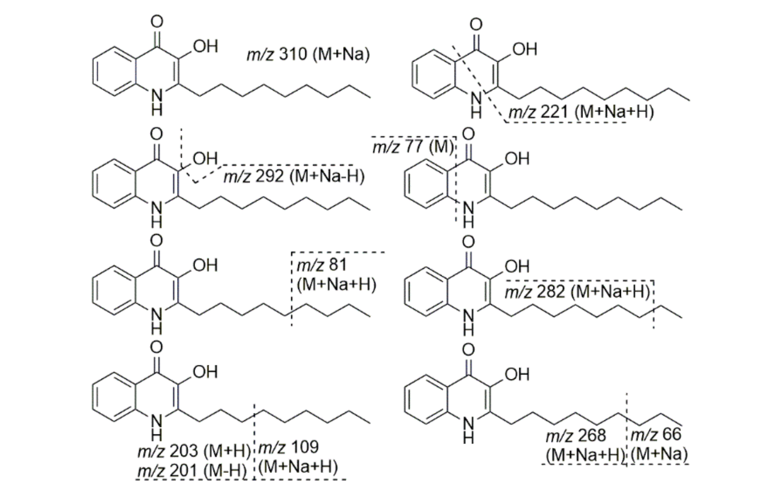
m/z 310 (M+Na)
m/z 221 (M+Na+H)
m/z 292 (M+Na-H)
m/z 77 (M)
m/z 81 (M+Na+H)
m/z 282 (M+Na+H)
m/z 203 (M+H)
m/z 201 (M-H)
m/z 109 (M+Na+H)
m/z 268 (M+Na+H)
m/z 66 (M+Na)

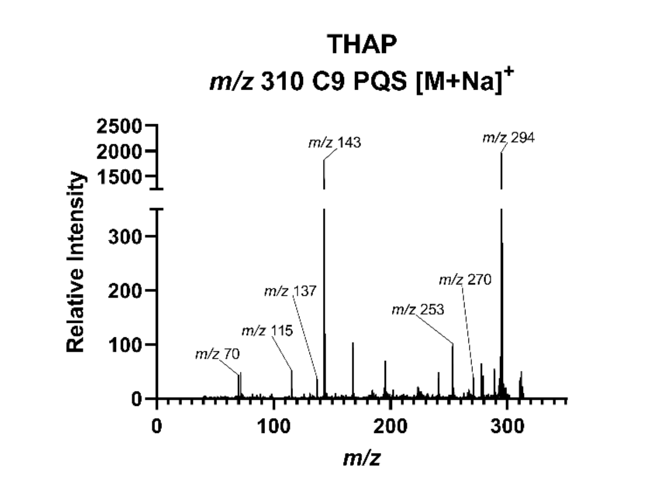
THAP
m/z 310 C9 PQS [M+Na]+
Relative Intensity
m/z
m/z 70
m/z 115
m/z 137
m/z 143
m/z 253
m/z 270
m/z 294

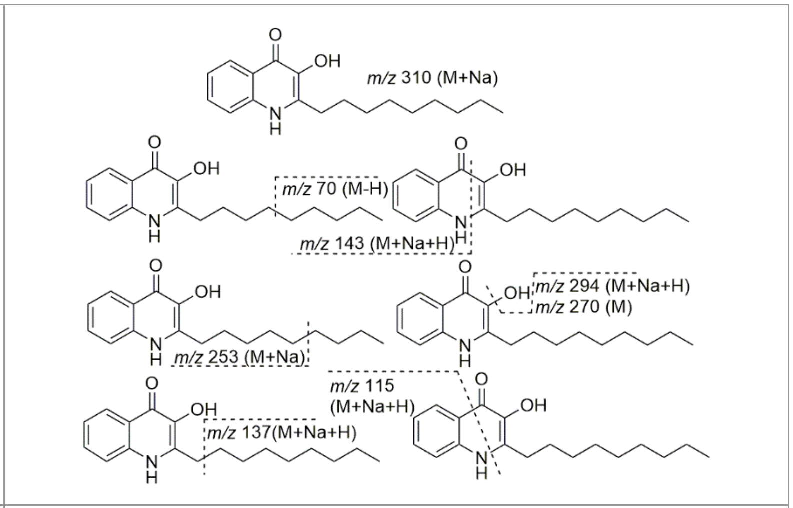
m/z 310 (M+Na)
m/z 70 (M-H)
m/z 143 (M+Na+H)
m/z 253 (M+Na)
m/z 294 (M+Na+H)
m/z 270 (M)
m/z 115 (M+Na+H)
m/z 137(M+Na+H)

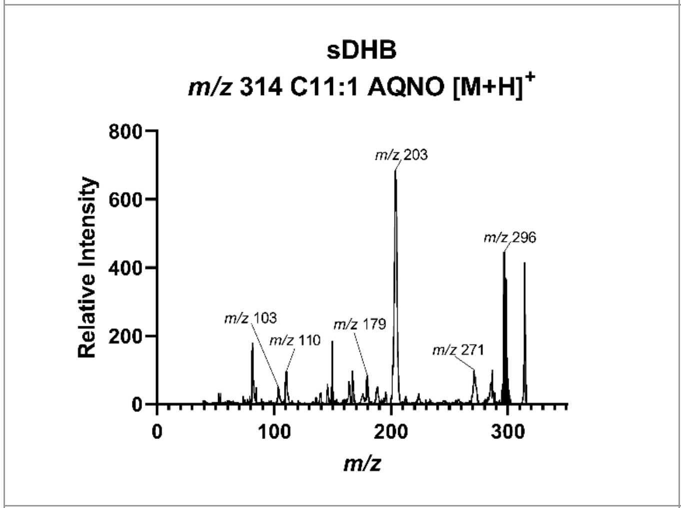
sDHB
m/z 314 C11:1 AQNO [M+H]+
Relative Intensity
m/z
m/z 103
m/z 110
m/z 179
m/z 203
m/z 271
m/z 296

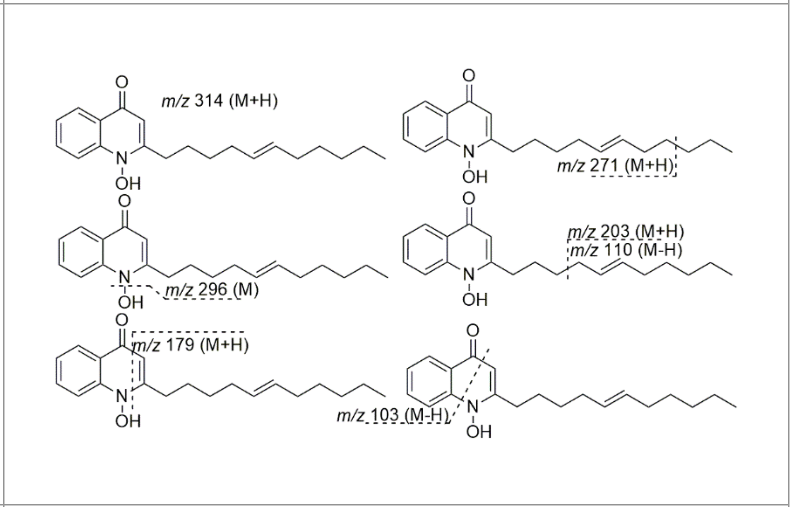
m/z 314 (M+H)
m/z 271 (M+H)
m/z 296 (M)
m/z 203 (M+H)
m/z 110 (M-H)
m/z 179 (M+H)
m/z 103 (M-H)

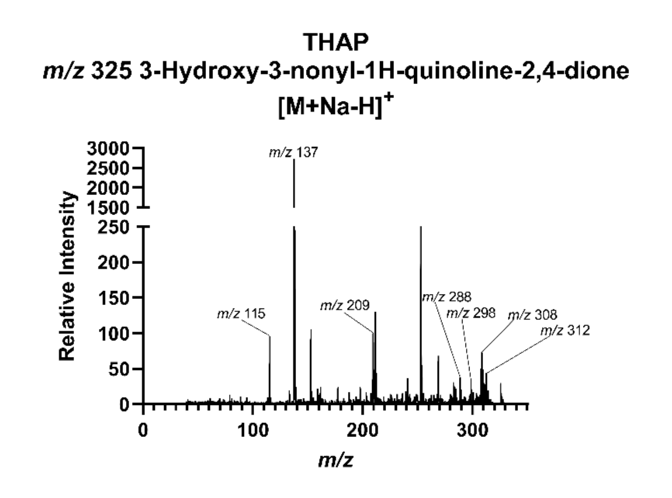
THAP
m/z 325 3-Hydroxy-3-nonyl-1H-quinoline-2,4-dione
[M+Na-H]+
Relative Intensity
m/z
m/z 115
m/z 137
m/z 209
m/z 288
m/z 298
m/z 308
m/z 312

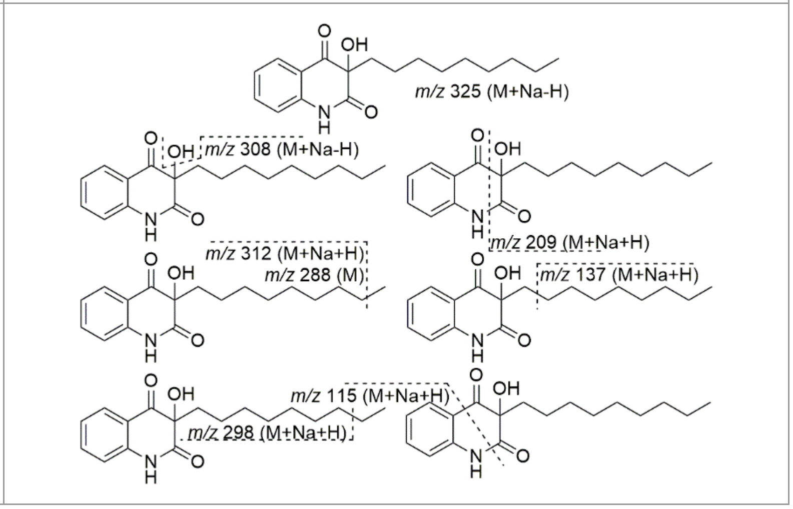
m/z 325 (M+Na-H)
m/z 308 (M+Na-H)
m/z 209 (M+Na+H)
m/z 312 (M+Na+H)
m/z 288 (M)
m/z 137 (M+Na+H)
m/z 115 (M+Na+H)
m/z 298 (M+Na+H)

CMBZT

m/z 332 C9 PQS $[M+2Na-H]^+$

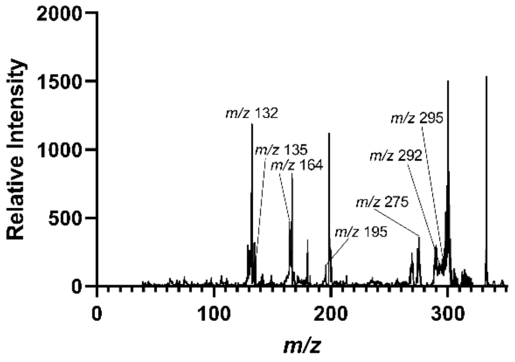

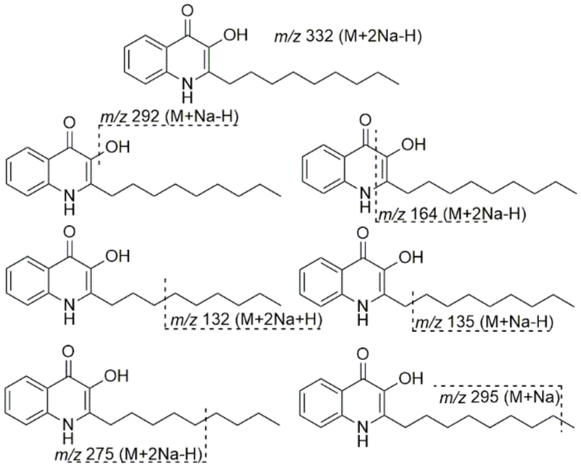

THAP

m/z 335 C12:1 AHQ $[M+Na+H]^+$

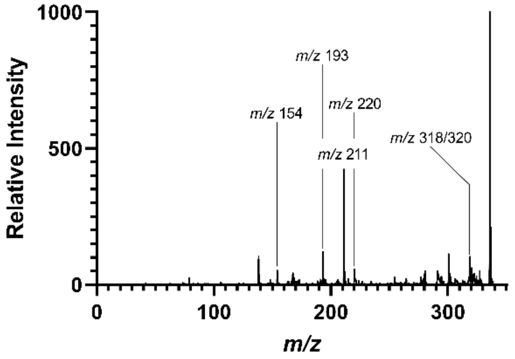

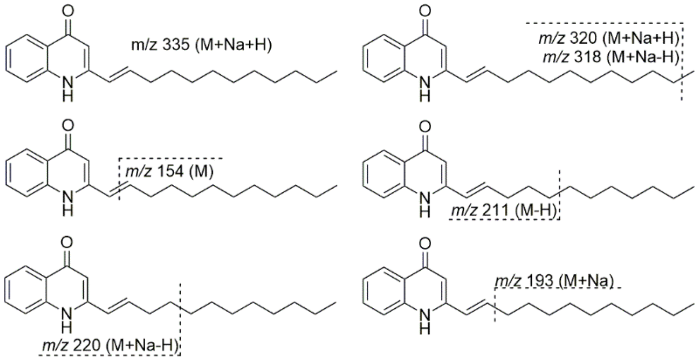

CHCA

m/z 338 C11 PQS $[M+Na]^+$

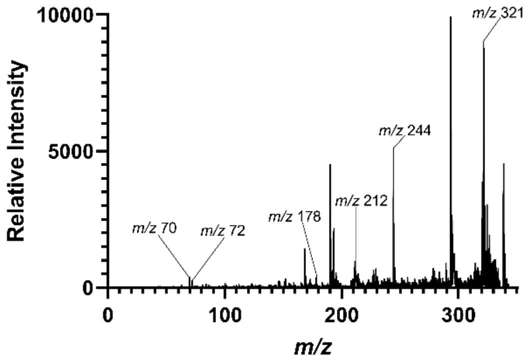

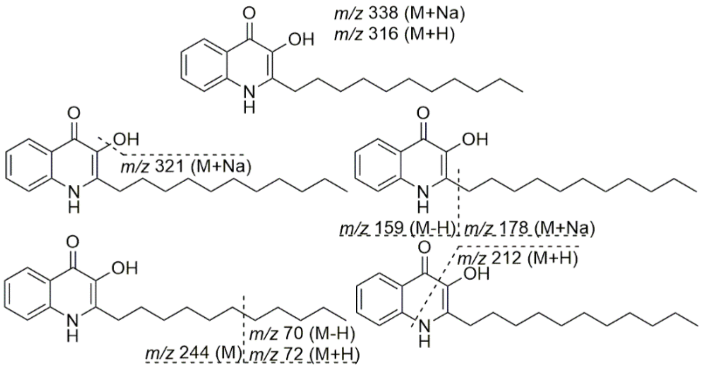

CMBZT
***m/z* 338 C11 PQS [M+Na]⁺**

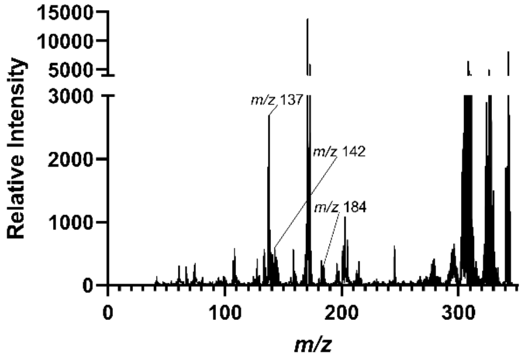

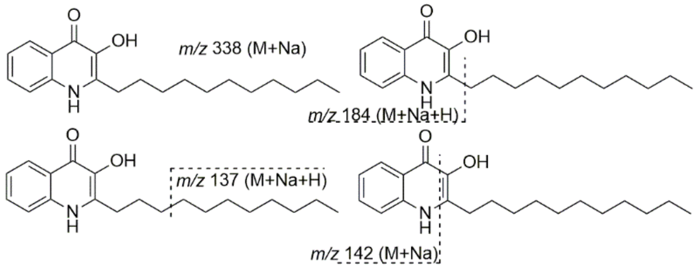

sDHB
***m/z* 338 C11 PQS [M+Na]⁺**

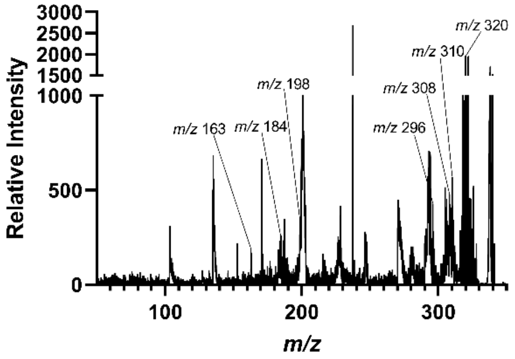

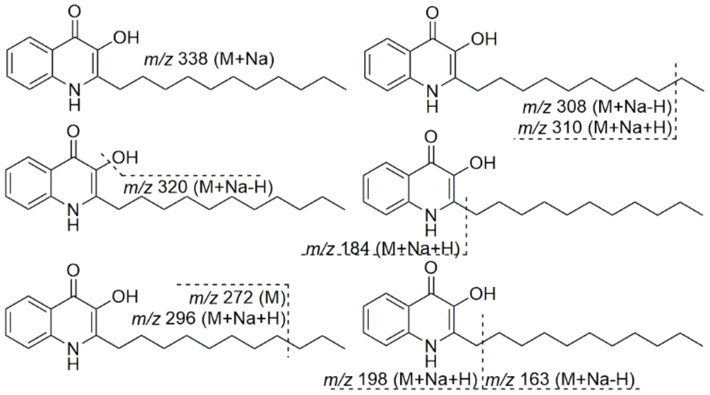

THAP
***m/z* 338 C11 PQS [M+Na]⁺**

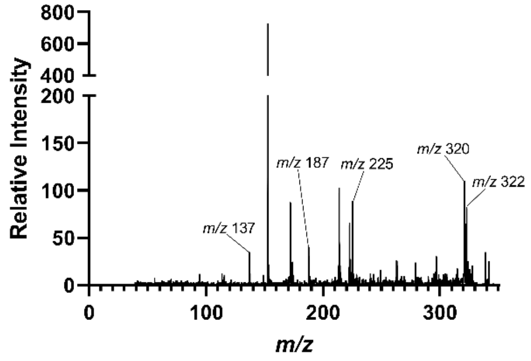

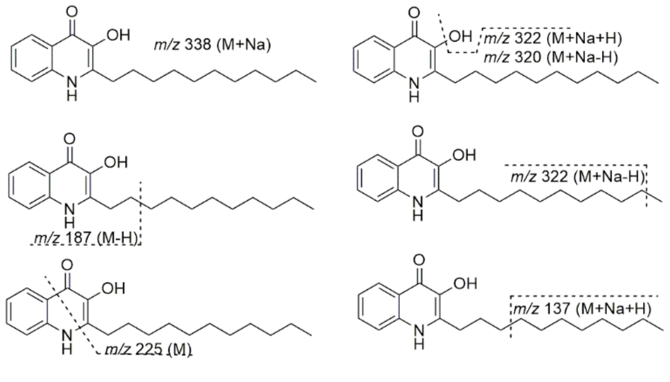

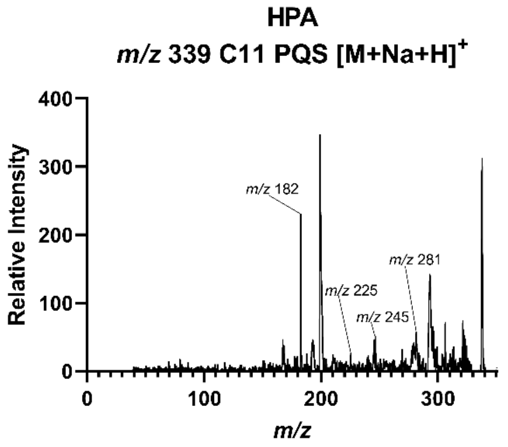
HPA
m/z 339 C11 PQS [M+Na+H]+
Relative Intensity
m/z
m/z 182
m/z 225
m/z 245
m/z 281
0
100
200
300
400

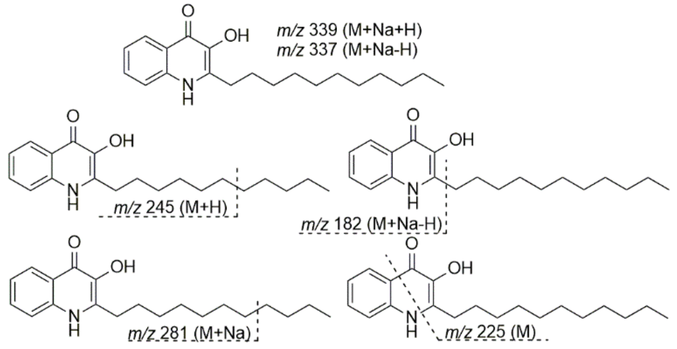
m/z 339 (M+Na+H)
m/z 337 (M+Na-H)
m/z 245 (M+H)
m/z 182 (M+Na-H)
m/z 281 (M+Na)
m/z 225 (M)
O
OH
N
H

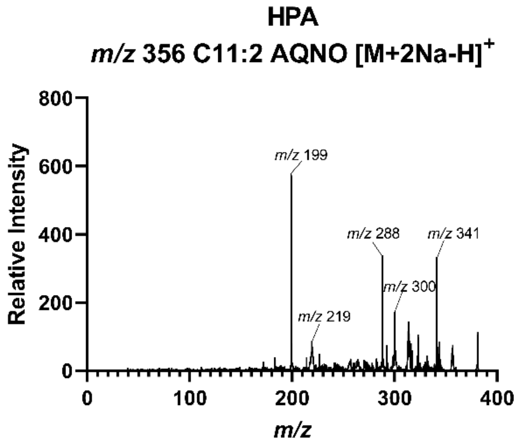
HPA
m/z 356 C11:2 AQNO [M+2Na-H]+
Relative Intensity
m/z
m/z 199
m/z 288
m/z 341
m/z 300
m/z 219
0
100
200
300
400
600
800

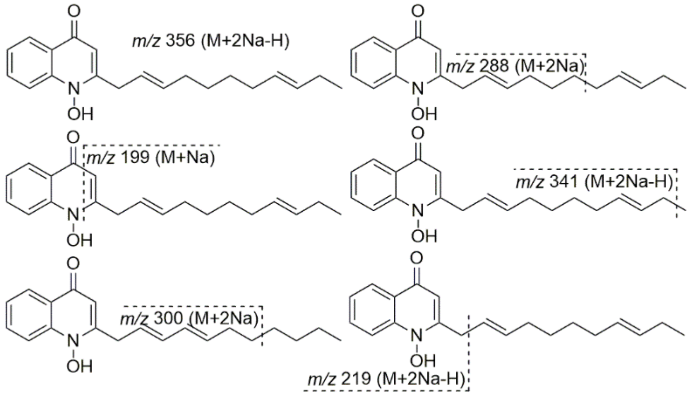
m/z 356 (M+2Na-H)
m/z 288 (M+2Na)
m/z 199 (M+Na)
m/z 341 (M+2Na-H)
m/z 300 (M+2Na)
m/z 219 (M+2Na-H)
O
N
OH

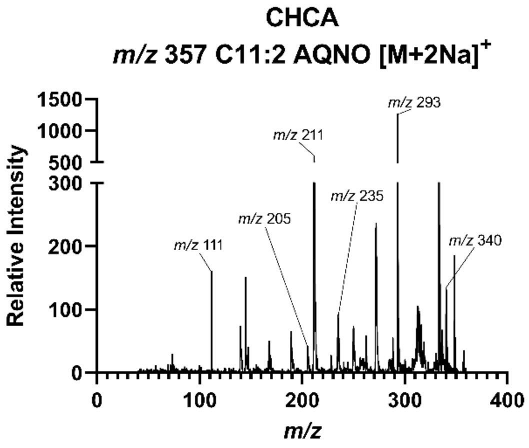
CHCA
m/z 357 C11:2 AQNO [M+2Na]+
Relative Intensity
m/z
m/z 293
m/z 211
m/z 235
m/z 205
m/z 111
m/z 340
0
100
200
300
400
500
1000
1500

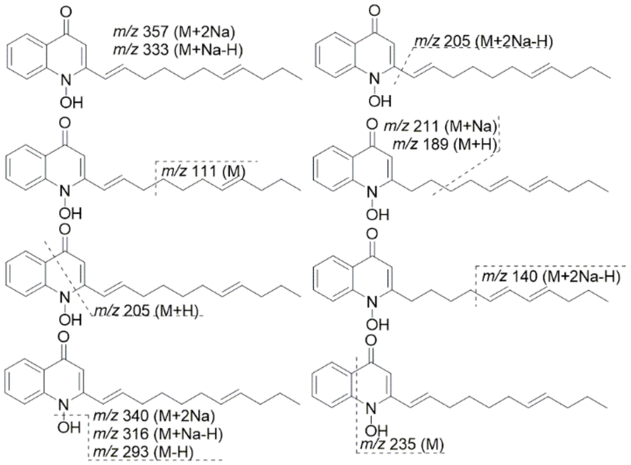
m/z 357 (M+2Na)
m/z 333 (M+Na-H)
m/z 205 (M+2Na-H)
m/z 111 (M)
m/z 211 (M+Na)
m/z 189 (M+H)
m/z 205 (M+H)
m/z 140 (M+2Na-H)
m/z 340 (M+2Na)
m/z 316 (M+Na-H)
m/z 293 (M-H)
m/z 235 (M)
O
N
OH

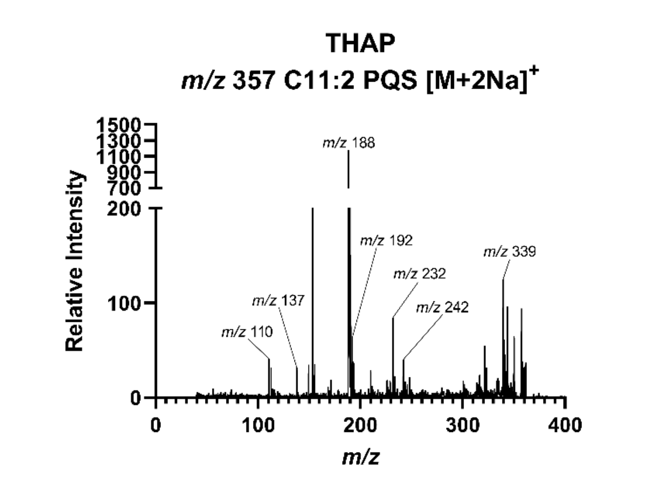
THAP
m/z 357 C11:2 PQS [M+2Na]+
Relative Intensity
m/z
m/z 188
m/z 192
m/z 232
m/z 242
m/z 339
m/z 137
m/z 110

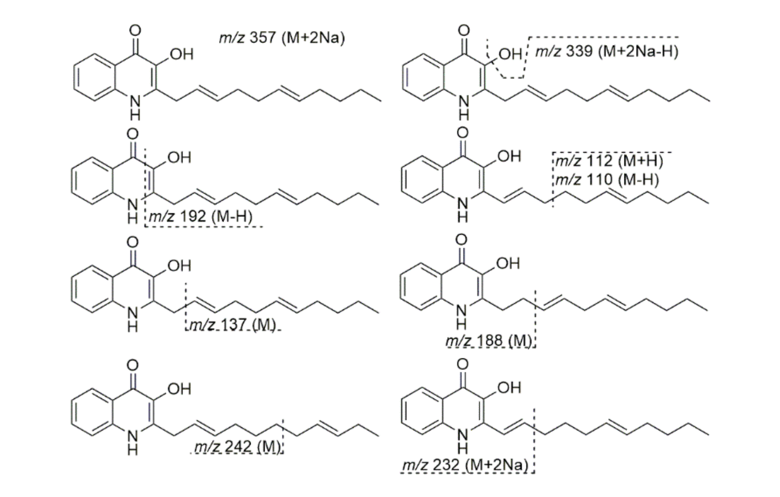
m/z 357 (M+2Na)
m/z 339 (M+2Na-H)
m/z 192 (M-H)
m/z 112 (M+H)
m/z 110 (M-H)
m/z 137 (M)
m/z 188 (M)
m/z 242 (M)
m/z 232 (M+2Na)

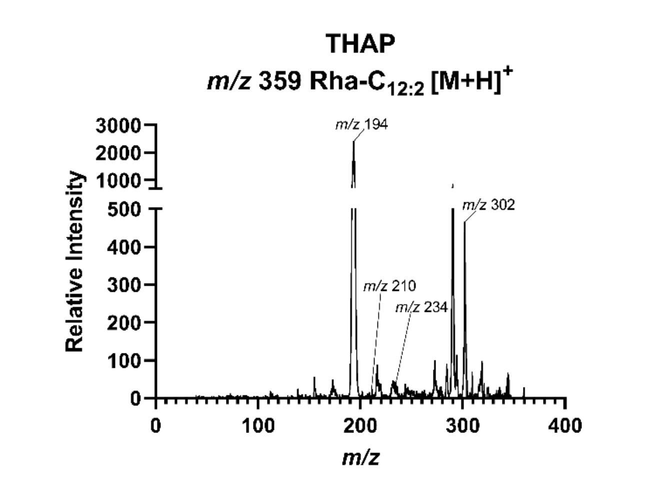
THAP
m/z 359 Rha-C12:2 [M+H]+
Relative Intensity
m/z
m/z 194
m/z 302
m/z 210
m/z 234

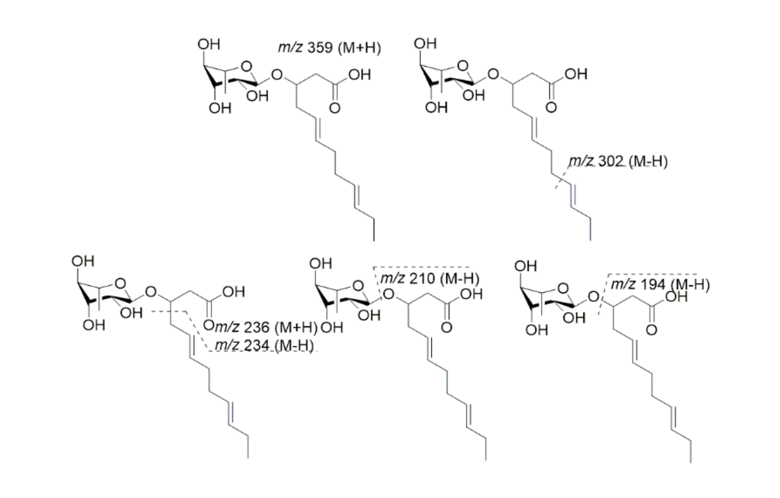
m/z 359 (M+H)
m/z 302 (M-H)
m/z 236 (M+H)
m/z 234 (M-H)
m/z 210 (M-H)
m/z 194 (M-H)

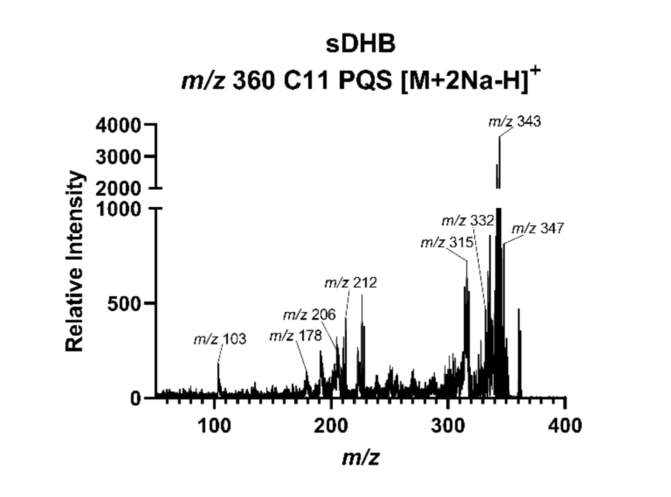
sDHB
m/z 360 C11 PQS [M+2Na-H]+
Relative Intensity
m/z
m/z 343
m/z 332
m/z 347
m/z 315
m/z 212
m/z 206
m/z 178
m/z 103

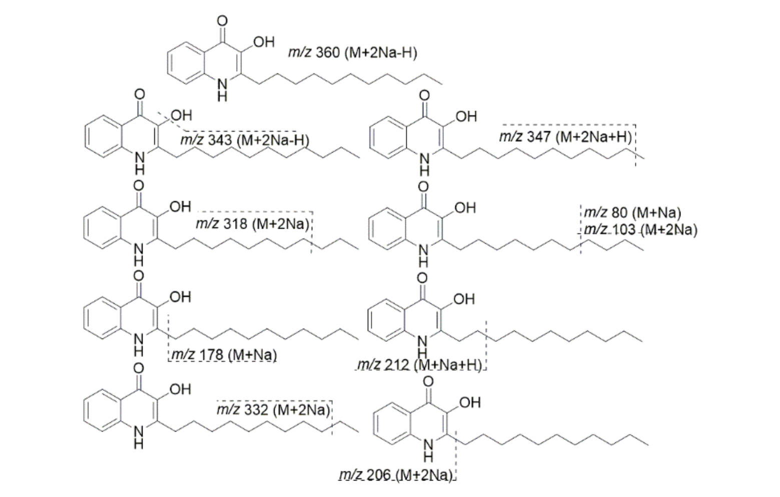
m/z 360 (M+2Na-H)
m/z 343 (M+2Na-H)
m/z 347 (M+2Na+H)
m/z 318 (M+2Na)
m/z 80 (M+Na)
m/z 103 (M+2Na)
m/z 178 (M+Na)
m/z 212 (M+Na+H)
m/z 332 (M+2Na)
m/z 206 (M+2Na)

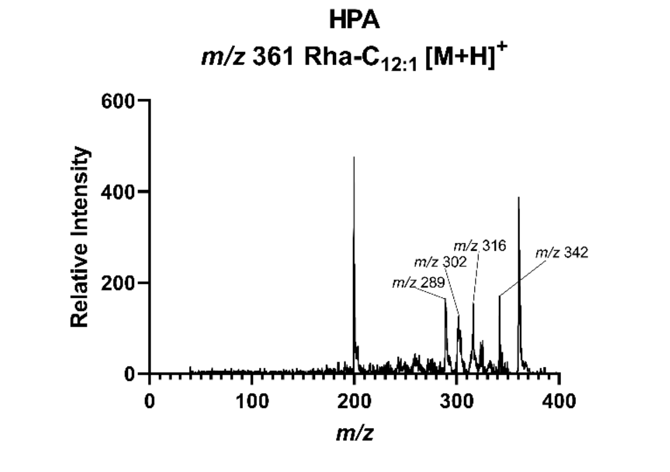
HPA
m/z 361 Rha-C12:1 [M+H]+
Relative Intensity
m/z
m/z 289
m/z 302
m/z 316
m/z 342

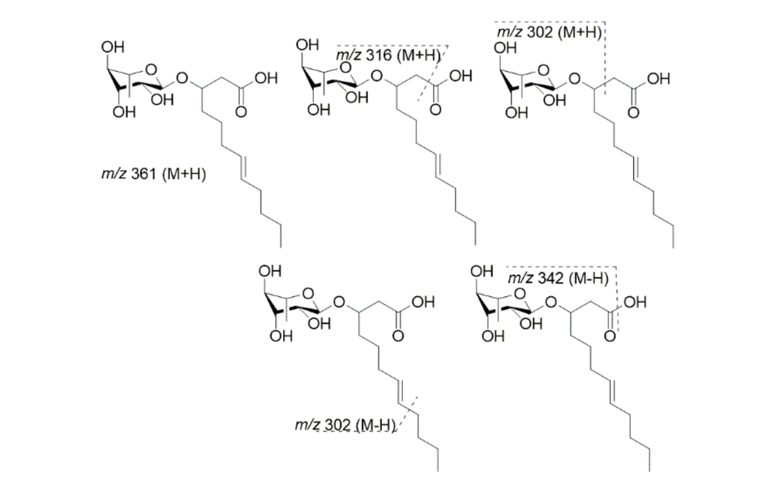
m/z 361 (M+H)
m/z 316 (M+H)
m/z 302 (M+H)
m/z 302 (M-H)
m/z 342 (M-H)

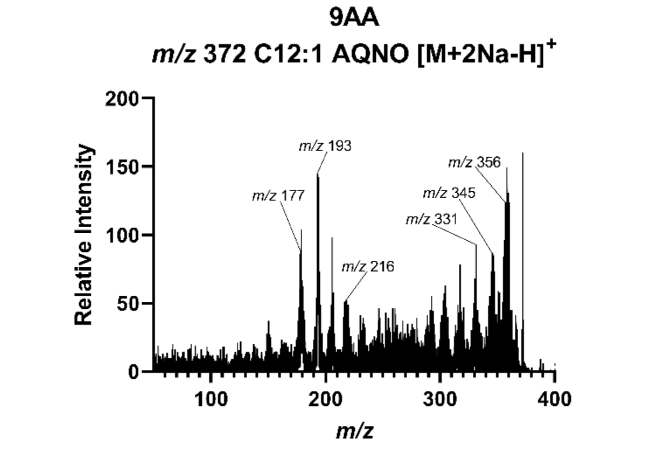
9AA
m/z 372 C12:1 AQNO [M+2Na-H]+
Relative Intensity
m/z
m/z 193
m/z 177
m/z 216
m/z 356
m/z 345
m/z 331

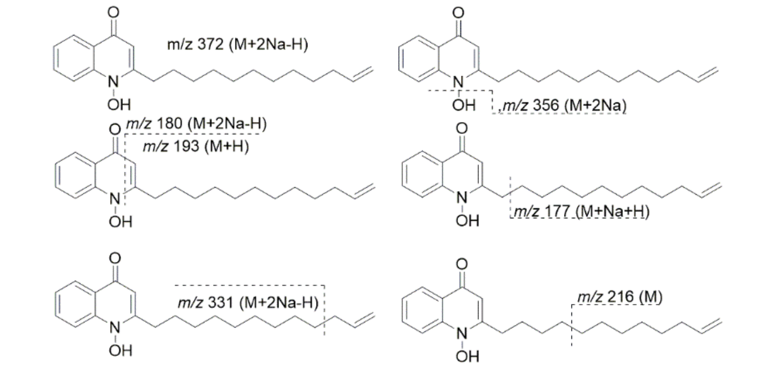
m/z 372 (M+2Na-H)
m/z 356 (M+2Na)
m/z 180 (M+2Na-H)
m/z 193 (M+H)
m/z 177 (M+Na+H)
m/z 331 (M+2Na-H)
m/z 216 (M)

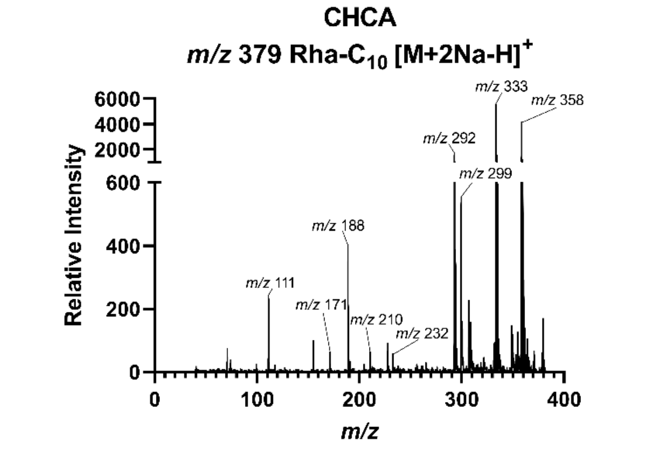
CHCA
m/z 379 Rha-C10 [M+2Na-H]+
Relative Intensity
m/z
m/z 333
m/z 358
m/z 292
m/z 299
m/z 188
m/z 111
m/z 171
m/z 210
m/z 232

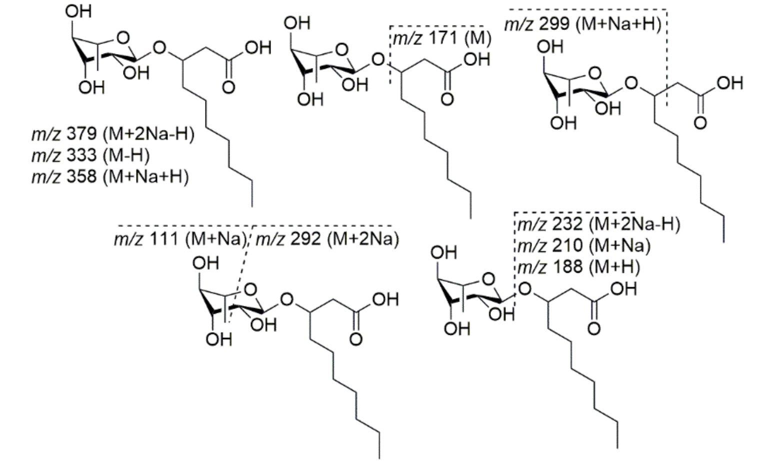
m/z 379 (M+2Na-H)
m/z 333 (M-H)
m/z 358 (M+Na+H)
m/z 171 (M)
m/z 299 (M+Na+H)
m/z 111 (M+Na)
m/z 292 (M+2Na)
m/z 232 (M+2Na-H)
m/z 210 (M+Na)
m/z 188 (M+H)

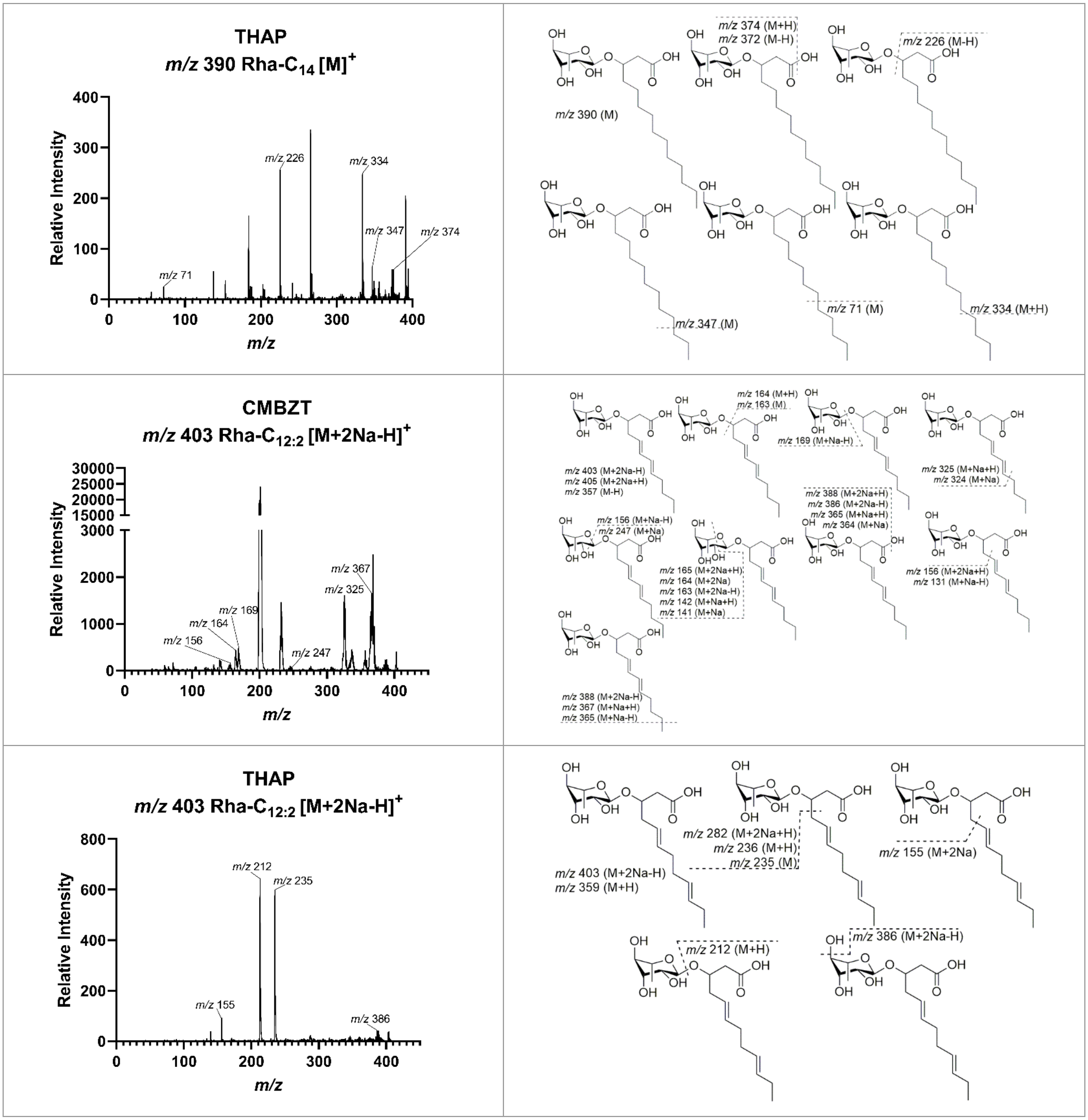
THAP
m/z 390 Rha-C14 [M]+
Relative Intensity
m/z
m/z 71
m/z 226
m/z 334
m/z 347
m/z 374
m/z 374 (M+H)
m/z 372 (M-H)
m/z 226 (M-H)
m/z 390 (M)
m/z 347 (M)
m/z 71 (M)
m/z 334 (M+H)
CMBZT
m/z 403 Rha-C12:2 [M+2Na-H]+
m/z 156
m/z 164
m/z 169
m/z 247
m/z 325
m/z 367
m/z 164 (M+H)
m/z 163 (M)
m/z 169 (M+Na-H)
m/z 403 (M+2Na-H)
m/z 405 (M+2Na+H)
m/z 357 (M-H)
m/z 325 (M+Na+H)
m/z 324 (M+Na)
m/z 388 (M+2Na+H)
m/z 386 (M+2Na-H)
m/z 365 (M+Na+H)
m/z 364 (M+Na)
m/z 156 (M+Na-H)
m/z 247 (M+Na)
m/z 165 (M+2Na+H)
m/z 164 (M+2Na)
m/z 163 (M+2Na-H)
m/z 142 (M+Na+H)
m/z 141 (M+Na)
m/z 156 (M+2Na+H)
m/z 131 (M+Na-H)
m/z 388 (M+2Na-H)
m/z 367 (M+Na+H)
m/z 365 (M+Na-H)
THAP
m/z 403 Rha-C12:2 [M+2Na-H]+
m/z 155
m/z 212
m/z 235
m/z 386
m/z 403 (M+2Na-H)
m/z 359 (M+H)
m/z 282 (M+2Na+H)
m/z 236 (M+H)
m/z 235 (M)
m/z 155 (M+2Na)
m/z 212 (M+H)
m/z 386 (M+2Na-H)

CHCA
***m/z* 453 LPA (16:1) [M+2Na]$^+$**

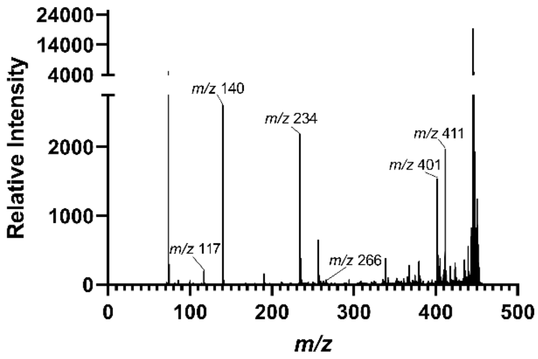

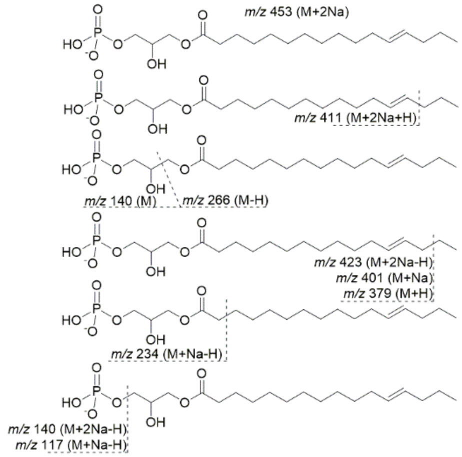

HPA
***m/z* 472 Rha-C_8-C_8 [M+Na+H]$^+$**

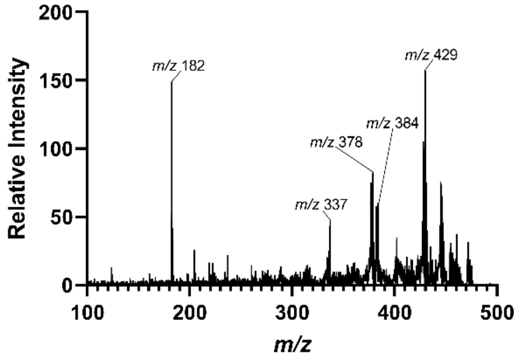

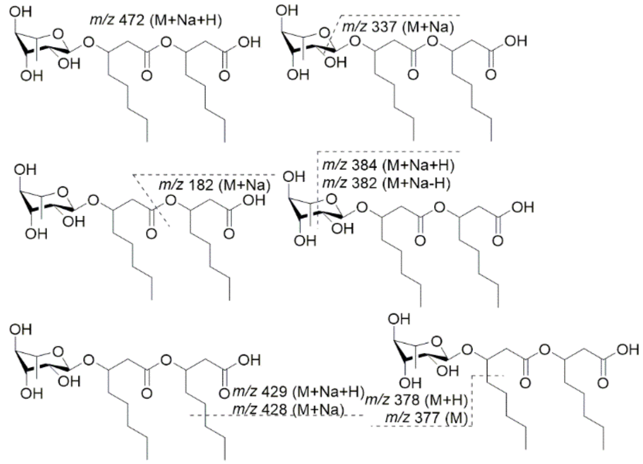

9AA
***m/z* 474 Rha-Rha-C_8 [M+Na-H]$^+$**

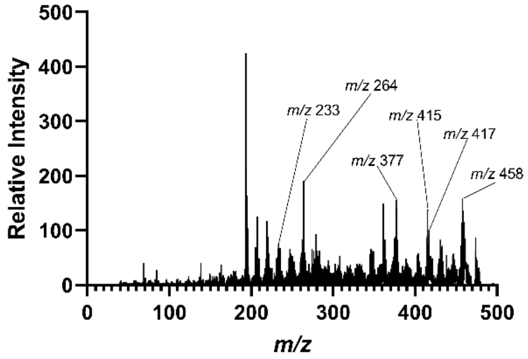

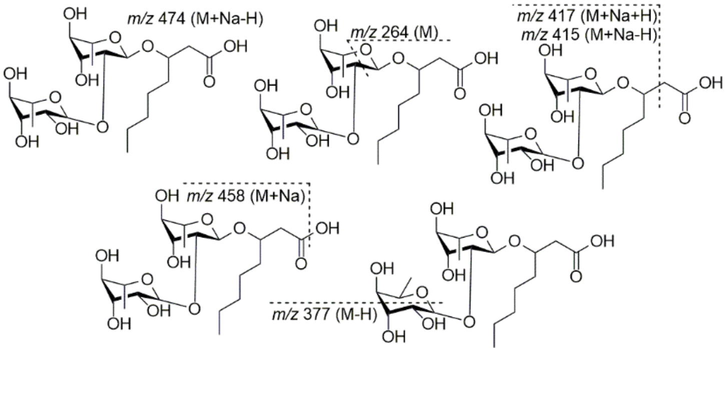

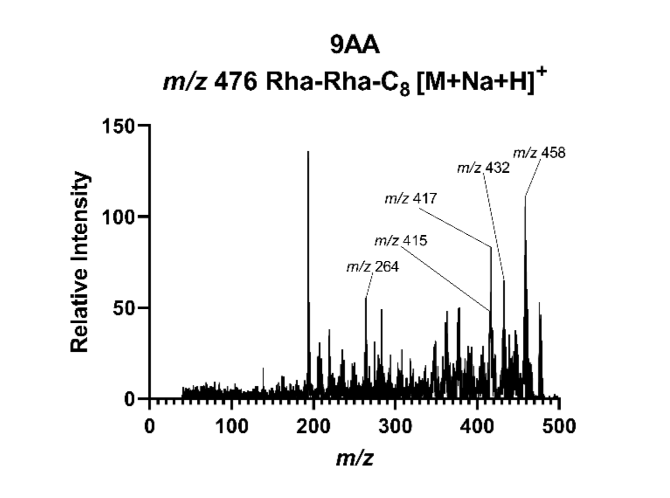
9AA
m/z 476 Rha-Rha-C8 [M+Na+H]+
Relative Intensity
m/z
m/z 458
m/z 432
m/z 417
m/z 415
m/z 264

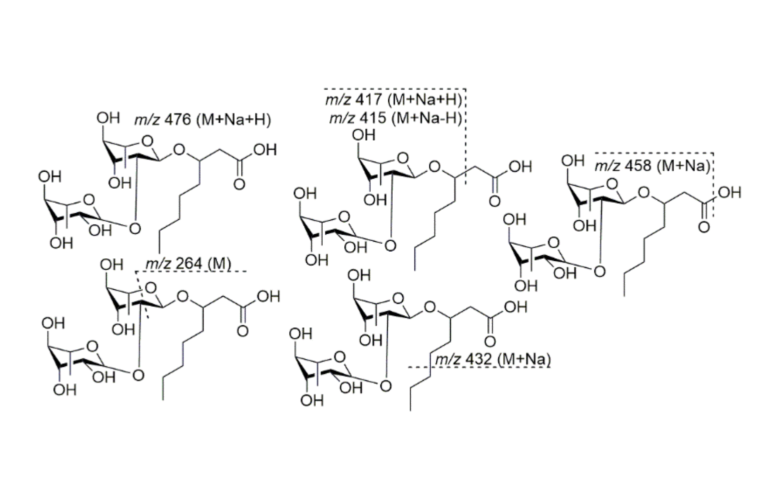
m/z 476 (M+Na+H)
m/z 417 (M+Na+H)
m/z 415 (M+Na-H)
m/z 458 (M+Na)
m/z 264 (M)
m/z 432 (M+Na)

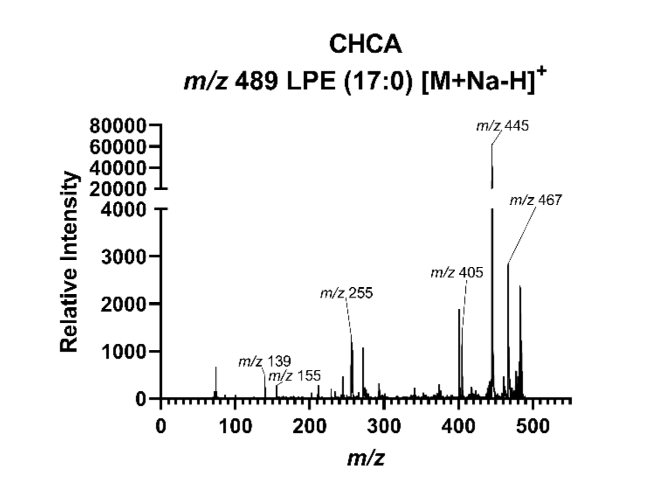
CHCA
m/z 489 LPE (17:0) [M+Na-H]+
Relative Intensity
m/z
m/z 445
m/z 467
m/z 405
m/z 255
m/z 139
m/z 155

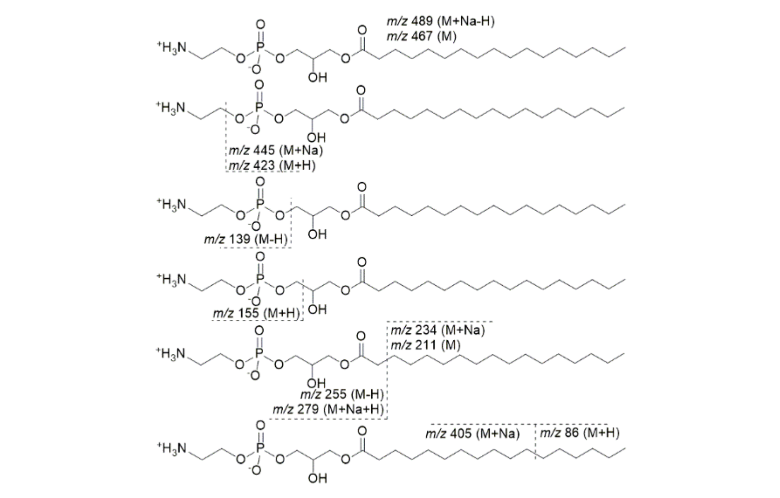
m/z 489 (M+Na-H)
m/z 467 (M)
m/z 445 (M+Na)
m/z 423 (M+H)
m/z 139 (M-H)
m/z 155 (M+H)
m/z 234 (M+Na)
m/z 211 (M)
m/z 255 (M-H)
m/z 279 (M+Na+H)
m/z 405 (M+Na)
m/z 86 (M+H)

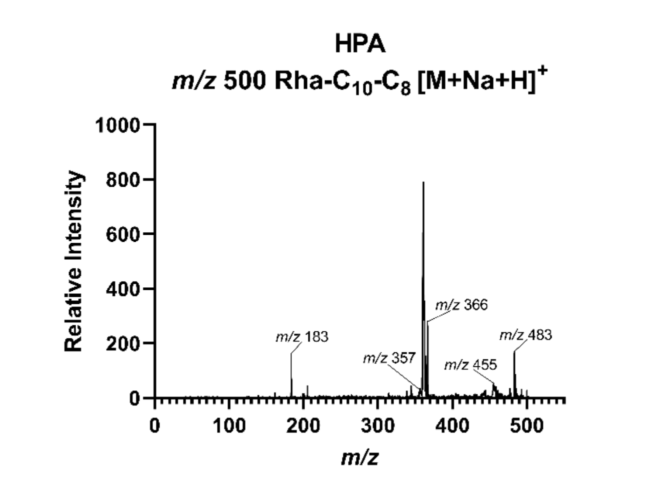
HPA
m/z 500 Rha-C10-C8 [M+Na+H]+
Relative Intensity
m/z
m/z 183
m/z 357
m/z 366
m/z 455
m/z 483

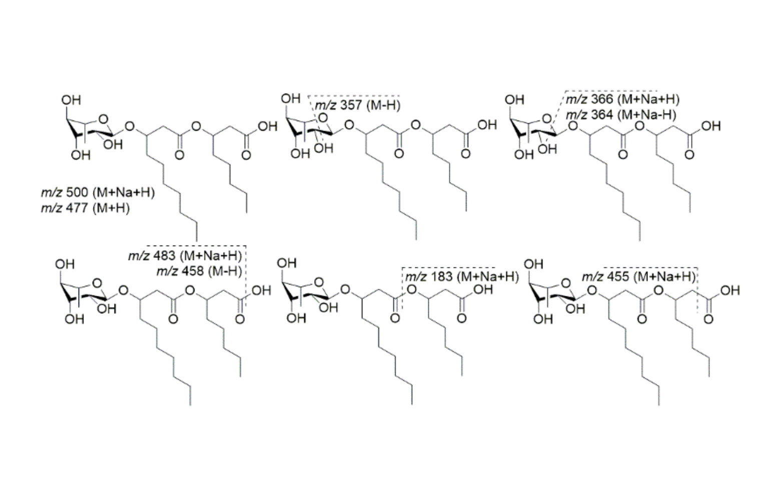
m/z 500 (M+Na+H)
m/z 477 (M+H)
m/z 357 (M-H)
m/z 366 (M+Na+H)
m/z 364 (M+Na-H)
m/z 483 (M+Na+H)
m/z 458 (M-H)
m/z 183 (M+Na+H)
m/z 455 (M+Na+H)

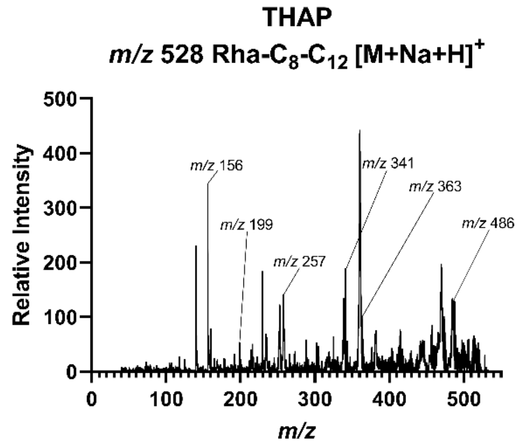
THAP
m/z 528 Rha-C8-C12 [M+Na+H]+
Relative Intensity
m/z
m/z 156
m/z 199
m/z 257
m/z 341
m/z 363
m/z 486

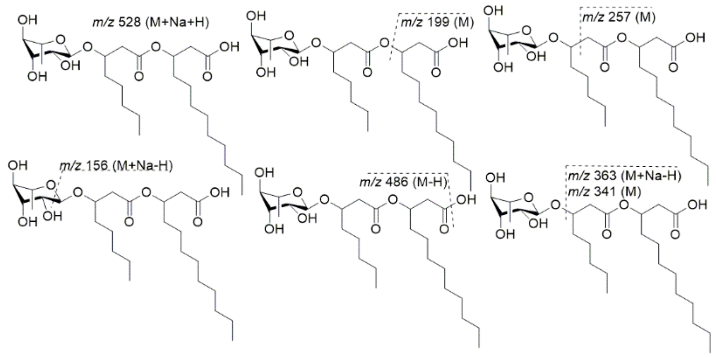
m/z 528 (M+Na+H)
m/z 199 (M)
m/z 257 (M)
m/z 156 (M+Na-H)
m/z 486 (M-H)
m/z 363 (M+Na-H)
m/z 341 (M)

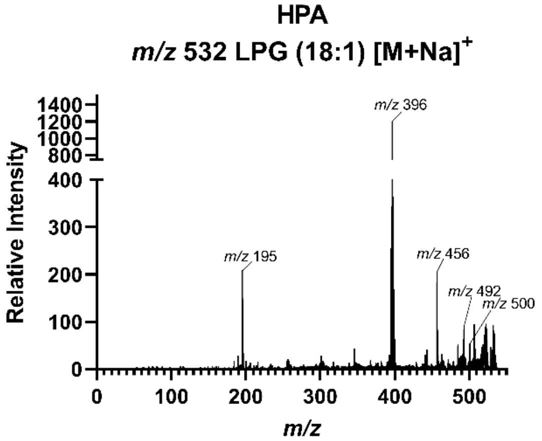
HPA
m/z 532 LPG (18:1) [M+Na]+
Relative Intensity
m/z
m/z 396
m/z 195
m/z 456
m/z 492
m/z 500

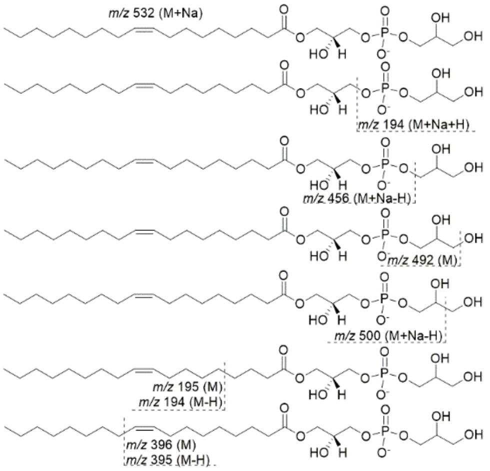
m/z 532 (M+Na)
m/z 194 (M+Na+H)
m/z 456 (M+Na-H)
m/z 492 (M)
m/z 500 (M+Na-H)
m/z 195 (M)
m/z 194 (M-H)
m/z 396 (M)
m/z 395 (M-H)

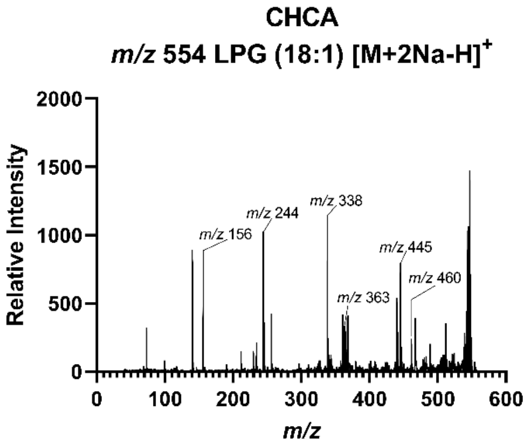
CHCA
m/z 554 LPG (18:1) [M+2Na-H]+
Relative Intensity
m/z
m/z 156
m/z 244
m/z 338
m/z 363
m/z 445
m/z 460

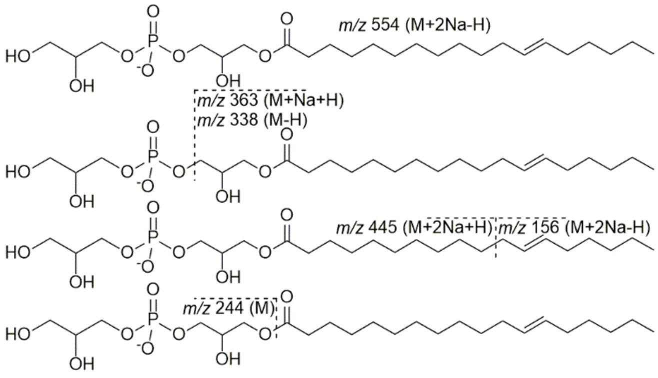
m/z 554 (M+2Na-H)
m/z 363 (M+Na+H)
m/z 338 (M-H)
m/z 445 (M+2Na+H)
m/z 156 (M+2Na-H)
m/z 244 (M)

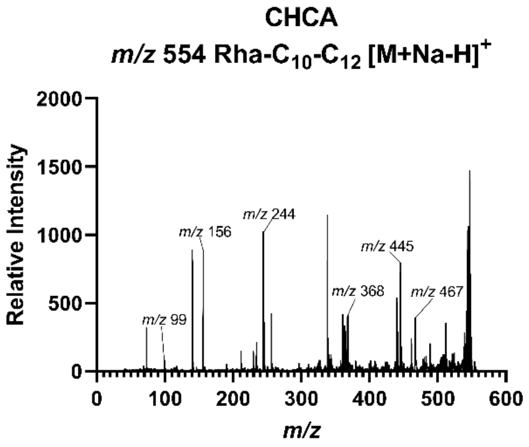
CHCA
m/z 554 Rha-C10-C12 [M+Na-H]+
Relative Intensity
m/z
m/z 99
m/z 156
m/z 244
m/z 368
m/z 445
m/z 467
0
500
1000
1500
2000
100
200
300
400
500
600

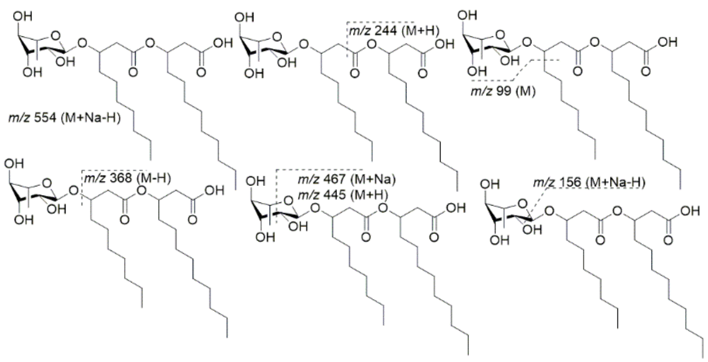
m/z 554 (M+Na-H)
m/z 244 (M+H)
m/z 99 (M)
m/z 368 (M-H)
m/z 467 (M+Na)
m/z 445 (M+H)
m/z 156 (M+Na-H)

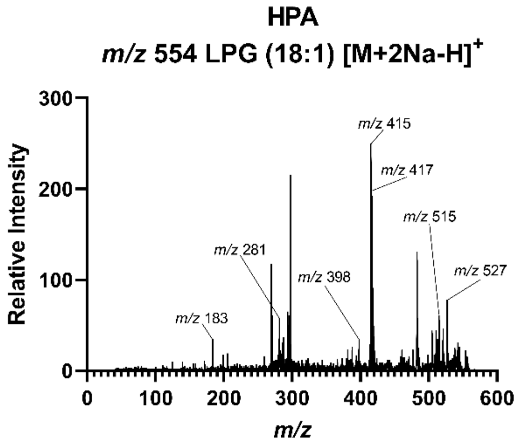
HPA
m/z 554 LPG (18:1) [M+2Na-H]+
Relative Intensity
m/z
m/z 183
m/z 281
m/z 398
m/z 415
m/z 417
m/z 515
m/z 527
0
100
200
300
400
500
600

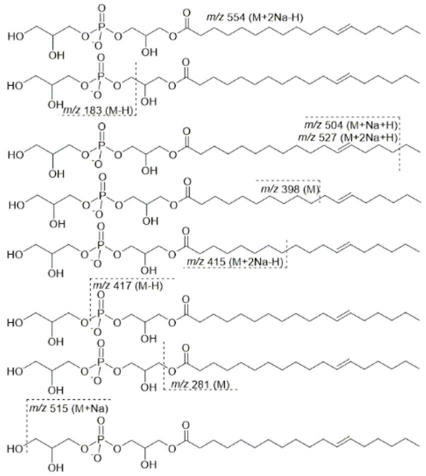
m/z 554 (M+2Na-H)
m/z 183 (M-H)
m/z 504 (M+Na+H)
m/z 527 (M+2Na+H)
m/z 398 (M)
m/z 415 (M+2Na-H)
m/z 417 (M-H)
m/z 281 (M)
m/z 515 (M+Na)

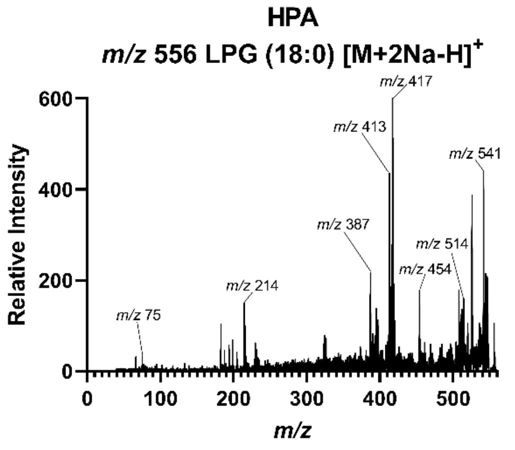
HPA
m/z 556 LPG (18:0) [M+2Na-H]+
Relative Intensity
m/z
m/z 75
m/z 214
m/z 387
m/z 413
m/z 417
m/z 454
m/z 514
m/z 541
0
200
400
600
100
300
500

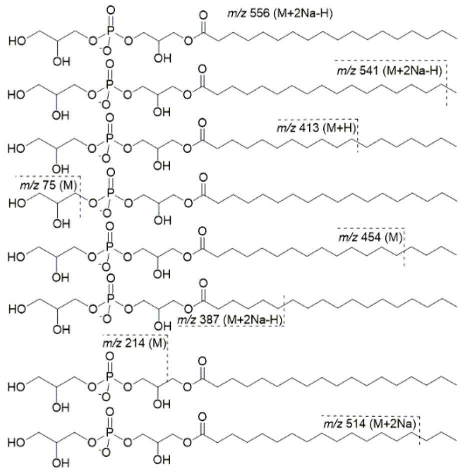
m/z 556 (M+2Na-H)
m/z 541 (M+2Na-H)
m/z 413 (M+H)
m/z 75 (M)
m/z 454 (M)
m/z 387 (M+2Na-H)
m/z 214 (M)
m/z 514 (M+2Na)

CMBZT

***m/z* 575 Rha-$C_{14:1}$-C_8 [M+2Na-H]$^+$**

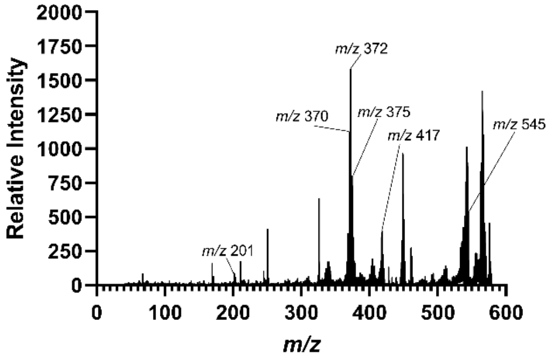

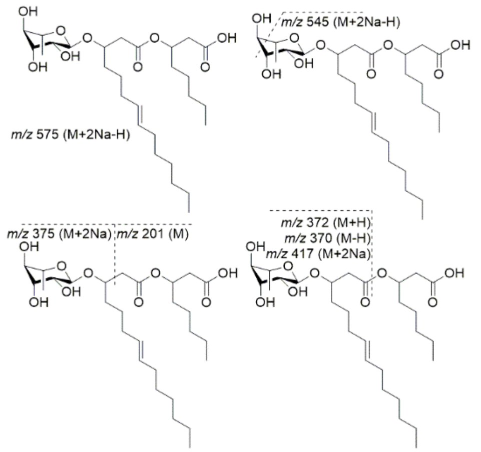

sDHB

***m/z* 575 Rha $C_{12:1}$-C_{10} [M+2Na-H]$^+$**

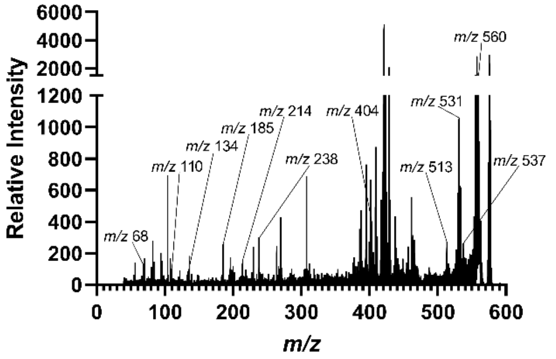

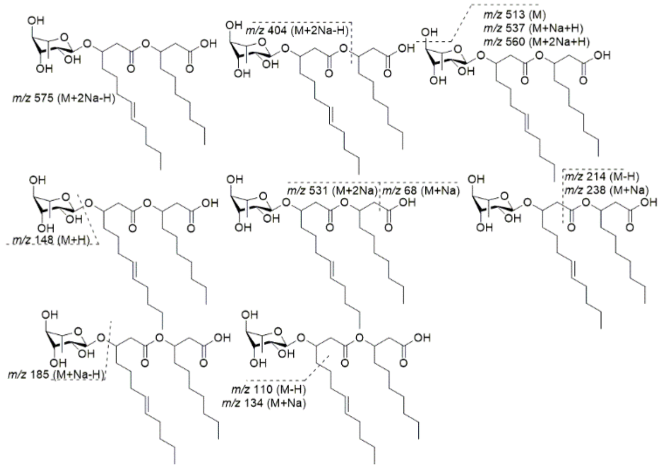

sDHB

***m/z* 577 Rha-C_{14}-C_8 [M+2Na-H]$^+$**

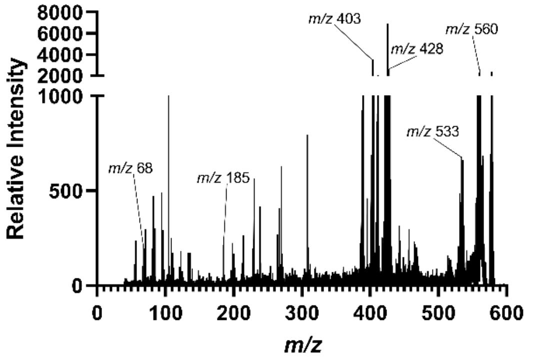

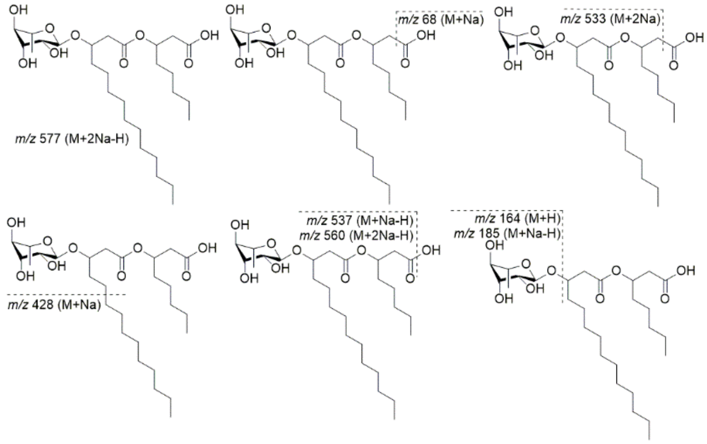

CHCA

m/z 605 Rha-C_{14}-C_{10} $[M+2Na-H]^+$

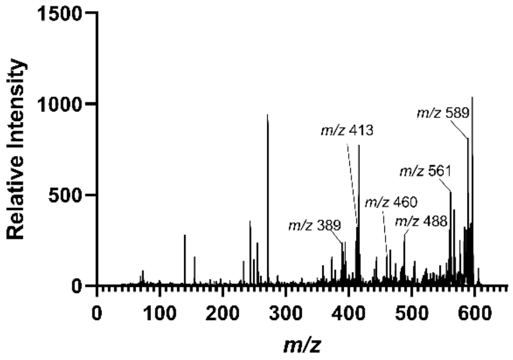

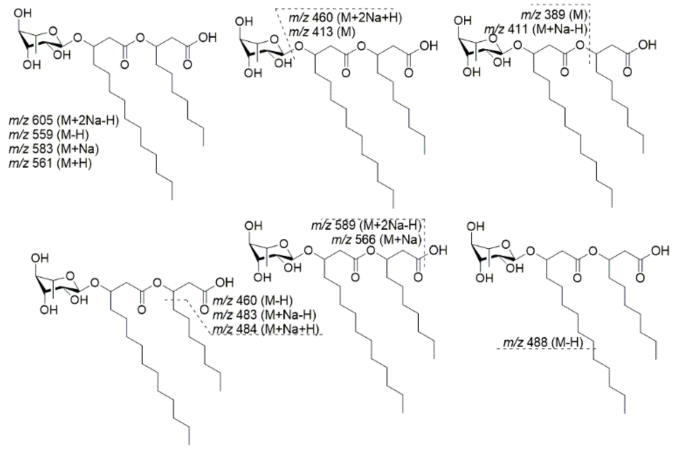

CMBZT

m/z 617 Rha-C_{14}-C_{14} $[M+H]^+$

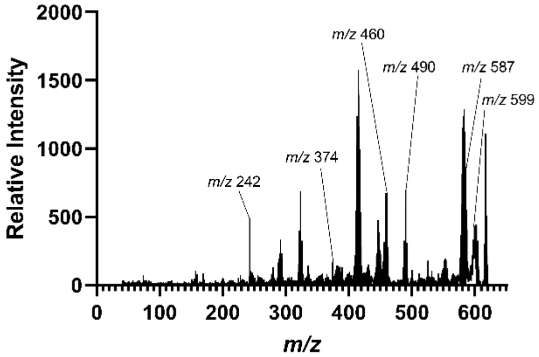

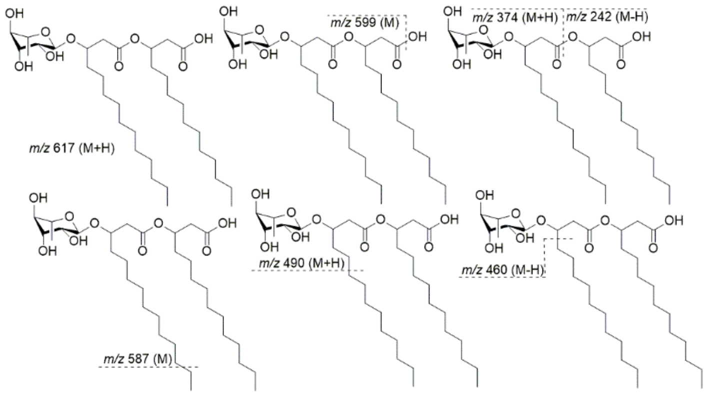

HPA

m/z 633 Rha-C_{12}-C_{14} $[M+2Na-H]^+$

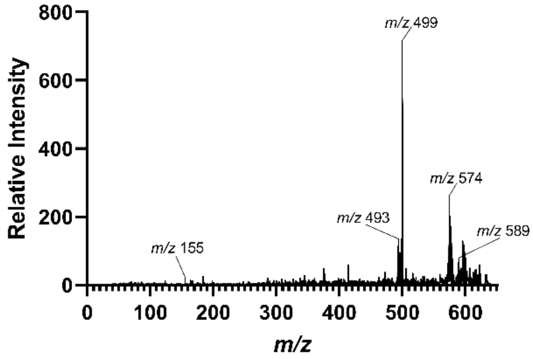

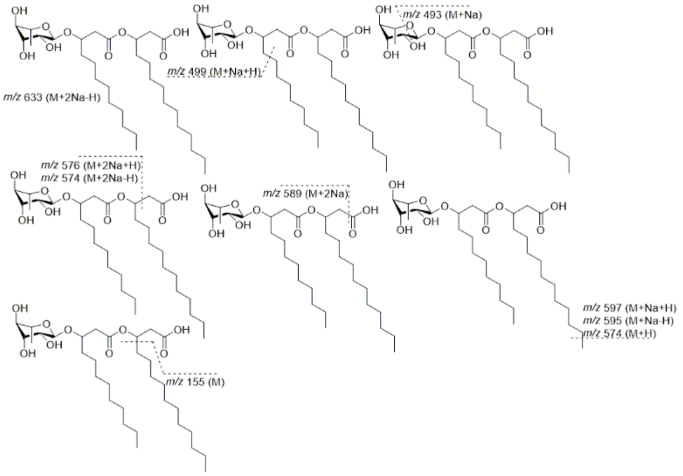

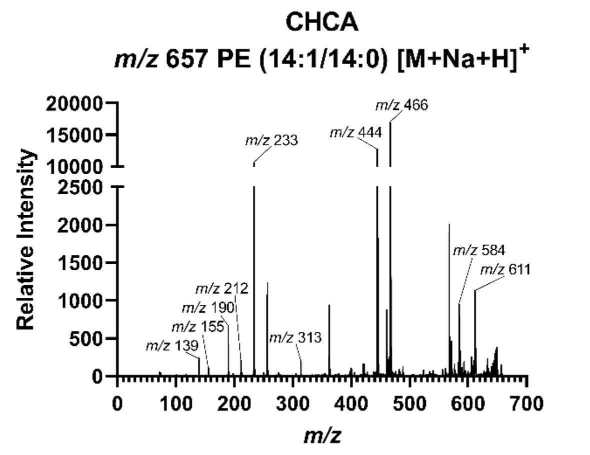
CHCA
m/z 657 PE (14:1/14:0) [M+Na+H]+
Relative Intensity
m/z
m/z 466
m/z 444
m/z 233
m/z 584
m/z 611
m/z 212
m/z 190
m/z 155
m/z 139
m/z 313

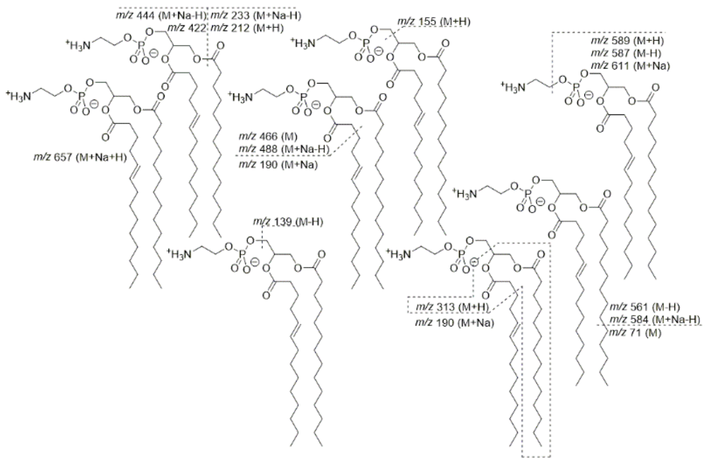
m/z 444 (M+Na-H)
m/z 233 (M+Na-H)
m/z 422
m/z 212 (M+H)
m/z 155 (M+H)
m/z 589 (M+H)
m/z 587 (M-H)
m/z 611 (M+Na)
m/z 657 (M+Na+H)
m/z 466 (M)
m/z 488 (M+Na-H)
m/z 190 (M+Na)
m/z 139 (M-H)
m/z 313 (M+H)
m/z 190 (M+Na)
m/z 561 (M-H)
m/z 584 (M+Na-H)
m/z 71 (M)

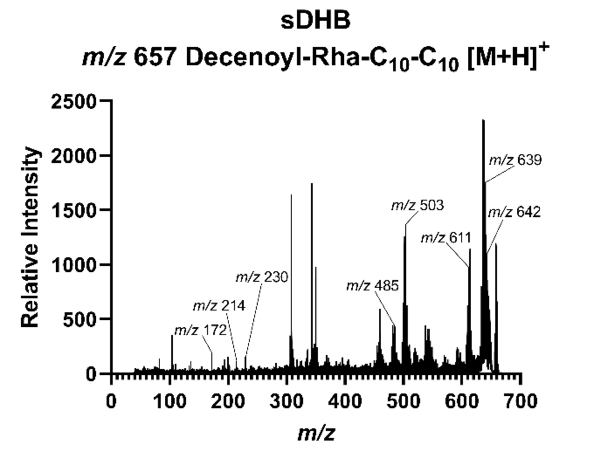
sDHB
m/z 657 Decenoyl-Rha-C10-C10 [M+H]+
Relative Intensity
m/z
m/z 639
m/z 503
m/z 642
m/z 611
m/z 230
m/z 485
m/z 214
m/z 172

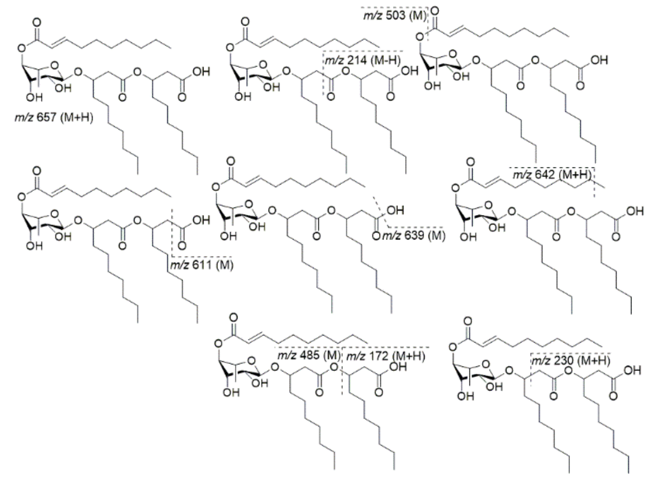
m/z 657 (M+H)
m/z 214 (M-H)
m/z 503 (M)
m/z 611 (M)
m/z 639 (M)
m/z 642 (M+H)
m/z 485 (M)
m/z 172 (M+H)
m/z 230 (M+H)

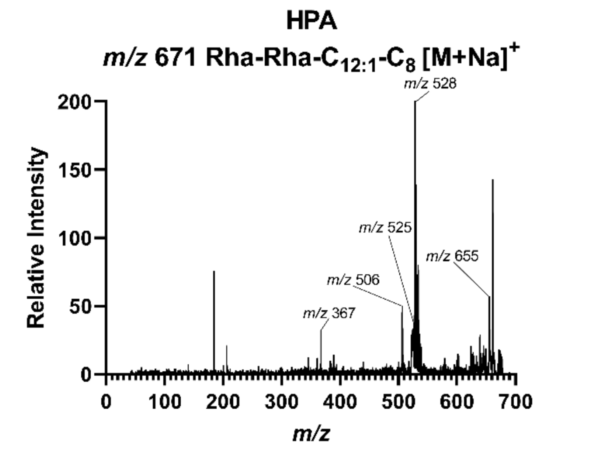
HPA
m/z 671 Rha-Rha-C12:1-C8 [M+Na]+
Relative Intensity
m/z
m/z 528
m/z 525
m/z 655
m/z 506
m/z 367

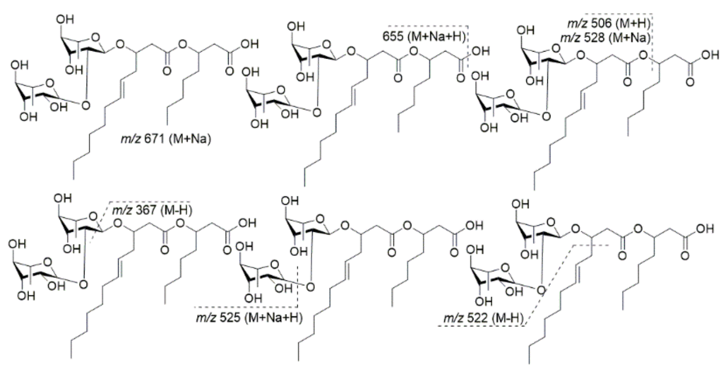
m/z 671 (M+Na)
655 (M+Na+H)
m/z 506 (M+H)
m/z 528 (M+Na)
m/z 367 (M-H)
m/z 525 (M+Na+H)
m/z 522 (M-H)

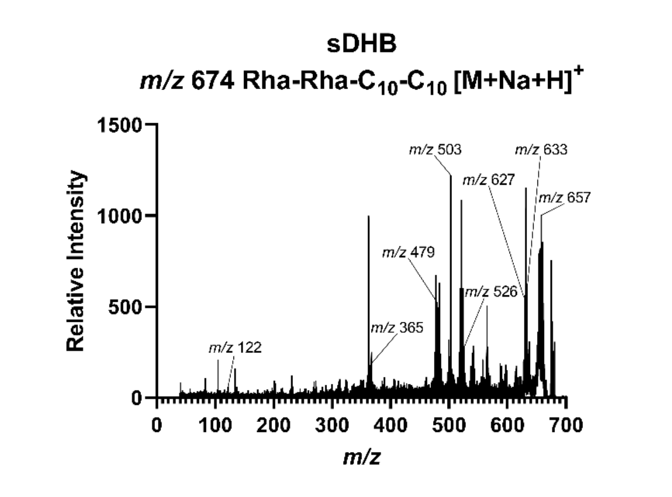
sDHB
m/z 674 Rha-Rha-C10-C10 [M+Na+H]+
Relative Intensity
m/z
m/z 503
m/z 633
m/z 627
m/z 657
m/z 479
m/z 526
m/z 365
m/z 122

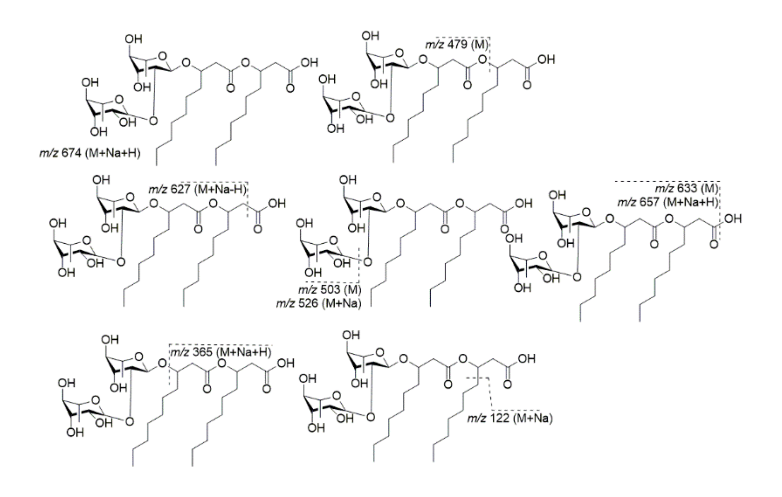
m/z 674 (M+Na+H)
m/z 479 (M)
m/z 627 (M+Na-H)
m/z 503 (M)
m/z 526 (M+Na)
m/z 633 (M)
m/z 657 (M+Na+H)
m/z 365 (M+Na+H)
m/z 122 (M+Na)

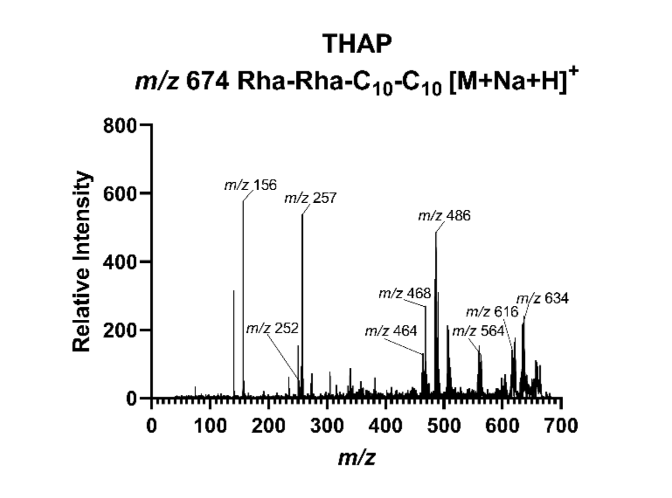
THAP
m/z 674 Rha-Rha-C10-C10 [M+Na+H]+
Relative Intensity
m/z
m/z 156
m/z 257
m/z 486
m/z 468
m/z 634
m/z 616
m/z 252
m/z 464
m/z 564

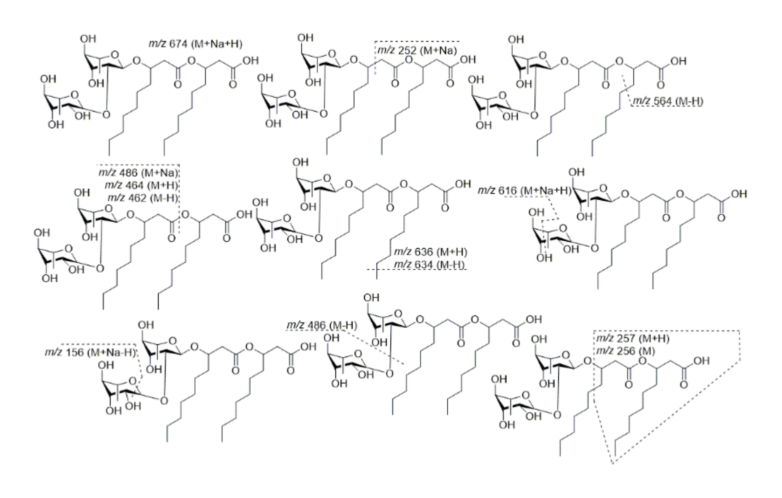
m/z 674 (M+Na+H)
m/z 252 (M+Na)
m/z 564 (M-H)
m/z 486 (M+Na)
m/z 464 (M+H)
m/z 462 (M-H)
m/z 636 (M+H)
m/z 634 (M-H)
m/z 616 (M+Na+H)
m/z 156 (M+Na-H)
m/z 486 (M-H)
m/z 257 (M+H)
m/z 256 (M)

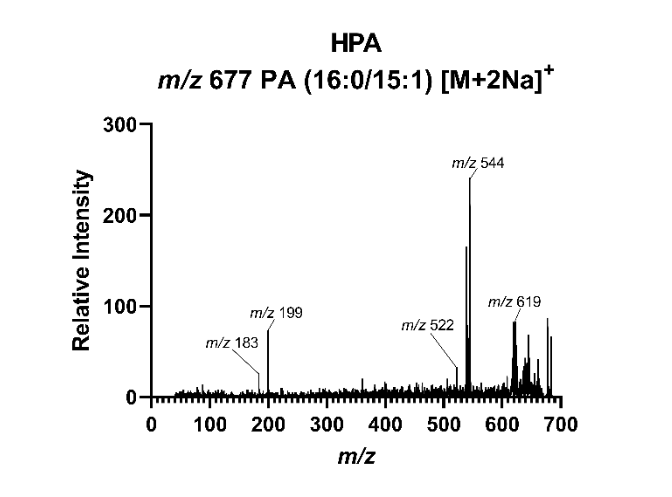
HPA
m/z 677 PA (16:0/15:1) [M+2Na]+
Relative Intensity
m/z
m/z 544
m/z 619
m/z 199
m/z 522
m/z 183

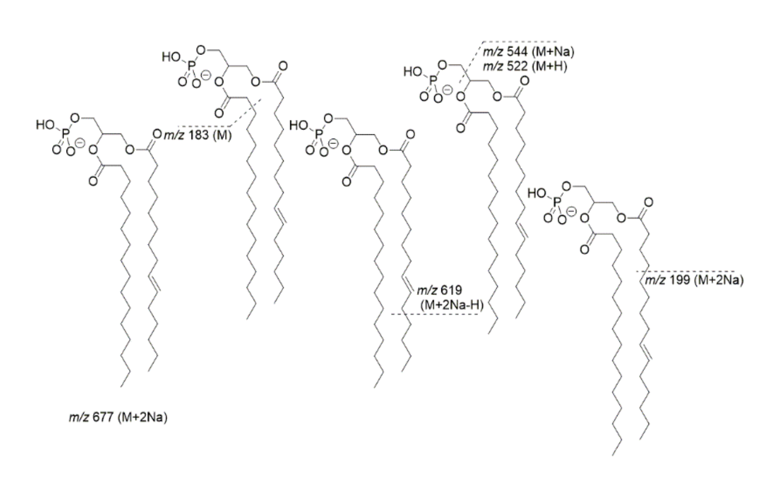
m/z 183 (M)
m/z 544 (M+Na)
m/z 522 (M+H)
m/z 619
(M+2Na-H)
m/z 199 (M+2Na)
m/z 677 (M+2Na)

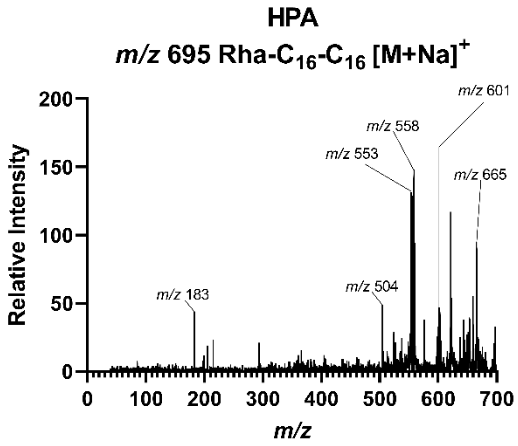
HPA
m/z 695 Rha-C16-C16 [M+Na]+
Relative Intensity
m/z
m/z 601
m/z 558
m/z 553
m/z 665
m/z 183
m/z 504

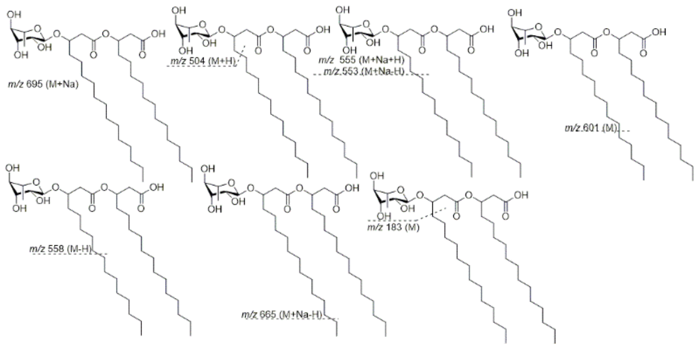
m/z 695 (M+Na)
m/z 504 (M+H)
m/z 555 (M+Na+H)
m/z 553 (M+Na-H)
m/z 601 (M)
m/z 558 (M-H)
m/z 183 (M)
m/z 665 (M+Na-H)

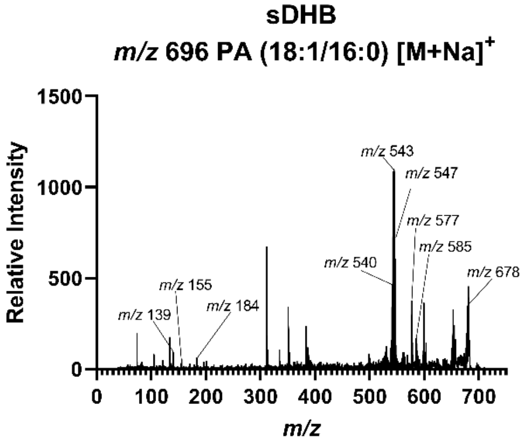
sDHB
m/z 696 PA (18:1/16:0) [M+Na]+
Relative Intensity
m/z
m/z 543
m/z 547
m/z 577
m/z 585
m/z 540
m/z 678
m/z 155
m/z 139
m/z 184

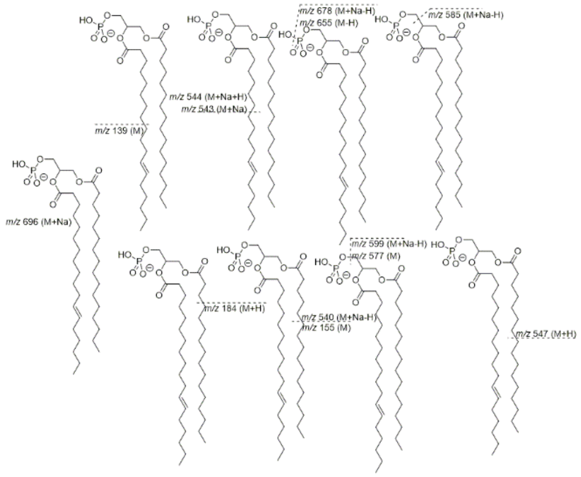
m/z 678 (M+Na-H)
m/z 655 (M-H)
m/z 585 (M+Na-H)
m/z 544 (M+Na+H)
m/z 543 (M+Na)
m/z 139 (M)
m/z 696 (M+Na)
m/z 599 (M+Na-H)
m/z 577 (M)
m/z 184 (M+H)
m/z 540 (M+Na-H)
m/z 155 (M)
m/z 547 (M+H)

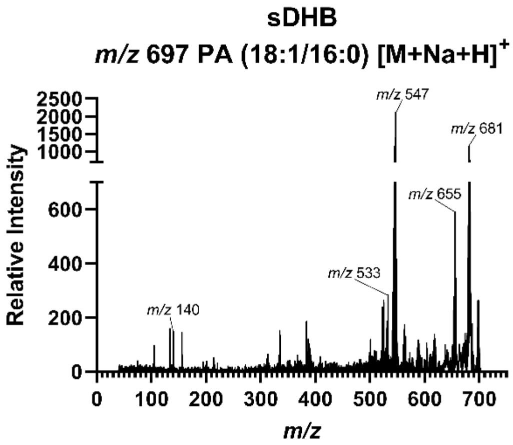
sDHB
m/z 697 PA (18:1/16:0) [M+Na+H]+
Relative Intensity
m/z
m/z 547
m/z 681
m/z 655
m/z 533
m/z 140

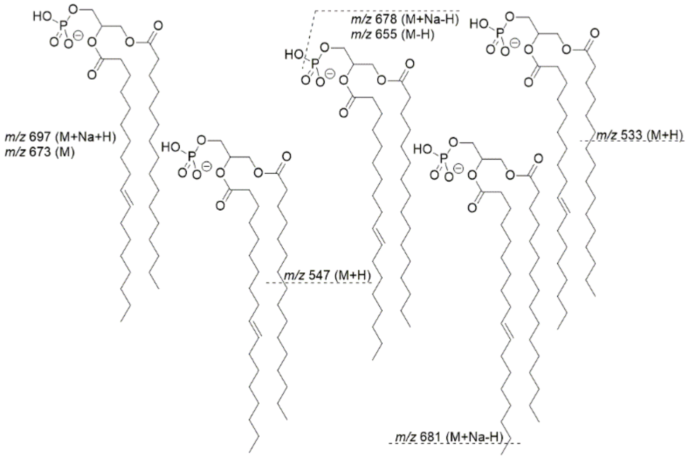
m/z 697 (M+Na+H)
m/z 673 (M)
m/z 678 (M+Na-H)
m/z 655 (M-H)
m/z 533 (M+H)
m/z 547 (M+H)
m/z 681 (M+Na-H)

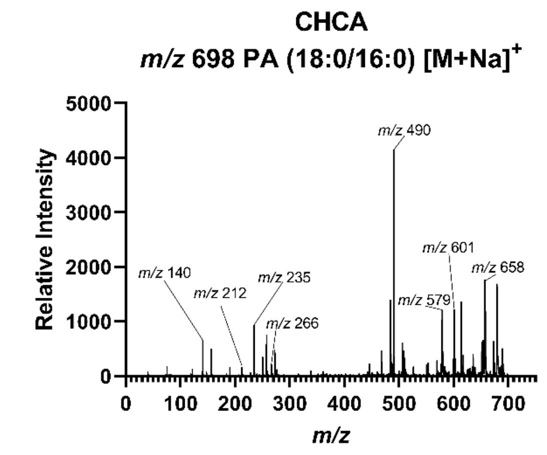
CHCA
m/z 698 PA (18:0/16:0) [M+Na]+
Relative Intensity
m/z
m/z 490
m/z 601
m/z 658
m/z 140
m/z 212
m/z 235
m/z 579
m/z 266

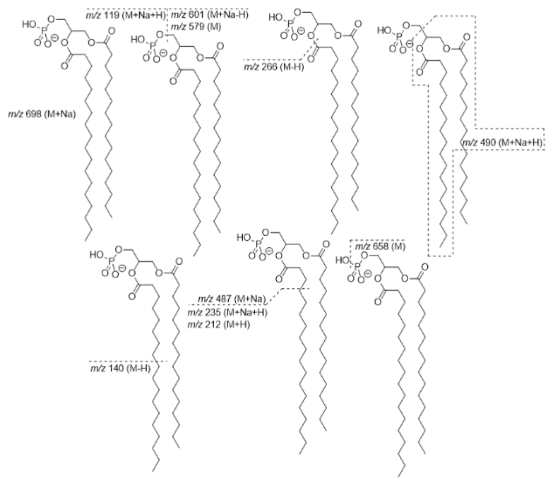
m/z 119 (M+Na+H)
m/z 601 (M+Na-H)
m/z 579 (M)
m/z 266 (M-H)
m/z 698 (M+Na)
m/z 490 (M+Na+H)
m/z 658 (M)
m/z 487 (M+Na)
m/z 235 (M+Na+H)
m/z 212 (M+H)
m/z 140 (M-H)

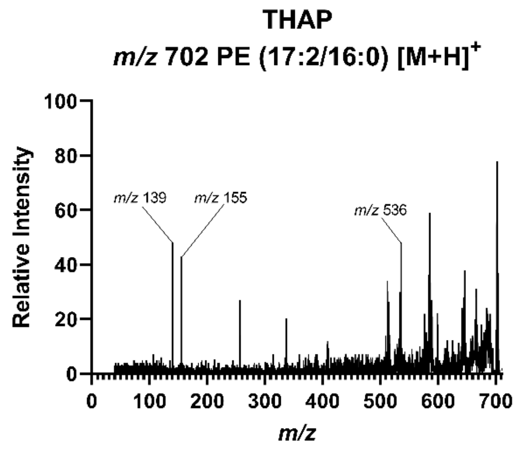
THAP
m/z 702 PE (17:2/16:0) [M+H]+
Relative Intensity
m/z
m/z 139
m/z 155
m/z 536

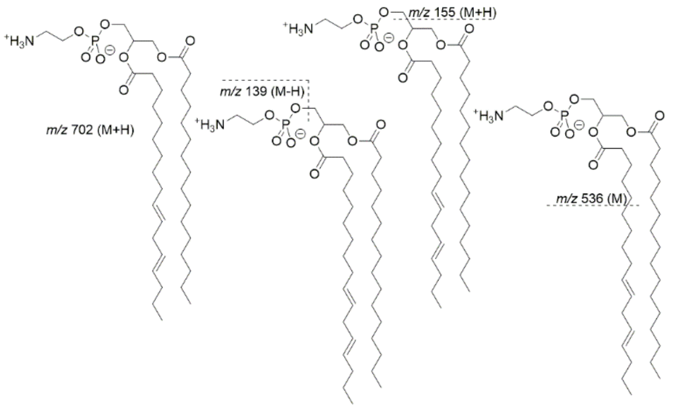
m/z 155 (M+H)
m/z 139 (M-H)
m/z 702 (M+H)
m/z 536 (M)

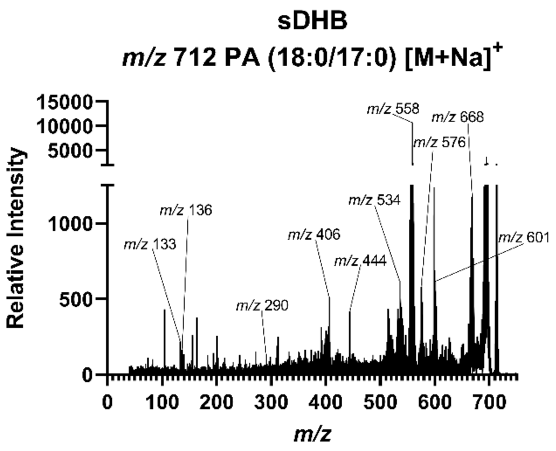
sDHB
m/z 712 PA (18:0/17:0) [M+Na]+
Relative Intensity
m/z
m/z 558
m/z 668
m/z 576
m/z 136
m/z 534
m/z 133
m/z 406
m/z 444
m/z 601
m/z 290

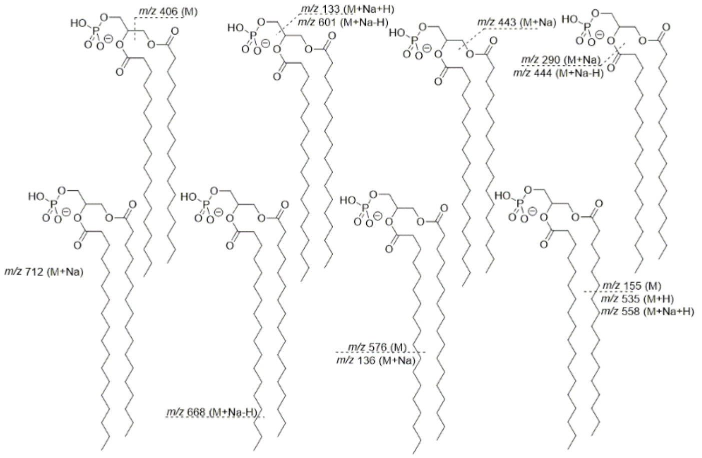
m/z 406 (M)
m/z 133 (M+Na+H)
m/z 601 (M+Na-H)
m/z 443 (M+Na)
m/z 290 (M+Na)
m/z 444 (M+Na-H)
m/z 712 (M+Na)
m/z 155 (M)
m/z 535 (M+H)
m/z 558 (M+Na+H)
m/z 576 (M)
m/z 136 (M+Na)
m/z 668 (M+Na-H)

HPA

***m/z* 715 PE (16:0/16:0) [M+Na+H]$^+$**

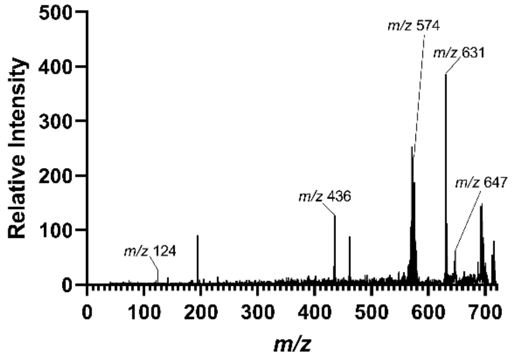

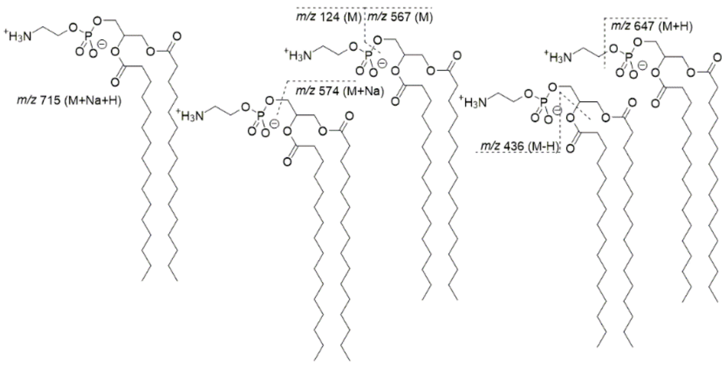

CMBZT

***m/z* 734 PE (16:1/16:0) [M+2Na-H]$^+$**

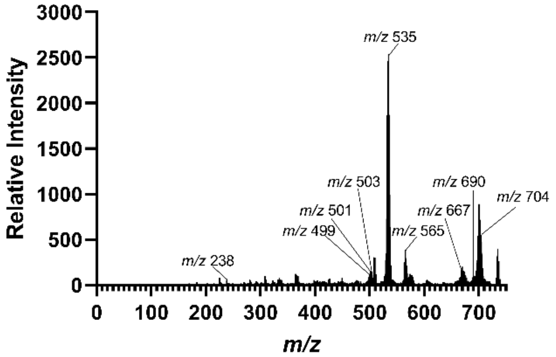

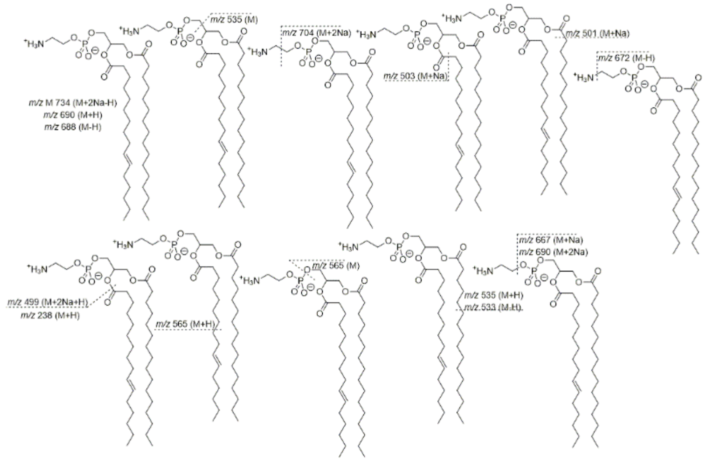

sDHB

***m/z* 740 PE (18:1/16:0) [M+Na]$^+$**

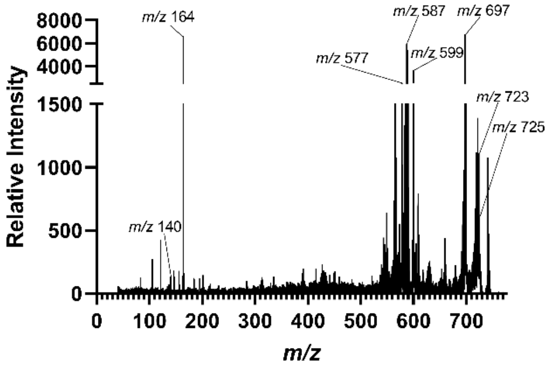

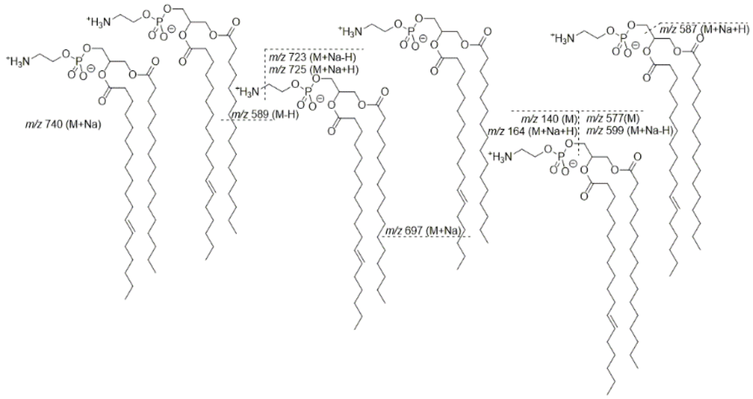

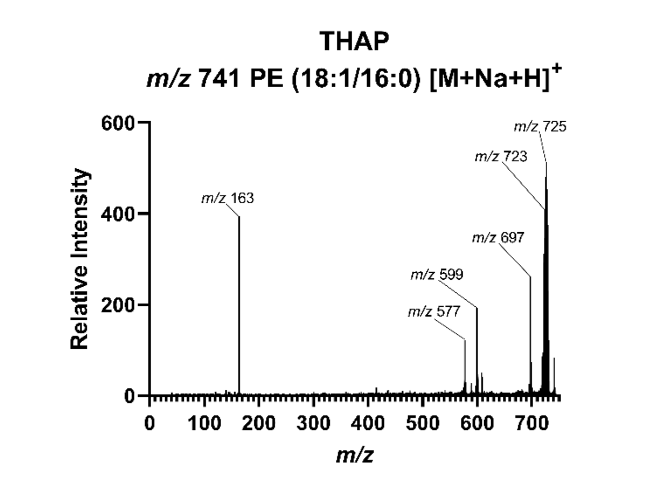
THAP
m/z 741 PE (18:1/16:0) [M+Na+H]+
Relative Intensity
m/z
m/z 725
m/z 723
m/z 697
m/z 599
m/z 577
m/z 163

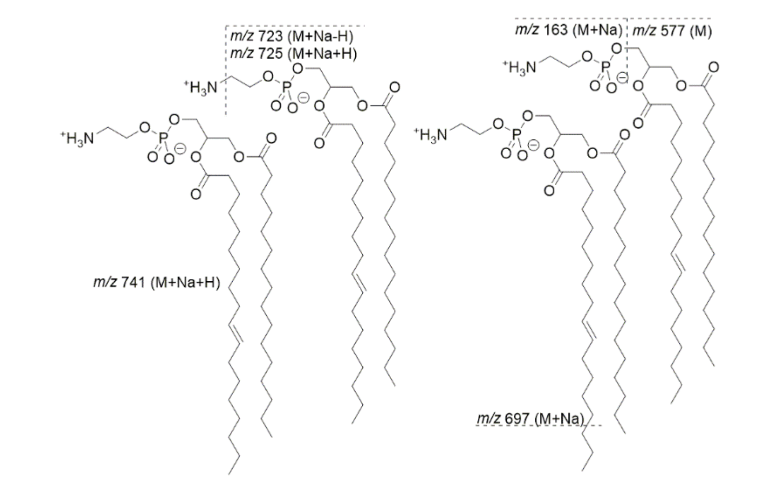
m/z 723 (M+Na-H)
m/z 725 (M+Na+H)
m/z 163 (M+Na)
m/z 577 (M)
m/z 741 (M+Na+H)
m/z 697 (M+Na)

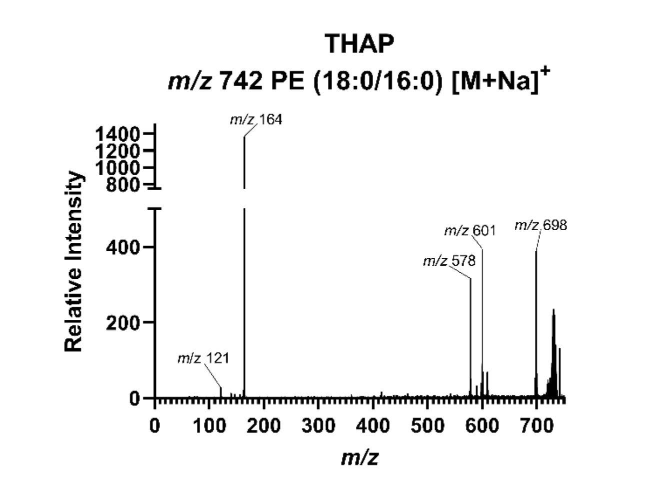
THAP
m/z 742 PE (18:0/16:0) [M+Na]+
Relative Intensity
m/z
m/z 164
m/z 601
m/z 698
m/z 578
m/z 121

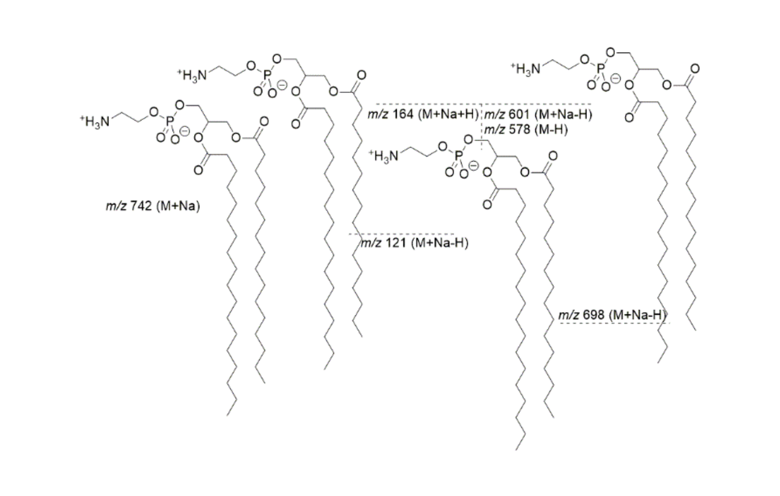
m/z 164 (M+Na+H)
m/z 601 (M+Na-H)
m/z 578 (M-H)
m/z 742 (M+Na)
m/z 121 (M+Na-H)
m/z 698 (M+Na-H)

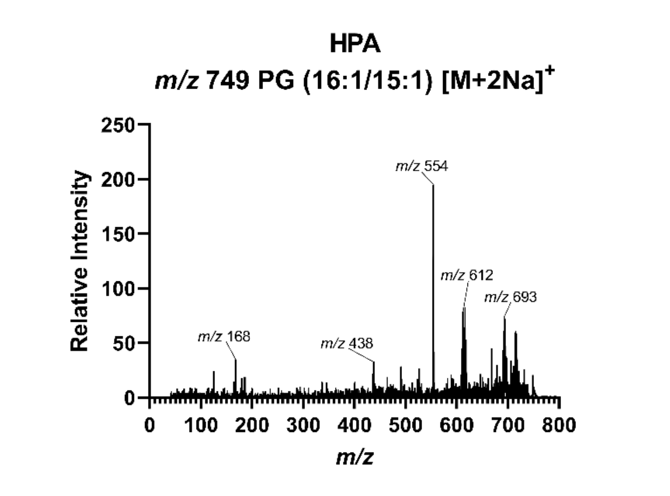
HPA
m/z 749 PG (16:1/15:1) [M+2Na]+
Relative Intensity
m/z
m/z 554
m/z 612
m/z 693
m/z 168
m/z 438

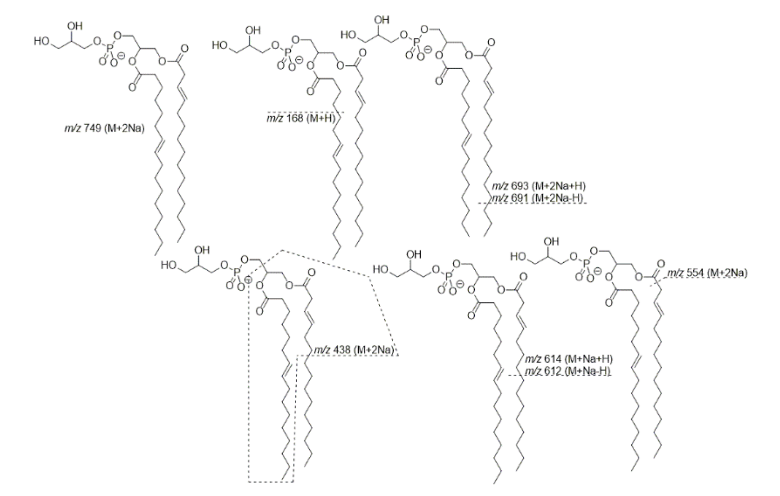
m/z 749 (M+2Na)
m/z 168 (M+H)
m/z 693 (M+2Na+H)
m/z 691 (M+2Na-H)
m/z 554 (M+2Na)
m/z 438 (M+2Na)
m/z 614 (M+Na+H)
m/z 612 (M+Na-H)

sDHB
***m/z* 754 PE (19:1/16:0) [M+Na]$^+$**

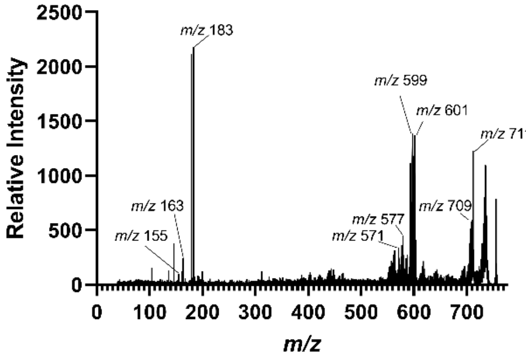

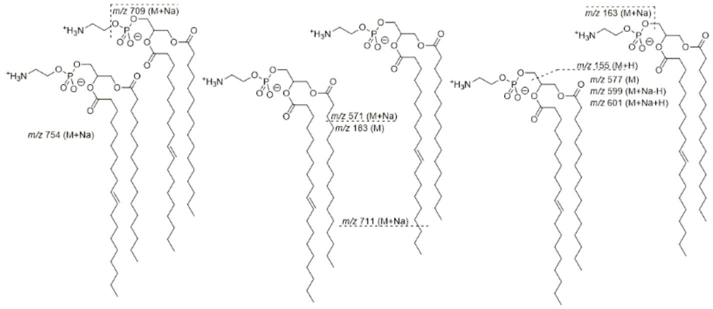

sDHB
***m/z* 756 Rha-Rha-C_{14}-C_{12} [M+Na-H]$^+$**

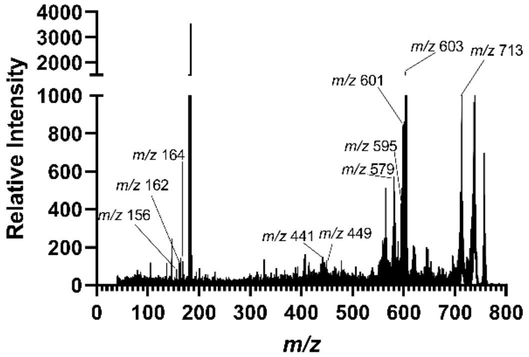

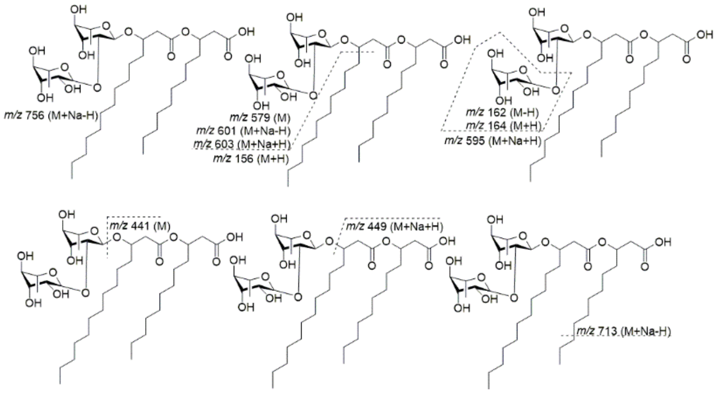

THAP
***m/z* 758 PE (18:2/16:1) [M+2Na-H]$^+$**

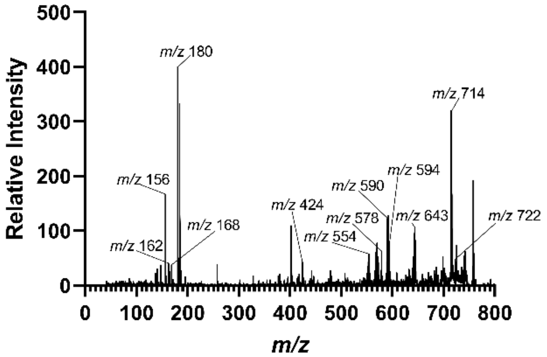

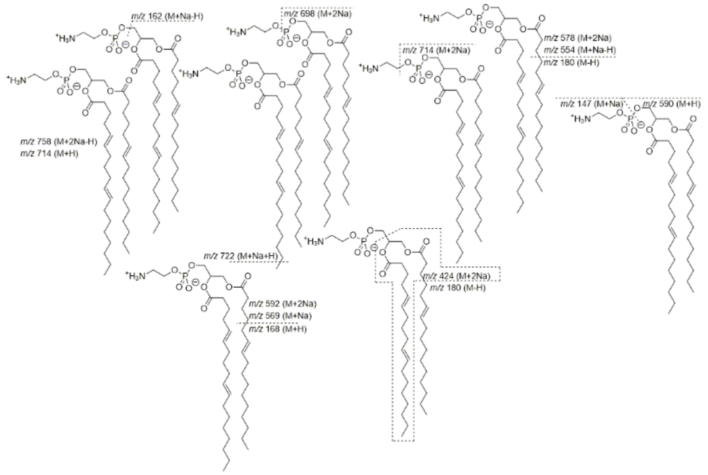

THAP

***m/z* 758 Rha-Rha-C_{12}-C_{14} $[M+Na+H]^+$**

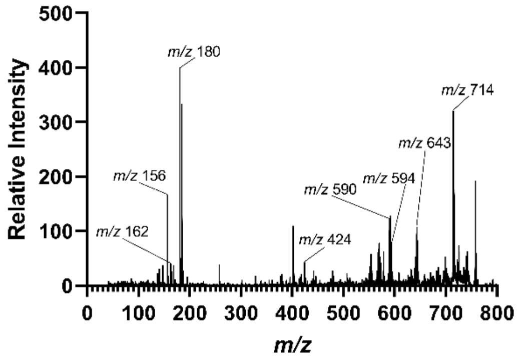

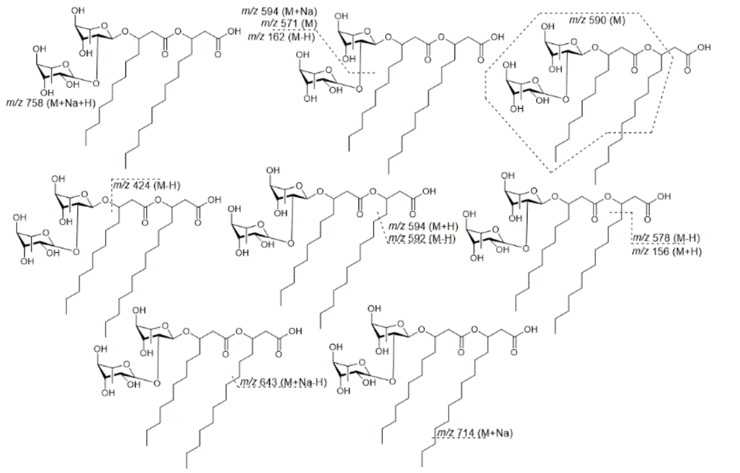

9AA

***m/z* 761 PE (18:1/16:1) $[M+2Na]^+$**

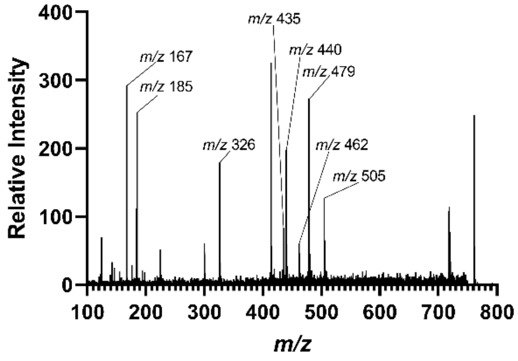

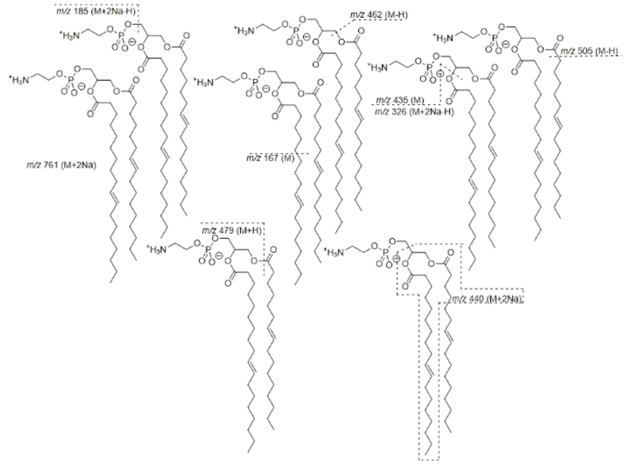

CMBZT

***m/z* 761 PE (18:2/16:0) $[M+2Na]^+$**

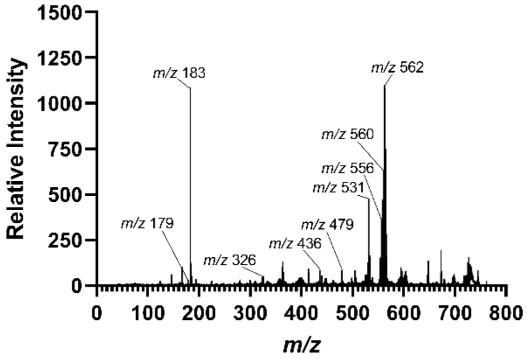

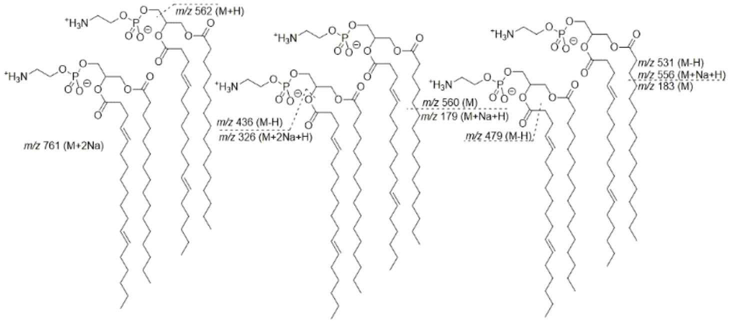

sDHB

***m/z* 761 PE (18:2/16:0) [M+2Na]$^+$**

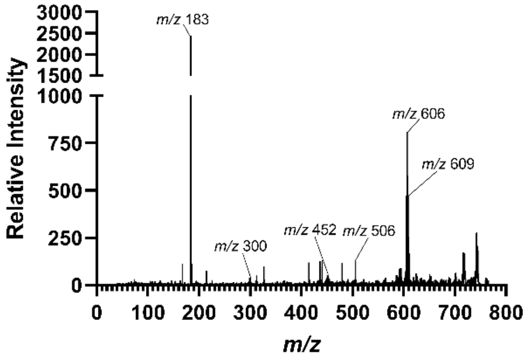

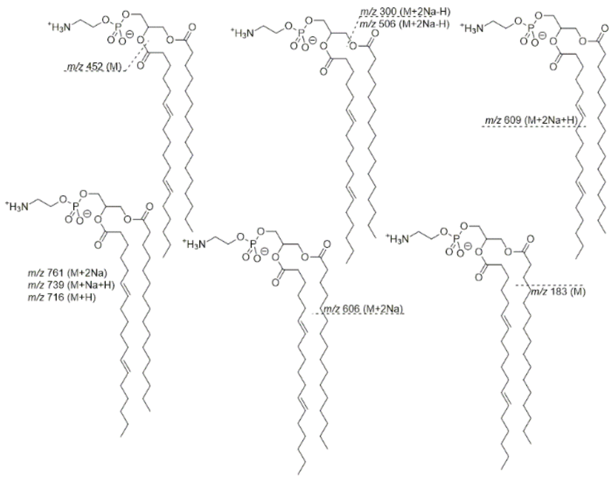

THAP

***m/z* 762 PE (18:2/16:0) [M+2Na+H]$^+$**

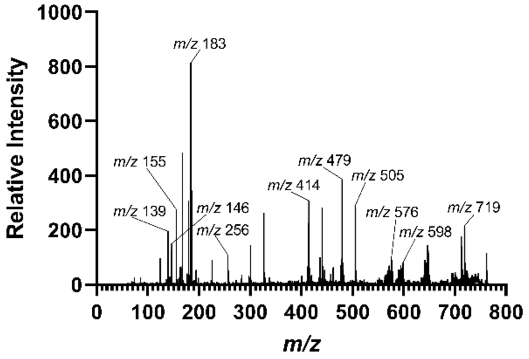

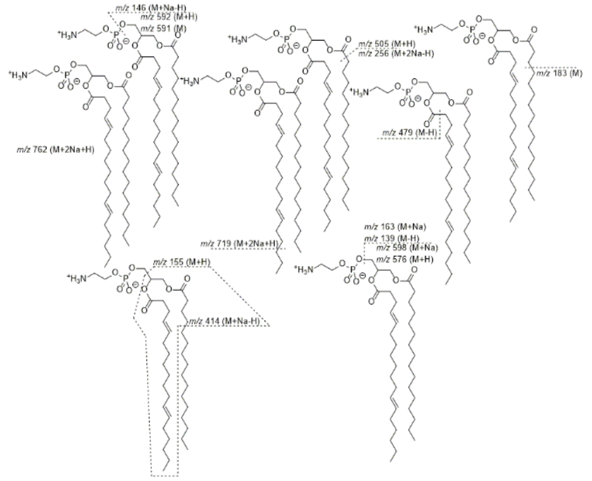

9AA

***m/z* 763 PE (18:1/16:0) [M+2Na]$^+$**

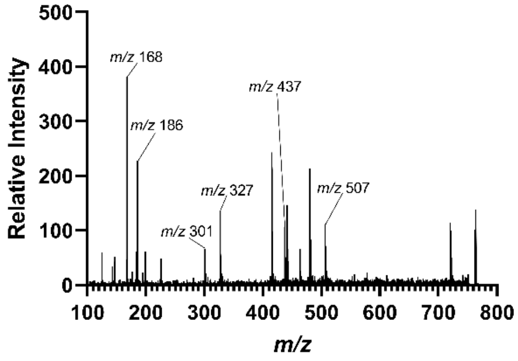

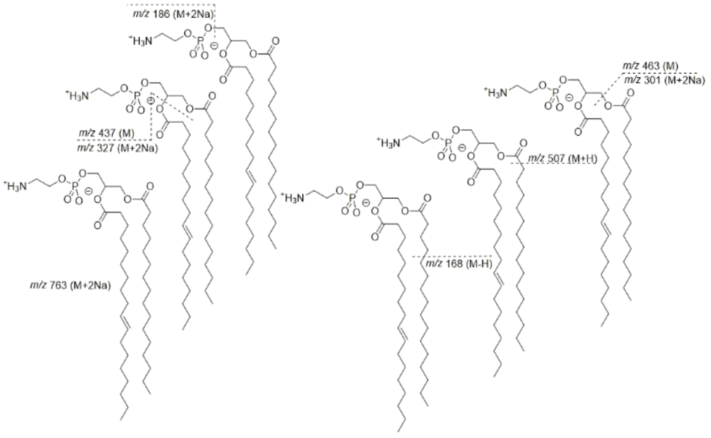

CHCA

***m/z* 763 PE (18:1/16:0) [M+2Na]$^+$**

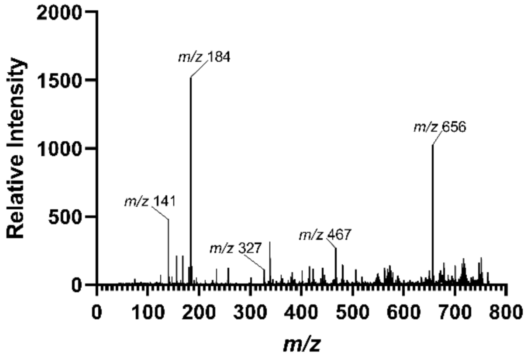

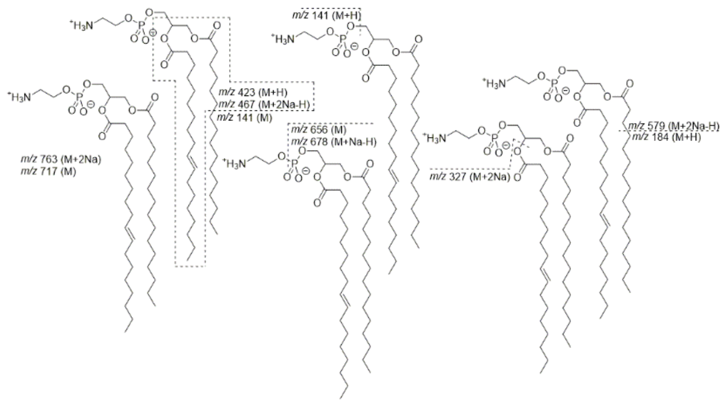

CMBZT

***m/z* 763 PE (18:1/16:0) [M+2Na]$^+$**

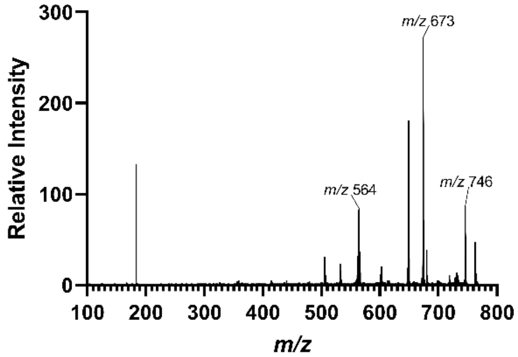

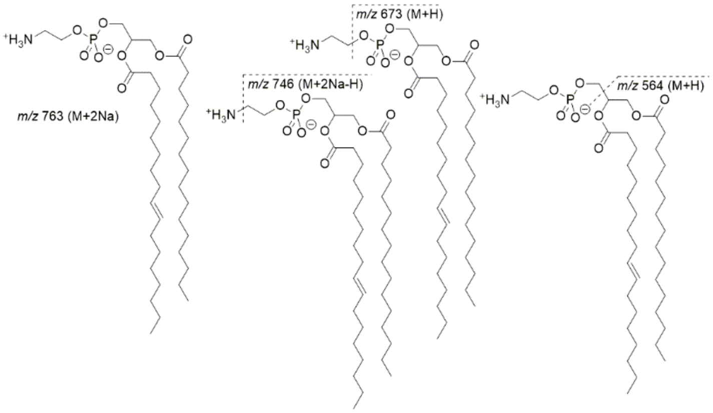

sDHB

***m/z* 763 PE (18:1/16:0) [M+2Na]$^+$**

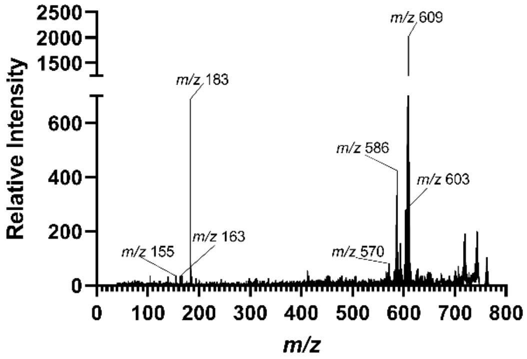

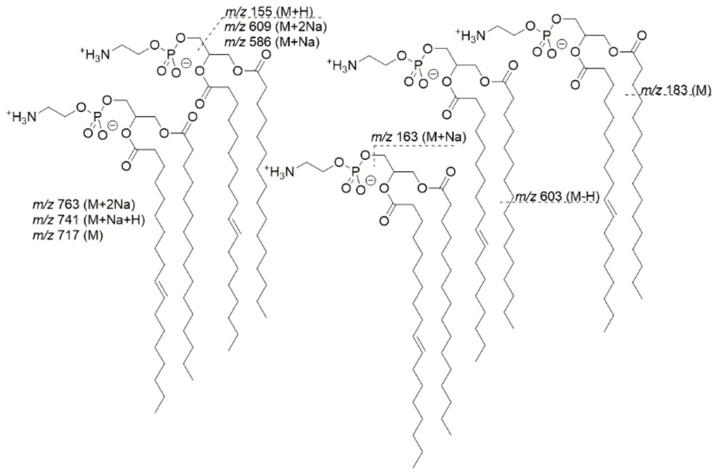

THAP

***m/z* 763 PE (18:1/16:0) [M+2Na]$^+$**

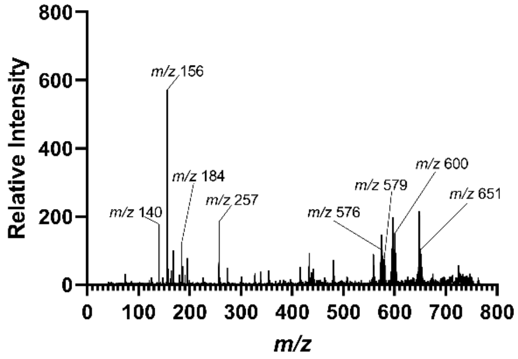

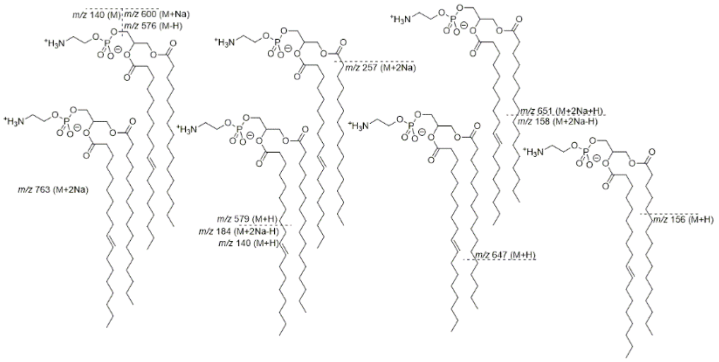

sDHB

***m/z* 776 PE (18:1/17:1) [M+2Na+H]$^+$**

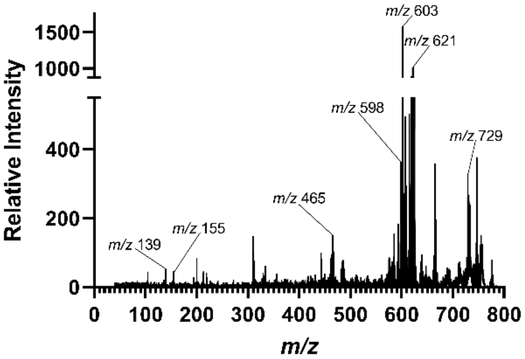

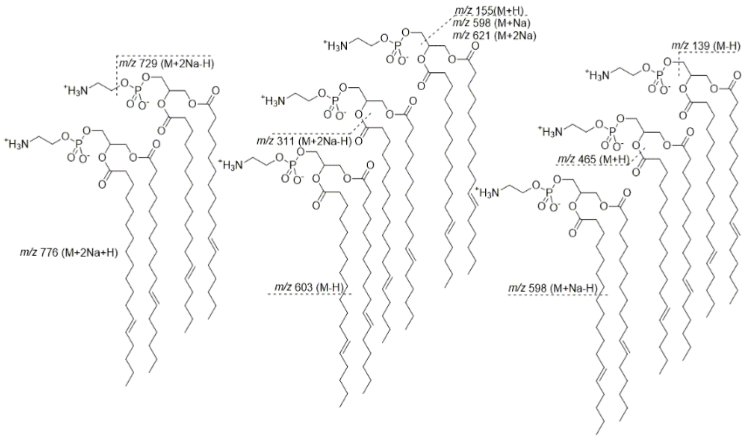

CHCA

***m/z* 779 PE (18:0/17:0) [M+2Na]$^+$**

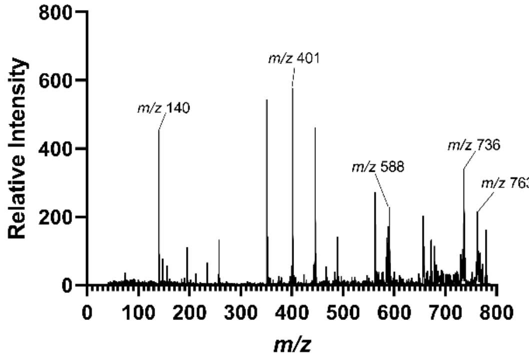

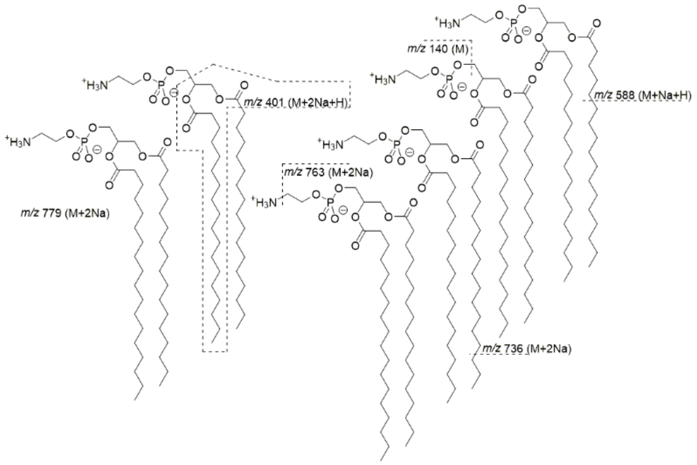

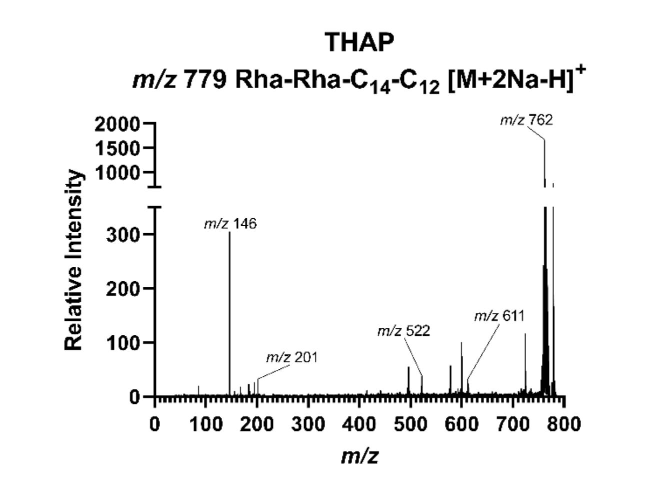
THAP
m/z 779 Rha-Rha-C14-C12 [M+2Na-H]+
Relative Intensity
m/z
m/z 762
m/z 146
m/z 201
m/z 522
m/z 611

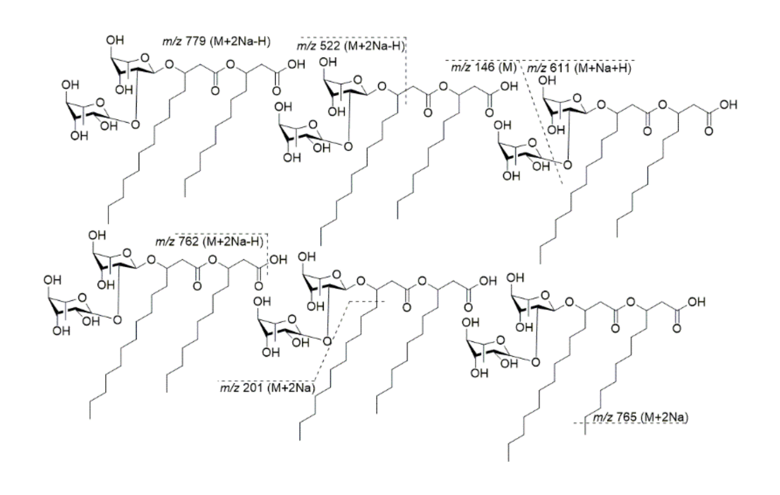
m/z 779 (M+2Na-H)
m/z 522 (M+2Na-H)
m/z 146 (M)
m/z 611 (M+Na+H)
m/z 762 (M+2Na-H)
m/z 201 (M+2Na)
m/z 765 (M+2Na)

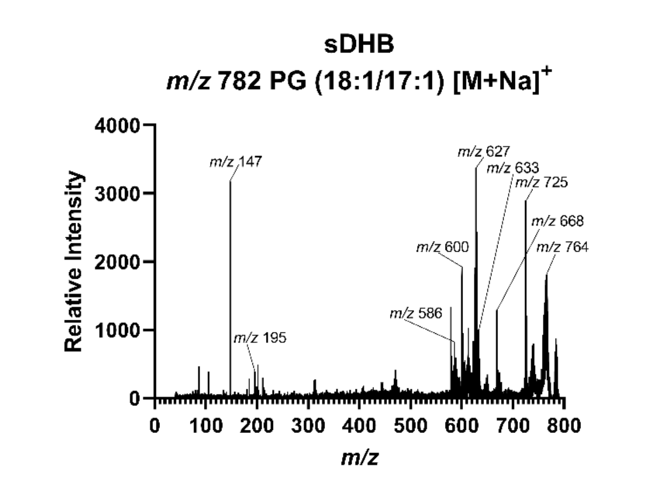
sDHB
m/z 782 PG (18:1/17:1) [M+Na]+
Relative Intensity
m/z
m/z 147
m/z 195
m/z 586
m/z 600
m/z 627
m/z 633
m/z 725
m/z 668
m/z 764

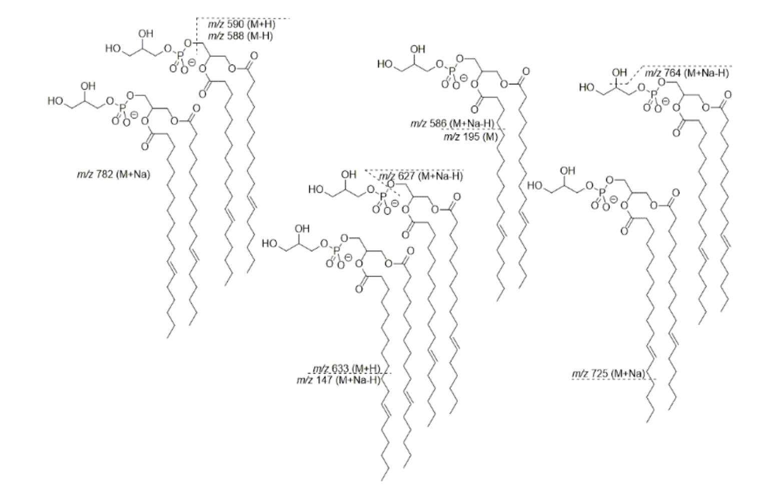
m/z 590 (M+H)
m/z 588 (M-H)
m/z 782 (M+Na)
m/z 586 (M+Na-H)
m/z 195 (M)
m/z 764 (M+Na-H)
m/z 627 (M+Na-H)
m/z 633 (M+H)
m/z 147 (M+Na-H)
m/z 725 (M+Na)

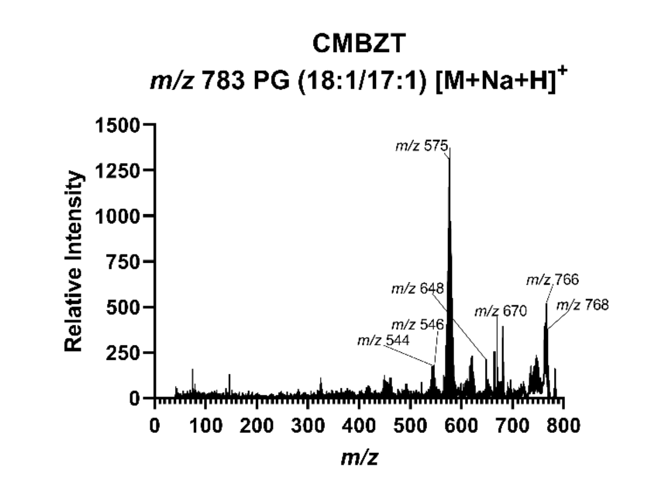
CMBZT
m/z 783 PG (18:1/17:1) [M+Na+H]+
Relative Intensity
m/z
m/z 575
m/z 648
m/z 546
m/z 544
m/z 670
m/z 766
m/z 768

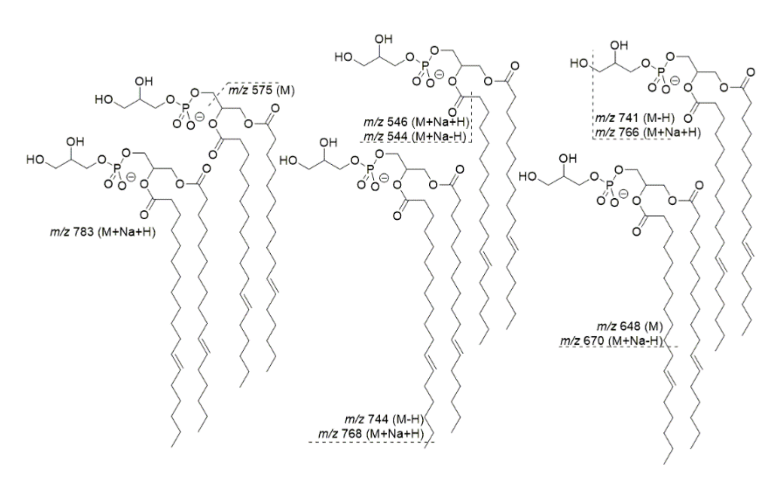
m/z 575 (M)
m/z 783 (M+Na+H)
m/z 546 (M+Na+H)
m/z 544 (M+Na-H)
m/z 741 (M-H)
m/z 766 (M+Na+H)
m/z 648 (M)
m/z 670 (M+Na-H)
m/z 744 (M-H)
m/z 768 (M+Na+H)

THAP

***m/z* 784 PG (18:1/17:0) [M+Na]$^+$**

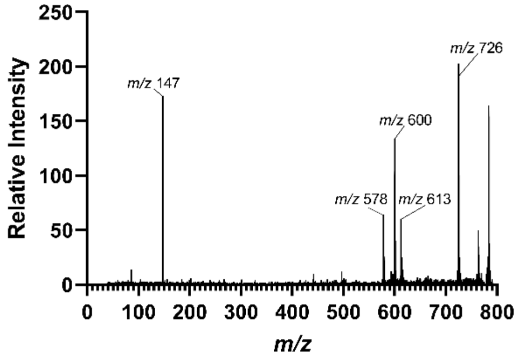

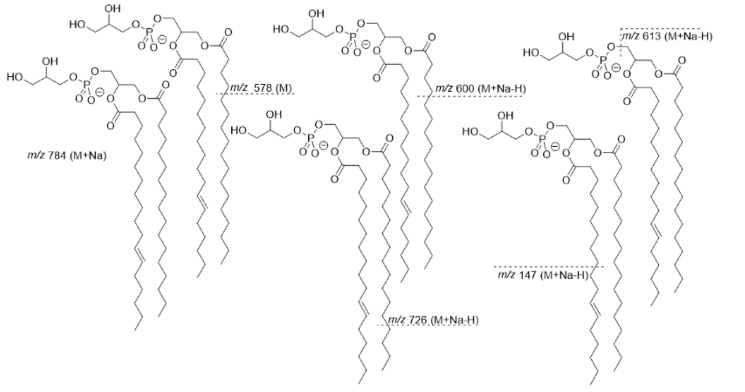

sDHB

***m/z* 787 PG (19:0/16:0) [M+Na+H]$^+$**

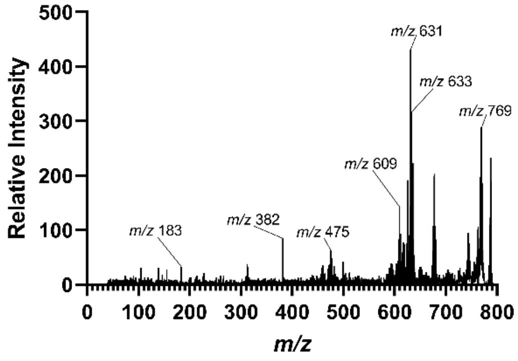

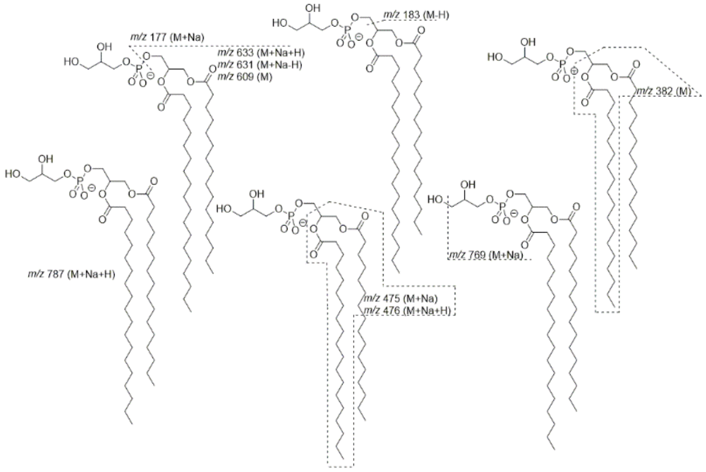

sDHB

***m/z* 792 PG (18:1/16:0) [M+2Na-H]$^+$**

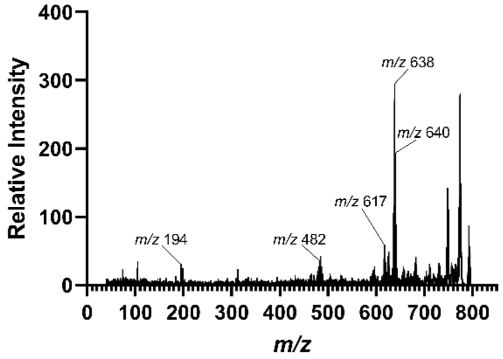

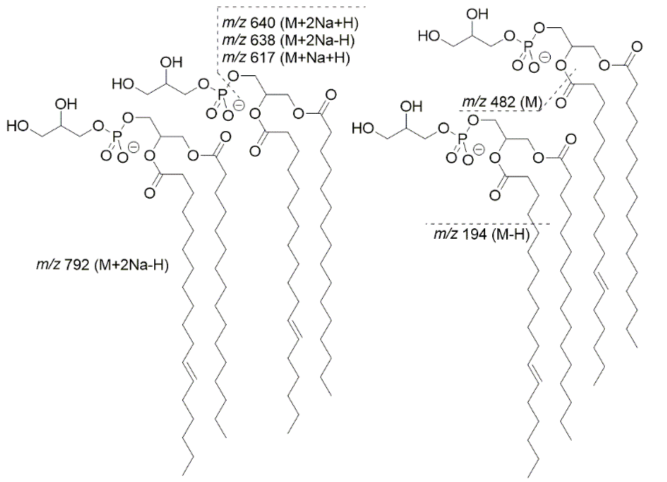

sDHB

***m/z* 798 PG (18:1/18:1) [M+Na+H]⁺**

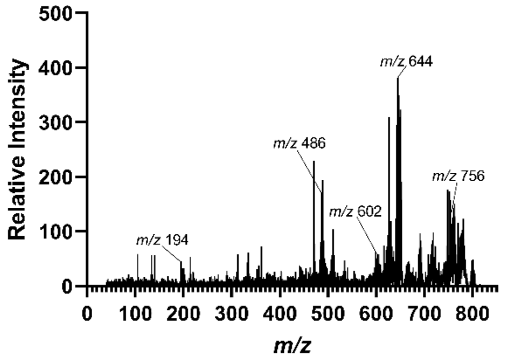

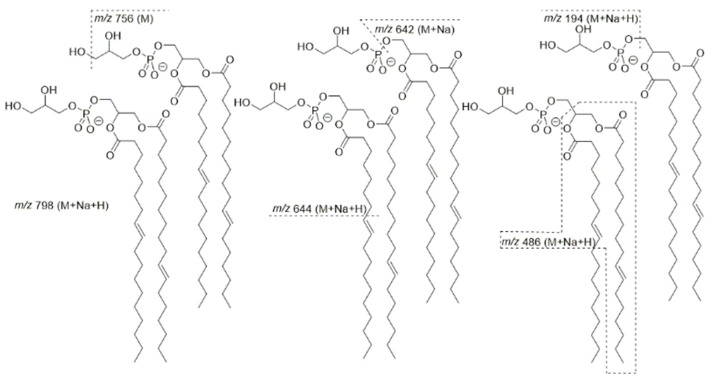

sDHB

***m/z* 809 PG (18:0/17:0) [M+2Na]⁺**

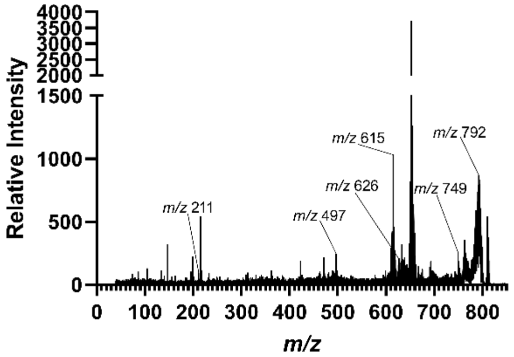

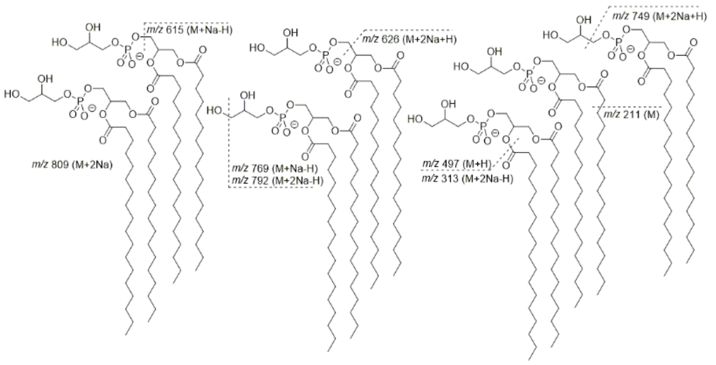

CHCA

***m/z* 813 PE (20:0/19:0) [M+Na]⁺**

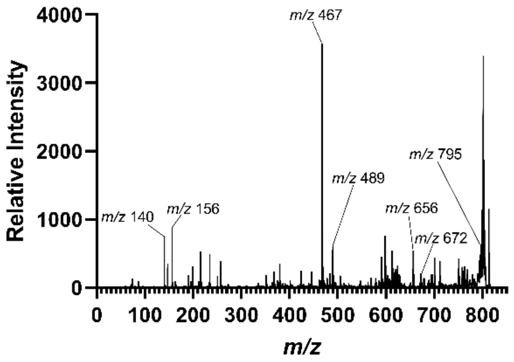

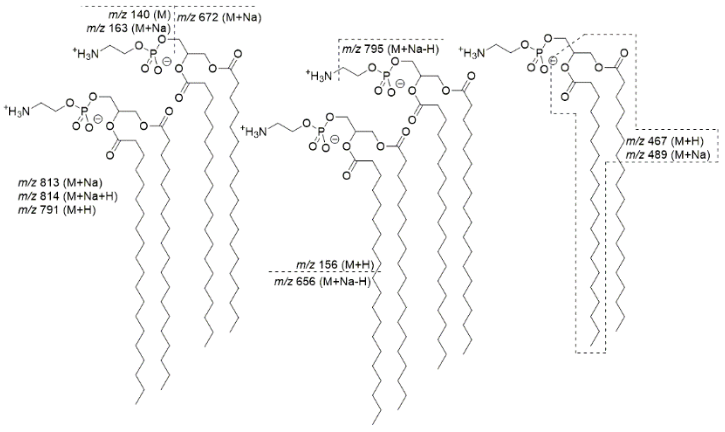

sDHB
m/z 820 PG (18:1/18:1) $[M+2Na+H]^+$

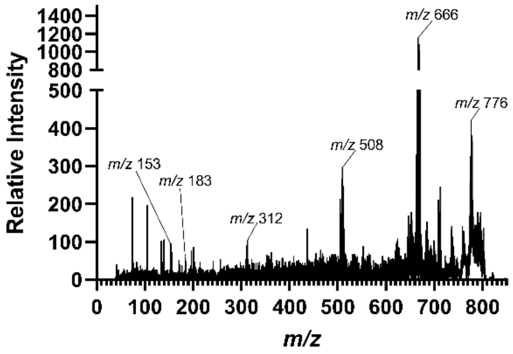

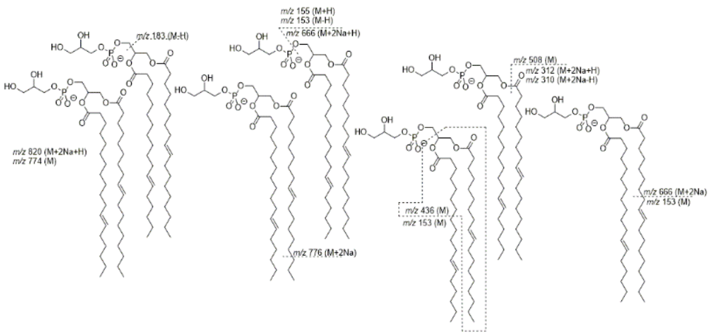

sDHB
m/z 827 Decenoyl-Rha-Rha-C_{10}-C_{10} $[M+Na+H]^+$

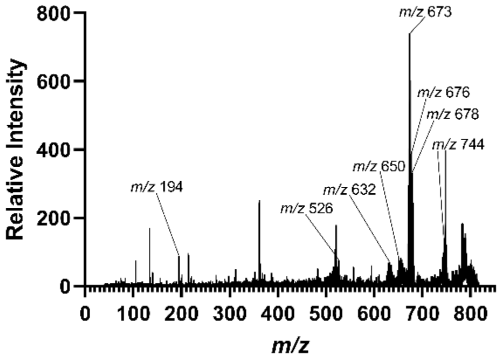

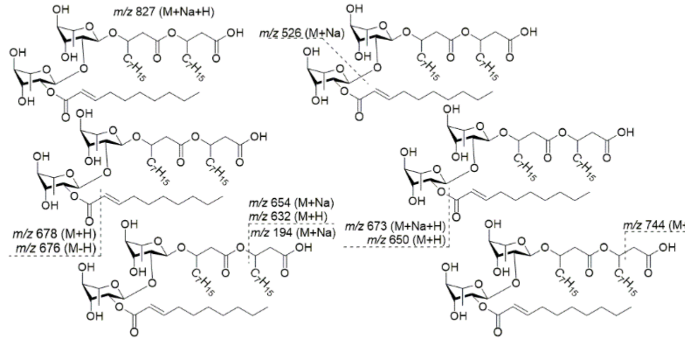

THAP
m/z 827 PG (19:1/19:0) $[M+Na+H]^+$

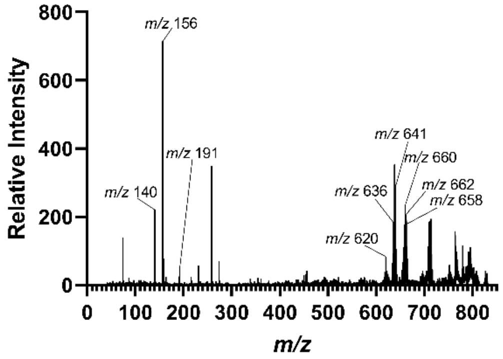

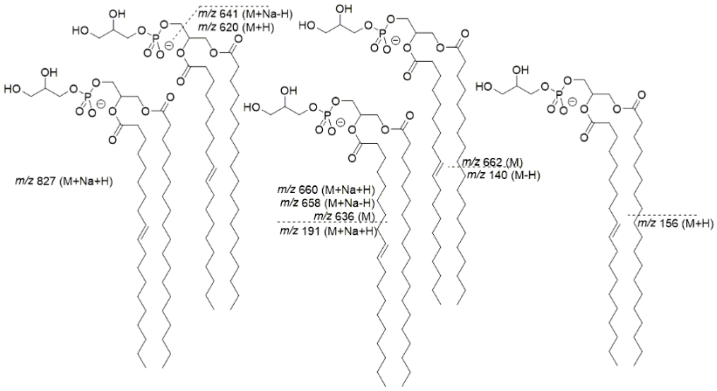

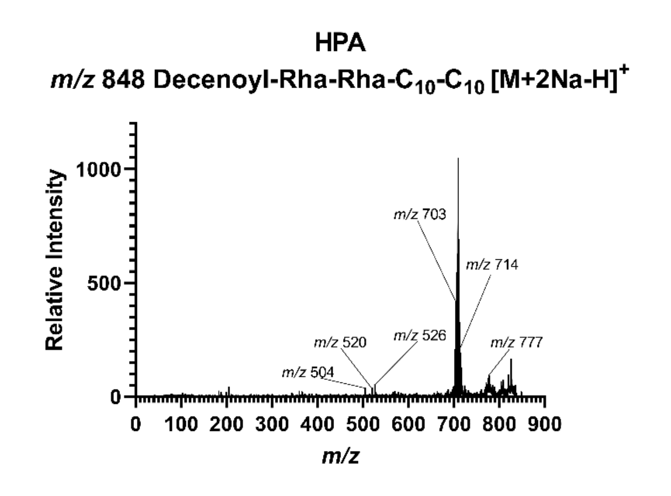
HPA
m/z 848 Decenoyl-Rha-Rha-C10-C10 [M+2Na-H]+
Relative Intensity
m/z
m/z 703
m/z 714
m/z 520
m/z 526
m/z 504
m/z 777

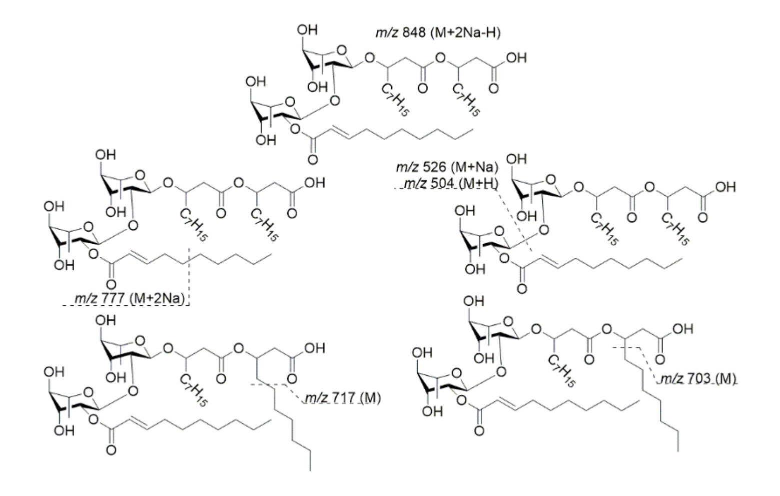
m/z 848 (M+2Na-H)
m/z 526 (M+Na)
m/z 504 (M+H)
m/z 777 (M+2Na)
m/z 717 (M)
m/z 703 (M)
